# Parents' Experiences and Reported Outcomes of Family‐Centred Care: A Qualitative Systematic Review

**DOI:** 10.1111/hex.70671

**Published:** 2026-04-19

**Authors:** Cansel Kocakabak, Agnes van den Hoogen, Melissa Rothfus, Marsha Campbell‐Yeo, Aurelia Abenstein, Anna Axelin, Patricia Schofield, Jos M. Latour

**Affiliations:** ^1^ School of Nursing and Midwifery, Faculty of Health University of Plymouth Plymouth UK; ^2^ Department Women and Baby, Neonatology, Wilhelmina Children's Hospital University Medical Centre Utrecht, Utrecht University Utrecht Netherlands; ^3^ Dalhousie Libraries Dalhousie University Halifax Nova Scotia Canada; ^4^ Faculty of Health Dalhousie University Halifax Nova Scotia Canada; ^5^ Global Foundation for the Care of Newborn Infants Munich Germany; ^6^ Department of Nursing Science University of Turku Turku Finland; ^7^ Department of Nursing, Zhongshan Hospital Fudan University Shanghai China; ^8^ Curtin School of Nursing Curtin University Perth Australia

**Keywords:** core outcome set, experiences, family‐centred care, neonatology, outcomes expectations, parents, qualitative research

## Abstract

**Introduction:**

Having an infant admitted to neonatal intensive care units (NICUs) is a distressing experience for parents. Family‐centred care (FCC) has been shown to improve outcomes for both infants and parents. However, there is inconsistency in reporting outcomes in FCC studies. The aim of this qualitative systematic review is to synthesise the experiences of parents related to FCC and to identify outcomes derived from these experiences to inform a Core Outcome Set (COS) for FCC in neonatal research and practice.

**Methods:**

This review included qualitative studies exploring parental experiences of FCC in NICUs. Databases searched were MEDLINE, EMBASE, CINAHL, Cochrane Library, PsycINFO, Scopus, JBI, Lilacs, and SciELO, completed in December 2022 and updated in May 2025. Included studies were critically appraised using the JBI checklist for qualitative research, and findings were synthesised using JBI meta‐aggregative approach.

**Results:**

From the 52 included studies, 275 findings were extracted. These findings were aggregated into four synthesised findings: (1) the impact of NICU admission on parental mental health; (2) becoming a Parent through caregiving involvement; (3) the influence of parent‐staff interactions on parental experience; (4) psychosocial and relational coping experiences of parent in the NICU and beyond. Outcomes were identified from each finding, reflecting parent, infants, and staff outcomes based on parental experiences.

**Conclusion:**

Parents' experiences of FCC in NICUs encompass both challenging and positive experiences, reflecting diversity in its implementation across settings. This highlights the need for studies FCC interventions to consider outcomes encompassing parents, infants, and healthcare professionals. This review informs the development a COS for FCC, ensuring that future research is relevant, effective, and aligned with parental experiences.

**Patient or Public Contribution**

This study is a qualitative systematic review of previously published literature and did not involve patients, service users, or members of the public in its design or conduct. However, the results of this qualitative systematic review are intended to inform a comprehensive and inclusive list of outcomes.

## 1 Introduction

It is estimated that approximately 13.4 million babies were born preterm worldwide in 2020, many of whom required admission to neonatal intensive care units (NICUs) [1]. While NICUs provide lifesaving and technologically advanced care for infants, having an infant admitted to a NICU can be emotionally distressing for parents [2]. Becoming a parent of an infant admitted to an NICU is often an emotionally and psychologically overwhelming experience [3], often resulting in adverse psychosocial and physical outcomes such as increased anxiety, depression [4], fatigue and sleep deprivation [5]. To better support parents experiencing these adverse outcomes, family‐centred care (FCC) has been implemented within NICUs [6]. Historically, neonatal care evolved from early home‐based infant care in the 19th century to highly medicalized hospital‐based system in the 20th century, which resulted in separation of infants from families [7]. Growing awareness of parental needs and consumer advocacy led to the development of FCC in NICUs [7].

The concept of FCC is considered as a philosophy of care [8], rather than a specific model of care. Conceptualising FCC as a model may be overly reductionist, as its principles are intended to remain flexible and adaptable to different settings, organisational cultures, and family's needs [9]. However, this flexibility has contributed to conceptual ambiguity and variability in how FCC is interpreted and implemented in clinical practice. Recently, a scoping review identified a range of FCC interventions designed to enhance parental experiences and neonatal outcomes [10]. Definitions of FCC changes across settings and countries [11], and there is no universally accepted definition of FCC. The most widely accepted definition is proposed by the Institute for Patient‐ and Family‐Centred Care (IPFCC), which describes FCC as an approach to the planning, delivery, and evaluation of healthcare grounded in partnership between families and healthcare professionals [12]. Despite the ongoing the ambiguity of definitions and interventions, FCC is recommended and endorsed by many organisations such as Global Foundation for the Care of Newborn Infants (GFCNI) [13], World Health Organisation (WHO) [14], and governments [15]. However, its conceptualisation, application, and interpretation across clinical settings around world remain broad, context dependent, and inconsistent [16].

A range of different FCC interventions have been shown to improve outcomes for both infants and parents [17]. However, the outcomes and outcome measurements reported in clinical trials are inconsistent and heterogeneous [18]. The variability in FCC definitions and interventions may contribute to inconsistency and heterogeneity in outcomes. Consequently, the lack of standardisation of FCC definitions, interventions, and outcome reporting limits meaningful comparison across studies and constrains synthesis of evidence needed to inform evidence‐based practice recommendations. To address this, it is essential to develop a core outcome set (COS) and measurements of FCC in NICUs [19]. A COS is an agreed minimum set of outcomes that should be measured and reported in clinical trials for a specific health condition [20]. The first stage in developing a COS involves identifying outcomes reported in previous studies [21].

We conducted a quantitative systematic review and found that most of infant outcomes were categorised within physiological area, while most parental outcomes related to emotional well‐being [18]. These results show that quantitative reviews often reflect outcomes chosen by researchers and clinicians [22] with limited patient and public involvement in the design and conduct of included studies. To ensure a comprehensive and inclusive list of outcomes, a systematic review of qualitative studies is essential to capture the experiences of parents. The aim of this systematic review is to synthesise the evidence of qualitative studies exploring the experiences of parents related to FCC and to derive outcomes from the synthesised findings to inform a COS for FCC in neonatal research and practice.

## 2 Methods

This systematic review is reported in accordance with the Enhancing Transparency in Reporting the Synthesis of Qualitative Research (ENTREQ) guideline [23] (Supporting Information S1: File 1) and registered in PROSPERO.

### 2.1 Search Strategy

A combination of MeSH terms and keywords related to neonatal care (e.g., special care ward, special care unit, critical care) and family‐centred care (e.g., patient‐centred care, professional family connections) informed the search strategy. No date limits were applied. The initial search strategy was developed in MEDLINE with a set of search terms and synonyms and then adapted to search in six other databases. This iterative process included the incorporation of new terms according to the search results. The full search strategy, along with the updated results, is provided in (Supporting Information S2: File 2).

### 2.2 Eligibility Criteria

**Population:** This review included studies that explored the experiences of parents and family members (mothers, fathers, family members, grandmothers, grandfathers) related to FCC interventions in neonatal settings. Studies that focused only on healthcare professionals' experiences related to the FCC were excluded.

**Phenomena of interest:** Studies that explored parents' and family members' experiences, views, perspectives, perceptions related to FCC in neonatal settings were included. Studies exploring experiences at least one component (collaboration, information sharing, participation, and partnership) of FCC defined by the Institute for Patient and Family‐Centred Care (IPFCC) were eligible [12]. Studies reporting follow‐up data after discharge related to FCC experiences were also included. Although Newborn Individualised Developmental Care and Assessment Programme (NIDCAP) and FCC interventions share common principles, NIDCAP is a structured developmental care intervention primarily focused on infant neurobehavioral regulation of care environment [24], whereas FCC represent a broader relational and organisational philosophy focuses on family partnership, collaboration, shared decision making. Therefore, studies testing NIDCAP were excluded to maintain conceptual clarity. **Context:** Studies conducted in neonatal care settings, which were NICU, newborn unit/ward, special care unit/ward, and critical care units admitting newborn infants were included. We excluded studies conducted in end‐of‐life care, palliative care, children and adults admitted to paediatric and adult intensive care settings.

**Study design:** Studies that focused on primarily qualitative design. Excluded were conference abstracts, dissertations, reviews, survey studies, quality improvement projects, and quantitative studies.

**Language:** Only studies published in English were included.

### 2.3 Information Sources

Medline (Ovid), Embase (Elsevier), CINAHL (EBSCO), the Cochrane Library, PsycINFO (EBSCO), Scopus (Elsevier), JBI EBP Database, LILACS (VHL), and SciELO (VHL) databases were searched for studies from beginning until 14 December 2022 and searches were updated on 30 May 2025.

### 2.4 Selection Process

Covidence systematic review software was used to manage screening process. All records identified through the databases search were imported into the Covidence systematic review software by the librarian (MR). Four reviewers (CK, AvdH, JML and MCY) independently screened titles and abstracts. If there was disagreement among reviewers, this was resolved through discussion. The full‐text screening was undertaken by two reviewers based on the eligibility criteria (CK and AvdH). Any disagreements were resolved through discussion with a third reviewer (JML).

### 2.5 Risk of Bias Assessment

The risk of bias of chosen studies was assessed using the JBI Critical Appraisal Checklist for qualitative research [25]. Studies were assessed by two reviewers (JML and CK). None of the studies were excluded based on the quality assessment.

### 2.6 Data Extraction

Data were extracted in a data extraction form: authors, years, country, aim, data collection method, population, analysis and main findings. A summary of the extracted data is presented in Table 1, with full details of provided (Supporting Information S3: File 3).

**Table 1:** Characteristics of the studies included in the systematic review.

Author year	Aim	Population	Main findings
Shafey et al., 2022	Experiences fathers of FICare	Fathers (*n* = 13)	Fear of the unknown; Mental preparation; Identifying fathers ‘role; Parenting with supervision; Effective communication; Post neonatal care transition; Family life
Abukari et al., 2022*	Experiences families of FCC	Family (*n* = 42)	Family experiences of FCC practices
Dien et al., 2022	Experiences mothers of FICare	Mothers (*n* = 26)	Recovering from birth; Adapting to the NICU; Caring for baby; Coping with daily disruption; Seeing progress; Supporting parenting
Ferreira et al., 2021	Experiences parents in care	Mothers (*n* = 9) Father (*n* = 1)	Parent‐staff interaction; Supportive/trustworthy HCPs; Consistency in care; Family couple and peer support; Newborn status; Resources and education; NICU environment; Research participation
van den Hoogen et al., 2021	Experiences parents of FCC	Mother (*n* = 11) Fathers (*n* = 2)	Involvement in care; Personalised information and communication; Transition to a parental role; Emotional support
Ndiaye et al., 2020	Experiences parents about primary carers	Mothers (*n *= 10) Fathers (*n* = 2)	General impressions of the neonatal unit; Communication; Maternal stress
Neu et al., 2020	Experiences mothers of FCC	Mothers (*n* = 14)	Visiting; General caregiving; Holding; Feeding; Maternal ideas for improvement
Lundqvist et al., 2019	Experiences parents of FCC	Parents (*n* = 6)	Feelings of existential loneliness and guilt; Challenges in becoming a mother; Ambivalent relationship to partner; Professionals supportive; Being unprepared coming home; Neonatal home care‐return to everyday life; Alignment partner needs; Stressed about everyday hassle; Father's bonding process
Sarin et al., 2019	Experience parents of FCC	Family (*n* = 12)	Acceptability of FCC, access to child, and improved well‐being of child; Interaction between staff and parent attendants was based on helpful communication; Empowerment and self‐efficacy of parents
Maastrup et al., 2018	Experience parents of SSC	Mothers (*n* = 9) Fathers (*n* = 2)	Overcoming ambivalence through professional support; Proximity and parental feeling; Feeling useful and realising the important of skin‐to‐skin contact; Bonding is beneficial regardless of survival
Serlachius et al., 2018	Experiences parents of FCC	Mothers (*n* = 63) Fathers (*n* = 20)	Disempowerment; Hierarchy between parents and staff; Father's peripheral role
Broom et al., 2017	Experiences parents of FICare	Mothers (*n* = 4) Grandmother (*n* = 1)	Benefits of FICare components; Enhancement of parent confidence and parental role attainment; Improved parent‐parent communication
Stephanie al., 2017	Experiences parents of closeness/separation	Mothers (*n* = 13) Fathers (*n* = 7)	Having a role as a parent; Providing for and getting to know the infant; Support from staff; Reluctantly leaving the infant's bedside; NICU environment
Brødsgaard et al., 2015	Experiences parents of early discharge	Parents (*n* = 9)	Respect and understanding of family's overall situation; A natural progression; Level of information before and during EDP; Participation and dialogue before/during EDP; Recommendation of EDP
Weis et al., 2015	Experiences parents of GFCC	Parents (*n* = 22)	Discovering and expressing emotions; Reaching a deeper level of communication; Obtain mutual understanding
Russel et al., 2014	Experiences parents of FCC	Mothers (*n* = 32) Fathers (*n* = 7)	Parental involvement; Staff competence and efficiency; Interpersonal relationship with staff
Finlayson et al., 2014	Experiences mothers of FCC	Mothers (*n* = 12)	Mothering in limbo; Deference to the experts; Anxious surveillance; Muted relations; Power struggles; Consistently inconsistent
Shirazi et al., 2016*	Experiences caregivers of FCC	Mothers (*n *= 6) Grandmother (*n* = 1)	Meta‐family interaction; Comprehensive support; Reconstruction of a normal family
Lundqvist et al., 2007	Experiences fathers of caring	Fathers (*n* = 13)	Feeling of distance; Feelings of proximity
Sarapat et al., 2017	Experiences parents of care	Family (*n* = 24)	Uncertainty about child's condition; Desire to be close to babies; Lack of confidence in providing care; Overcoming difficulties in breastfeeding; Socio‐cultural factors influencing parental involvement
Ingram et al., 2017	Experiences parents of FCC discharge	Parents (*n* = 37)	Practical preparation: knowledge and skills transfer; Emotional preparation: uncertainty, feeling rushed, motivation to get home; Role of feeding: breastfeeding is the harder way to do it
Shirazi et al., 2018	Experiences caregivers of FCC	Family (*n* = 8)	Restoring stability; Oriented coalition; Dynamics of care; Empowering the family
Mӧrelius et al., 2021	Experiences fathers of feeding	Fathers (*n* = 7)	Shared responsibility for feeding process; A long and demanding process
Mohammadi et al., 2020	Experiences of mothers of dignity	Mothers (*n* = 20)	Privacy; Respecting and individual identity; Authority
Patriksson et al., 2019	Experiences of parents of the presence of language barrier	Parents (*n* = 10)	Wanting to speak for oneself; Being aware of cultural keys; Understanding one another in employees' arena
Axelin et al., 2018*	Experiences parents of decision‐making	Parents (*n* = 22)	Collaborative communication decision‐making; Neonatologist‐led communication decision‐making; Emergency communication decision‐making; Disconnected communication decision‐making
Reis et al., 2010*	Experiences parents of negotiated partnership	Parents (*n* = 10)	Perceptive engagement; Cautious guidance; Subtle presence
Heermann et al., 2005	Experiences mothers of becoming a mother	Mothers (*n* = 15)	From NICU to baby; From their baby to my baby; From passive to active; From silence to advocacy
Wigert et al., 2014	Experiences parents of communication with staff	Parents (*n* = 27)	Meeting a fellow human being; Being included or excluded as a parent; Bearing unwanted responsibility
Gotting et al., 2022	Experiences parents of pictorial support for communication	Family (*n* = 31)	Communicating through pictorial support; Facing barriers in communication; Facing external influences; Need for a good healthcare relationship
Jafari et al., 2023*	Experiences parents of communication	Mothers (*n* = 5)	Mutually ineffective relationship between personnel and parents
Afeadie et al., 2023	Experiences mothers of information and interactions	Mothers (*n* = 15)	Socioeconomic statues of participants; Healthcare workers' mood at specific time; Physicians in possession of detailed information
Hajiaraghi et al., 2021*	Experiences parents of strategic elements of FCC	Mothers (*n* = 3) Fathers (*n* = 2)	Family and care; Parental characteristics in care; Family needs
Petersson et al., 2021	Experiences parents of family health conversations	Family (*n* = 12)	Co‐creating comprehensive picture; Feeling validated; Feeling equipped for future
Sigurdson et al., 2020	Experiences former parents of FCC	Parents (*n* = 18)	Family lack of knowledge; Staff judgement; Unmet family‐identified need for nurse continuity of care and meaningful relationship with nurse; Inconsistent access to quality translation service
Dahan et al., 2023*	Experiences parents of information sharing	Parents (*n* = 10)	Revolving clinicians; Controlling narrative; Impact on relationship; Impact on personhood
Park et al., 2024*	Experiences parents of FCC	Parents (*n* = 3)	Wandering; Becoming a harmonious team
Shrestha et al., 2025	Experiences parents of care and support	Mothers (*n* = 20) Fathers (*n* = 5)	Diverse experience of care and support; Initial care involvement; Care involvement outcomes
Nukpezah et al., 2025	Experiences mothers of care	Mothers (*n* = 13)	Maternal anxiety about unknown outcomes of newborn condition; Positive impact of family‐centred care; Maternal roles for preterm care; Poor support for maternal involvement in care
Guttmann et al., 2024	Experiences parents of communication quality	Mothers (*n* = 8) Fathers (*n* = 6)	Strengths; Challenges; People; Coping strategies
Eriksson et al., 2024	Experiences fathers of HBNHC	Fathers (*n* = 12)	Vivid memories from NICU; Struggling with new challenges in life; Transition to home still their thoughts
Stefana et al., 2024	Experiences fathers of nurses' role and care practices	Fathers (*n* = 20)	Communication and clarity about infants' health condition and progress; Inclusiveness and guidance from nurses; Fathers' satisfaction with nurses' support for mother; Nurses' personal attention to the babies; Nurses' varied personalities.
Cai et al., 2024	Experiences parents of KC	Mothers (*n* = 12) Fathers (*n* = 3)	Low motivation upon initial engagement with KC; Dynamic fluctuations of emotional states during KC; Unexpected gains; Barriers to participation.
Jafari et al., 2024*	Experiences parents of barriers of FCC	Mothers (*n* = 5)	Inefficiency of playing parental role; Ineffective involvement of parents in care
Brødsgaard et al., 2024	Experiences shared parents of separation	Parents (*n* = 8)	Becoming parents at different paces; Being at juncture between separation and closeness
Osborne et al., 2024	Experiences parents of communication	Parents (*n* = 16)	Medical team inclusion and connection; Confusion regarding NICU care; Discharge readiness; Methods of communication
Schmid et al., 2024*	Experiences parents of parental presence	Parents (*n* = 20)	Structural factors of the institution; Organisation and time management; Resources; Physical and psychological aspects; Parent‐professional interaction; Cultural aspects and language
Kabajassi et al., 2024*	Experiences parents of adaptation of FICare	Mothers (*n* = 10)	Healthcare provider's workload would be reduced by offloading tasks to mothers; Neonatal outcomes would be improved by involving mothers in patient care; Mothers would be empowered by assuming more responsibility; Maternal stress would increase with increased responsibility; Mothers would not be able to learn new skills; Healthcare providers would not trust maternal assessment
Banazadeh et al., 2024*	Experiences parents of decision‐making	Parents (*n* = 10)	Parental capabilities; Parental self‐efficacy; Conviction; Living conditions
Franck et al., 2024	Experiences parents of mFICare	Mothers (*n* = 9) Fathers (*n* = 1)	Actively caring for the infant; Learning how to care their infant; Learning about the clinical status of their infant
Ondusko et al., 2025	Experiences Black parents of family support	Mothers (*n* = 10) Fathers (*n* = 3)	Distrust and fear of medical setting; Hypervigilance and trauma trajectory formation; Myth of Black hardiness; Policing and surveillance; Undermining Black parenting; Earn rather than assume trust; Respect family concerns; Improve mental health support; Provide compassionate care; Support parenting role
Ajayi et al., 2024	Experiences Black mothers of communication and support	Mothers (*n* = 12)	Maternal/nursing care experiences; Interactions in NICU; Support needs

### 2.7 Synthesis Methods

The JBI meta‐aggregative approach was used to synthesise the qualitative data. This analysis method consists of three stages. In the first stage, findings were extracted from each included study. Each finding was then assessed for credibility using JBI's established criteria: unequivocal, credible, or unsupported. In the following stage, findings with similar meaning were grouped into categories based on similar experiences. These categories were developed with a focus on outcomes to ensure relevance to the development of a COS. However, due to the limited number of categories permitted within meta‐aggregative approach, some outcomes may not have been fully captured within the category structure. To address this, Table 2 was created to present the derived outcomes from each category. At the final stage, categories were aggregated to generated broader, overarching synthesised findings, each representing a collective understanding of parents' experiences in FCC.

**Table 2:** Identified outcomes from synthesised findings.

Parent outcomes	Infant outcomes	Staff outcomes
**Parental emotional and mental health** Anxiety, stress, depression, guilt, worry, self‐blame, separation, secondary trauma, anticipatory grief, sleep deprivation, fatigue, emotional exhaustion, loneliness and isolation, emotional expression, emotional relief	Length of NICU stay	Staff competence
**Parental identity, role and confidence** Parental role, maternal role, paternal role, disconnection from maternal role, parental identity, parental responsibility, maternal self‐esteem, parental empowerment, parental dignity, parent respectful care, confidence in breastfeeding	Infant fragility and medical instability	Staff support
**Participation and involvement** Parental participation, parental Involvement, participation in care, perceived involvement in care, duration of parental presence, parental involvement frequency, father involvement in care, FCC participation, shared decision‐making, decision‐making involvement, decisional conflict, perceived discrimination in care	Feeding adequacy	Parent‐staff partnership
**Communication and partnership with staff** Parent communication, parental reassurance, parental hesitation to seek support, parental trust in healthcare providers, satisfaction with communication, parental perception of staff emotional support, parental perception of staff practical support, parental perception of staff informational support, relational closeness with staff	Developmental immaturity	Parent‐staff interaction
**Knowledge, preparedness and advocacy:** Knowledge of NICU environment, discharge readiness, parental preparedness for preterm birth, parental advocacy	Mortality	Staff collaboration with parents
**Comfort, support and social well‐being** Satisfaction, satisfaction with caregiving role, satisfaction with overall care, parental relationship satisfaction, perceived social support	Acute deterioration	Staff recognition of family expertise
**Family functioning and relationship** Parent‐infant bonding, mother‐infant bonding, father‐infant attachment, detachment, attachment, family functioning	Post‐discharge needs	Staff permission and guidance
	Reduced behavioural stress	Staff familiarity
	Infant's safety	Staff responsiveness to parental input
	Feeding methods at discharge	Staff encouragement
	Infection rates	Parent‐staff communication
	Physical regulation	Staff acceptance of religious or cultural practices

## 3 Result

### 3.1 Study Selection

**Figure 1:**
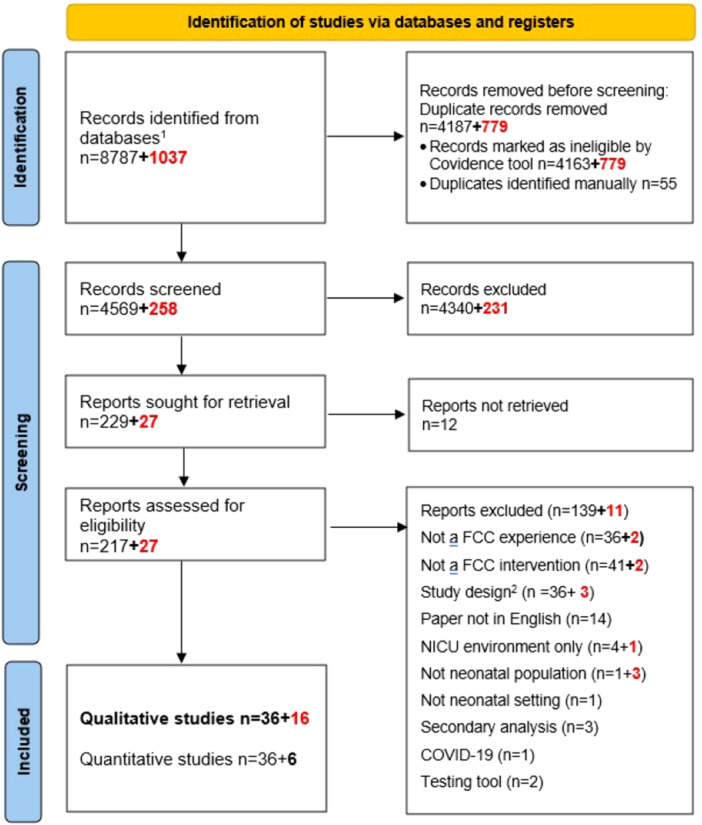
Flow diagram of study selection in December 2022 and updated in May 2025 [1]. Databases are MEDLINE, EMBASE, CINAHL, Cochrane, PsycINFO, Scopus, Johanna Briggs Institute (JBI), Lilacs, SciELO [2]. Study design not related to the inclusion criteria such as survey studies, quality improvement project, implementation project, poster, systematic review, and quantitative design. Numbers in bold are related to the update search in November 2023. Abbreviation: FCC, family‐centred care.

The search strategy retrieved a total of 9824 studies. After duplications were removed, 4571 out of 4827 studies were excluded following title and abstract screening. The remaining 244 studies were undertaken for full‐text assessment. We excluded 150 studies based on the eligibility criteria. Consequently, we included 52 studies [26, 27, 28, 29, 30, 31, 32, 33, 34, 35, 36, 37, 38, 39, 40, 41, 42, 43, 44, 45, 46, 47, 48, 49, 50, 51, 52, 53, 54, 55, 56, 57, 58, 59, 60, 61, 62, 63, 64, 65, 66, 67, 68, 69, 70, 71, 72, 73, 74, 75, 76, 77] (Figure 1).

### 3.2 Study Characteristics

The characteristics of the included studies are summarised in Table 1 The studies were published between 2005 and 2025 and included a total of 834 participants. Of the included studies, all, except two, were published after 2010. These studies were conducted across 20 countries spanning five continents (North America, Africa, Europe, Asia, Oceania), with most studies originating from Europe. The epistemological approaches included phenomenology, exploratory descriptive, interpretive descriptive, qualitative descriptive, qualitative deductive, descriptive comparative, hermeneutic phenomenology, grounded theory, and ethnography. The included studies explored various aspects of parental FCC experiences.

### 3.3 Risk of Bias

All studies met criteria 3 and 10 of the JBI Critical Appraisal Checklist for Qualitative Research which assessed the congruity between the research methodology and data collection, as well as alignment between data interpretation and conclusions. Only one study [44] did not meet the criteria 4 and 5, related to the congruity between the research methodology and the representation and analysis of data, and the interpretation of results. Most studies did not meet criteria 6 and 7, which assess whether the researcher' position was considered culturally or theoretically, and the potential influence of researcher on research, and vice‐versa.

### 3.4 Review Findings

A total of 275 findings were extracted from the 52 included studies [26, 27, 28, 29, 30, 31, 32, 33, 34, 35, 36, 37, 38, 39, 40, 41, 42, 43, 44, 45, 46, 47, 48, 49, 50, 51, 52, 53, 54, 55, 56, 57, 58, 59, 60, 61, 62, 63, 64, 65, 66, 67, 68, 69, 70, 71, 72, 73, 74, 75, 76, 77] and assessed for its credibility. According to the JBI's established criteria: Unequivocal (U), Credible (C), and Unsupported (US), four findings were considered as unsupported, and 11 findings as credible. The remaining 260 findings were deemed unequivocal.

All findings were grouped into 19 categories based on similarity in meaning and were further aggregated into four synthesised findings. The four synthesised findings are: ‘The Impact of NICU admission on Parental Mental Health’, ‘Becoming a Parent through Caregiving Involvement’, ‘The Influence of Parent‐Staff Interaction on Parental Experience’, and ‘Psychosocial and Relational Coping Experiences of Parent in the NICU and Beyond.’ A detailed list of extracted findings with supporting quotations, categories, and synthesised findings, is presented in Supporting Information S4: File 4. Furthermore, derived outcomes from each finding along with its quotation, category, and synthesised finding is presented in Supporting Information S5: File 5. Although this review primarily focused on parents' experience of FCC, we also derived infant and healthcare professional outcomes (Table 2).

### 3.5 Synthesised Finding 1: ‘The Impact of Nicu Admission on Parental Mental Health’

This synthesised finding was developed from 37 findings across four categories, exploring the emotional and psychological impact of NICU hospitalisation on parents. Of these findings, 19 parental and 7 infant outcomes were derived (Supporting Information S5: File 5).

#### 3.5.1 Parental Fear and Worry Related to the Infant's Health and Survival

Parents described persistent fear and worry from admission to discharge. Initially, parents expressed fear related to the uncertainty and the possibility of losing their infant. Afterwards, it shifted to fear of harming their infants due to complex medical equipment. As one mother stated, ‘… it was like, you rather not touch him because you thought then I'll rip off this or that tube or something like that. So, it was a bit, it was hard [38]. As discharge approached, parents expressed worry about their infant's feeding and caregiving readiness. As one mother reported, ‘I never stopped pumping my breasts even a single time because I'm worried that if my baby starts getting milk, I would have no breast milk for her… So I try to express milk every 2‐3 h to keep my milk… [34].

#### 3.5.2 Feelings of Isolation and Guilt Due to Separation from the Infant

Mothers reported loneliness and lack of support from family members. One mother described, *‘*Because of poverty, my husband cannot find the ticket (transportation fare) to come visit me every day. My father is too old to move around. My mother is no longer alive' [36]. Mothers also reported blaming themselves for giving birth prematurely and leaving their infants in the NICU. One mother expressed, ‘I feel a sense of abandonment and I have to leave earlier than planned so I am feeling a little guilty too’ [29].

#### 3.5.3 Stress Related to Parental Vulnerability and External Caregiving Environment

This category reflects variety stress factors experienced by parents, including concern about their own health, financial constrains related to medical expenses, lack of information and communication, daily responsibilities, and monitor sounds and alarms in the NICU. Parents reported difficulties managing house works and caring for other siblings. While visiting NICUs, one father expressed ‘Felt that it took a lot of energy to keep up with everything at home, visit them in the NICU and make sure that the older children did not suffer’ [39]. In addition, parents reported feeling overwhelmed due to the NICU environment, where constant alarms and noises heightened their anxiety. One mother stated, ‘When the red light is flashing and that alarms going off, I'm having palpitations wondering why nobody was doing anything’ [41].

#### 3.5.4 Supportive Information and Preparation to Reduce the Emotional Toll of the Nicu Experience

Parents reported that being informed in advance about the possibility of premature birth helped them feel less overwhelmed when their infant was admitted to NICU. Furthermore, parents highlighted the need for structured education session to introduce them to the NICU environment, expectations, and available resources. As one parent stated, ‘There should be some sort of way to present to parents, this is the NICU, this is what you can expect kind of thing, like, just one information session. Parents should be invited to one information session’ [42].

### 3.6 Synthesised Finding 2: Becoming a Parent through Caregiving Involvement

This synthesised finding was developed from 81 findings across four categories. It reflects how caregiving involvement in the NICU contributes to development of parental identity, autonomy, and bonding, and how these experiences are shaped by the institutional conditions. A total of 26 parental and five infant outcomes were identified (Supporting Information S5: File 5).

#### 3.6.1 Developing Parental Role and Identity through Involvement

Parents experienced difficulties related to their expectation of being a mother or father when their infant was admitted to the NICU. They reported the feelings of role confusion, disconnection with their role, a sense of inadequacy and detachment, particularly fathers reported feelings of not visible their role, and not treated as an equal parent.

Parents experienced strong desire to take their parental role and feel themselves as real parents by performing caregiving tasks. As one mother stated, ‘We did the care all by ourselves. It was our own process and very meaningful for us to feel complete as a parent’ [28] The act of feeding, holding, and changing diapers strengthened their parental identity. As one parent stated, ‘You can feel a little more like mum and dad when you have him skin‐to‐skin–not a guest in his home’ [37].

#### 3.6.2 Parental Autonomy and Self‐Efficacy

Parents gradually gained caregiving skills, which led to increased confidence and self‐efficacy. When parents had restricted access to information, they felt lack of control. However, when they were involved in making‐decisions in their infants' care, they reported feelings of autonomy. As one parent said, ‘For a brief moment you feel like you are in control again, which for me gives me the sense of responsibility and bonds me to my baby’ [29], Initially, parents were scared when involved in their infant's care, but in time their caregiving skills developed naturally. As one parented reported, ‘Although initially it was scary, I developed the skills, it was a natural progression; we learned how to take care of him' [45].

#### 3.6.3 Parent‐Infant Bonding through Caregiving

Parents reported when their contact was limited, they experienced delayed and uncertainty in bonding with their infant. One father said, ‘I had not had much time with the infant, and therefore ended up thinking “now what”? [39]. Conversely, parents who experienced proximity to their infants bonded emotionally and physically. ‘We have to go home every day and come in twice a day …. Our presence with our baby. We want to be close. For nearly everything’ [31].

#### 3.6.4 Influence of Nicu Environment and Policies on Caregiving Involvement

This category reflects how the NICU environment and hospital policies either supported or constrained parental involvement. When the NICU environment and facilities focused on supporting only mothers, fathers were often being treated as secondary and as a supporting role within the couple. The nature of the NICU environment was overwhelming and distracting for parents, one parent stated, ‘Preoccupied by my surroundings and sometimes [I] would lose the ability to focus on the time I was spending with my son’ [29]. Furthermore, hospital policies were reported to actively facilitate mothers’ involvement, ‘As a dad, there's food supplied for Emily but not for me. In terms of encouraging dads to be here it makes it harder …. As dad, we don't have much to do but support mum. The thought is put into supporting mum but not supporting the people who support mum’ [34].

### 3.7 Synthesised Finding 3: The Influence of Parent‐Staff Interactions on Parental Experience

This synthesised finding is developed from 121 findings across eight categories. It highlights the vital role healthcare professionals play in shaping parents' trust, caregiving skills, involvement, communication. A total of 57 parental outcomes and seven healthcare professional outcomes were identified (Supporting Information S5: File 5).

#### 3.7.1 Inclusive Communication and Recognition of Parents during Medical Rounds

When healthcare professionals listened to parents' observations of their infant, asked for their opinions, and encouraged them to ask questions during bedside and medical rounds, parents felt recognised, respected, and empowered; ‘It's very easy if they talked at the bedside of the baby, then you're obviously part of the conversation, and they always asked me at the end of their discussion, they asked me if I had any questions and concerns’ [42]. Effective communication between parents and healthcare professional was valued for parents. A mother reported ‘It was great to get that interaction, especially with the doctors, because they included you as part of the rounds, whereas I found before that they would kind of look at you but not talk to you’ [44].

#### 3.7.2 Encouragement and Coaching to Support Parental Caregiving

This category shows the encouragement and guidance of healthcare professionals supporting parents when they performed caregiving tasks and were involved in their infant's care. This resulted in more positive experiences, feelings more empowered, confident, happy and connected to their infants. They also reported overcoming initial fears of caring for their infant, embracing their parental role, and seeking further involvement, and regaining sense of normalcy.

Healthcare professionals' encouragement provided reassurance to parents. As one mother explained, ‘She kept willing us forward. ‘Why don't you try this? Why don't you do his nappy?’ I was like ‘Oh no’, very scared, but very encouraged to touch him, talk to him… Things around us were very positive’ [33]. Similarly, with healthcare professionals’ encouragement another mother learned how to care in her infants ‘The NICU nurse encouraged us to participate in the care. We learned a lot by observing how nurses cared and by copying their practice’ [28].

#### 3.7.3 Staff Familiarity and Presence Supporting Parental Involvement

When healthcare professionals were consistently present during parents' caregiving process, it contributed to strengthened parental independence, comfort, and a sense of security. As parents spent more time in the unit, they became more with familiar with each other, which leading to feelings of supported and understood; ‘Sometimes you just can't see any nurses. It is comfortable, when you can see a few’ [37]. Even when parents would like to have greater responsibility for their infant's care, knowing that healthcare professionals were nearby provided reassurance; ‘Tell me, don't you want us to help you? I say I will get it myself, but I see them looking at me from a distance to look out for me. So, I have nothing to worry about when I need help. Her nurse is there to help me’ [26].

#### 3.7.4 Parental Trust in Staff Expertise and Supervision

Parents expressed trust in knowledge, skills, and clinical experiences of healthcare professionals, particularly during the early and critical phase of their infant's hospitalisation. Parents' trust was rooted in the perceived expertise and professionalism of healthcare professionals. As one mother stated, ‘All the doctors that were there, as far as I'm concerned, were the experts. The doctors had, you know, been in this industry for like 15, 20, 25 years. They knew, they knew their stuff inside out you know, the information was never flaky…’ [33]. Furthermore, witnessing improvements in their infant's health with healthcare professionals guidance further reassured parents and fostered emotional relief; ‘The biggest joy was seeing how quickly my son was able to overcome the obstacles of being preterm. He was able to start eating a lot better under their [nurses’] supervision and as a result he got stronger. That was a great joy to see him get so much stronger so fast under their care’ [43].

#### 3.7.5 Staff Communication and Explanation to Promote Parental Understanding

Clear and consistent communication from healthcare professionals was important in helping parents understand medical procedures, monitor readings, and discharge planning. When staff allocated time to explain process detail and provided opportunities to ask questions, parents felt valued; ‘It was one of the nursery nurses sat down with me and explained what it [Train‐to‐Home] was and did the first round of stickers and then another nurse went through it again a bit later, updated it with a different discharge date and everything’ [40]. Conversely, inadequate and unclear communication led to parents' feeling unsecured and less confident like one father reported, ‘I was more or less happy with the care but felt that the staff did not devote enough time to informing them and making sure that they had understood the information’ [39].

#### 3.7.6 Individualised Emotional Support and Respectful Care

When healthcare professionals provided individualised emotional support based on parent's needs, they felt valuable and respectful. As one mother described, ‘We talked all the time [in the unit], but that was different. We discussed subjects at a deeper level [in the dialogues]. Yes, we discussed totally different subjects than in the unit everyday life’ [27]. Emotional support from healthcare professionals was expressed as an emotional recovery. One parent explained ‘It was nice to tell them my story and to have somebody who was just listening and who understood the situation on the NICU’ [28].

#### 3.7.7 Staff Behaviours That Undermine Parental Confidence and Emotional Safety

Parents reported feelings of marginalisation and disempowerment when healthcare professionals excluded them from caregiving activities, such as acting as gatekeepers, or restricting access to treatment information. These experiences contributed to a perception of being passive recipients of care rather than active participants in their infant's caregiving. As one mother expressed, ‘We felt not to bother them like kindergarten children. But it would've made us more confident and comfortable [31] ’. This exclusion led to hesitate seek help, especially for emotional concerns; ‘I wouldn't talk to them [nurses] about feeling upset or depressed or if I had worries about her or anything, in case they thought I was some kind of psychotic mother. I've never felt…. I just couldn't speak to them about anything personal or anything like that’ [41].

#### 3.7.8 Inconsistency in Staffing and Its Impact Parental Experience

Parents described difficulties resulting from a lack of continuity among nurses and doctors. Frequent changes in healthcare professionals disrupted communication and caregiving plans, leading to feelings of insecurity and diminished trust in healthcare team. As one parent described ‘The one thing we found that was a little bit hard was when there would be a new nurse, it was like starting again from scratch. They would assume our knowledge was very low and would repeat a lot of the same thing or try to tell us the same thing… So the more they could keep the same person [nurse] with us, the easier it was and more beneficial it was for us’ [43]. Changes in doctors also affected parents' confidence in the stability of care plans. As another parent shared, ‘If the same physician was in for 2 weeks, you get momentum going and you would kind of be—you'd have a plan, more of a long‐term plan. When someone would come in on the weekend, sometimes they would totally switch gears on you. And then, whoever was there would come back the next week, it would sometimes shift again’ [42].

### 3.8 Synthesised Finding 4: Psychosocial and Relational Coping Experiences of Parent in the NICU and Beyond

This synthesised finding is aggregated from three categories and draws on 35 findings. It reflects that the ways in which parents cope with the psychological, emotional, and relational challenges of having a baby in the NICU and transitioning to life after discharge. A total of 19 parental outcomes were identified (Supporting Information S5: File 5).

#### 3.8.1 Coping Strategies and Psychosocial Support

Parents described the experience of having a baby born prematurely as emotionally overwhelming and chaotic. When they expressed their feelings to each other, they felt better and more understood: ‘[Using reflection sheets] helped us put into words some of the things we actually felt inside. It felt good to express some of the chaos, because it is chaotic having a baby born that early’ [27]. Parents who received peer and online support found it helpful in overcoming difficulties. However, parents expressed a need for their partner's physical presence and for professional mental health support. As one parent emphasised, ‘There should be a team to comfort mothers because many of them arrive depressed and they don't know how to pick themselves up’ [36].

#### 3.8.2 Family and Partner Dynamics in the NICU

This category shows the varied partner dynamics while caring their infant. Even though some couples expressed fatigue, supporting each other strengthened their relationship. Conversely, some couples experienced tensions in their relationship; ‘she felt that her partner did not understand how it affected her not being able to sleep at night’ [39]. Extended family support, particularly guidance how to care their babies, as one grandmother expressed, ‘I just told her (the preterm baby's mother) how to take a bath and what kind of milk to feed a baby… She should pour some water on the face first to prevent a baby from getting cold… Be careful not to wet a baby's umbilical cord stump as it might get infected…’ [34] grandmothers helped parents during challenging time.

#### 3.8.3 Support for Transitioning from NICU to Home Care

Parents described the transition from the NICU to home. Parents received support during transition, which helped them feel better prepared and reassured that their infants could continue to receive appropriate care after discharge. However, parents experienced mixed emotional and practical responses. While some parents reported feelings of independent and comfort, as one mother said, ‘Coming home was liberating and a step back into normal life, with more independence’ [39], another parent reported feeling emotionally overwhelmed and unprepared for the responsibilities, ‘Then I start to cry. It's like filling the house again after it's been empty’ [38].

### 3.9 Outcome Identification

The aim of this qualitative systematic review was to derive outcomes based on parental experiences of FCC in neonatal settings. According to the COMET handbook, an outcome is defined as a measurement or observation used to capture and assess the effect of an intervention [21]. As this review will inform the development of a COS, outcomes were operationally defined as experiential, relational, or perceived changes described by parents that reflect meaningful impacts associated with FCC implementation. Using the JBI meta‐aggregative approach, we synthesised findings from 52 included studies into four overarching synthesised findings and associated categories. As part of this process, outcomes were systematically derived from parents' narratives within included studies. These outcomes were based on both explicitly reported experiences and analytically interpreted patterns within grouped categories (Supporting Information S4: File 4). Although many outcomes were identified across multiple categories, each outcome is presented only once in Table 2 to avoid duplication.

The derived outcomes were then grouped into three domains: parent, infant, and staff outcomes (Table 2). Parental outcomes reflect a wide range of emotional, relational, and experiential dimensions, such as mental health, role confidence, involvement in care, communication with staff, and social wellbeing. Infant outcomes emerged through parental descriptions of their infants' medical progress and well‐being. Staff outcomes primarily centred on parents' perceptions of staff competence, responsiveness, and collaboration with parents within FCC model.

Table 2 presents the derived outcomes and mapped according to corresponding domains. This table demonstrates how parents' lived experiences in NICUs can be translated into meaningful outcomes. However, we acknowledge that these outcomes are not yet clearly defined or measurable in standardised outcomes.

## 4 Discussion

The aim of this review was to synthesise evidence from qualitative studies exploring parents' experiences of FCC in NICUs. Subsequently, this review aimed to derive outcomes from these experiences to contribute to the development of a core outcome set for FCC. We aggregated four synthesised findings, reflecting main aspects of the parental journey. A wide range of parent, infant, and staff‐outcomes were derived from the synthesised findings, supported by direct quotations from the included studies. These outcomes extend beyond the results of our previous quantitative systematic review, which identified parental outcomes, mainly in the domain of emotional well‐being, most frequently reported outcomes stress, depression, and satisfaction [18]. Our qualitative systematic review shows that why these outcomes matter and how they were experienced by parents.

Although FCC has been widely implemented across various healthcare and community settings, including, disability services [78], paediatric intensive care units (PICU) [79] and adult intensive care units (ICU) [80], its operationalisation varies across settings. Qualitative systematic reviews conducted in PICU [79] and adult ICU [80] settings have similarly identified themes such as family involvement, communication, partnership with healthcare professionals, which are consistent with our findings. However, the vulnerability of preterm and critically ill infants, creates distinct relational and emotional dynamics in NICUs. Therefore, while our findings align with broader FCC literature, they must be interpreted within the specific clinical and organisational context of neonatal care.

The experience of having an infant admitted to the NICU profoundly alters the lives of parents. A recently published qualitative thematic synthesis aimed to identify barriers and facilitators to the delivery of FCC in NICUs based on parental experiences. It reported following main themes, parental mental health, staff‐parent partnership (power dynamics, staff attitudes consistency of care, and holistic support), informational needs, and NICU environment [81]. While there is some overlap with our findings, the aim of our systematic review is different. Our findings are consistent with existing evidence regarding the emotional toll of NICU admission on parental well‐being [82]. The most frequently reported outcomes based on parental' experiences were related to parental emotional and mental health. Supporting parental mental health is a fundamental requirement for improving parent's experience of FCC. Osborne et al. (2024) recommend enhancing healthcare professionals' training and competencies in mental health along with the implementation of supportive policies for parents [82]. In line with these recommendations, our finding emphasise the importance of preparing and informing parents about the NICU environment and infant's health to provide smooth adaptation for parents. Parental emotional well‐being is closely connected to infant's physical health outcomes, highlighting the importance of the interdependence of parent and infant health in NICU care.

Parents in our review described early feelings of inadequacy, disconnection, and role ambiguity, particularly among fathers, who often felt excluded and treated as secondary parents. However, caregiving involvement emerged as a transformative experience, fostering the development of parental autonomy, self‐efficacy, parental identity, role and bonding with the infant. These findings are consistent with previous research demonstrating how FCC fosters parental empowerment, parent‐infant relationship, and parents' involvement and taking responsibility in care [83]. Our review findings revealed several important parental outcomes that might help to assess parent‐reported experiences of FCC. As emphasised in a recent editorial by Latour et al., although FCC is a well‐established approach, its implementation remains inconsistent in clinical practice [84]. The authors identified a key barrier to implementation as the lack of validated tools and meaningful outcomes to assess parents' experiences of FCC [84]. Our review findings might support by translating FCC experiences into meaningfully and potentially measurable outcomes. However, this lack of standardisation and consistency in outcomes may contribute the continued challenges in implementing FCC in neonatal settings. Therefore, our findings demonstrate that the urgent need to develop a COS for FCC.

The relationship between healthcare professionals and parents was identified as an important determinant of parental experience. When healthcare professionals involved parents in rounds, listened to their input, and communicated transparently, parents felt empowered and informed. Conversely, inconsistent communication, and restricted access to information led to hesitancy, distress, and disempowerment. Our findings underscore that communication between healthcare professionals and parents is one of the key derivers of satisfaction. This finding aligns with a previous cross‐sectional study which found that the quality of interaction between healthcare professionals and parents significantly influences satisfaction and engagement [85]. In the USA, Lake et al. examined the relationship between parent satisfaction and missed nursing care in the NICU using the EMPATHIC‐N tool, which measures parental satisfaction and experiences. The authors identified that nursing responsibilities, such as counselling, teaching, supporting breastfeeding mothers, and preparing parents for discharge may contribute to parental satisfaction [86]. These responsibilities align with several outcomes derived from our review, particularly those related to parental self‐efficacy, perceived support from staff, and readiness for discharge. This highlights the importance of recognising outcomes related to healthcare professionals when evaluating FCC in NICUs.

To our knowledge, our review is the first systematic review identifying outcomes related to healthcare professionals based on parental experiences. These outcomes are typically overlooked when evaluating the impact of FCC interventions on infants and parents. A recently published qualitative systematic review exploring healthcare providers' perceptions of factors influencing implementation of FCC in NICUs reported inadequate staffing, lack of training and education for staff, and communication difficulties [87]. Another qualitative review found healthcare providers' attitudes and beliefs towards family‐integrated care significantly shaped the implementation [88]. Although healthcare professionals have an important role in the implementation of FCC, a recent scoping review found that there was only one randomised controlled trial that reported a healthcare professional, namely satisfaction [89]. Our review revealed several outcomes related to healthcare professional derived from parental experiences.

The findings of our review demonstrate that FCC must be evaluated using outcomes that extend beyond only infant's physical health. Currently, existing neonatal core outcome sets [90, 91] are not including parents and staff outcomes. Initially, Webbe et al., (2018) mapped the outcomes from parent, former neonatal patients, and clinicians' perspectives, however they identified a limited number of outcomes related to parents and staff, such as parental support, and healthcare workers knowledge and competence, and healthcare workers communication [92]. In contrast, our review systematically identified outcomes related to parents, infants, and staff, ensuring more holistic approach for the development of a COS for FCC.

To our knowledge, this is the first qualitative systematic review to derive FCC outcomes for parents, infants, and healthcare professionals based on parents' experiences. By using the JBI meta‐aggregative approach, we ensured a transparent and rigorous synthesis of evidence across a wide range of methodological design. A key strength of our review is the parent‐centred focus, with outcomes from parents' narratives, reflecting what truly matters to them in neonatal care. This makes the findings particularly valuable for informing the future development of COS for FCC.

However, some limitations must be acknowledged. The outcomes identified were not consistently defined or measurable across studies and remain largely conceptual, which limits their replicability and application in clinical research and practice. Like many qualitative systematic reviews, the inclusion of only qualitative studies may limit the generalisability of findings. However, the outcomes identified were clearly supported by parents' narratives across a diverse range of studies. Another limitation of this review is the exclusion of non‐English publications, which may have limited the inclusion of evidence from certain geographical and cultural contexts where FCC interventions are well‐established. Although the included studies represented a range of countries, relevant literature published in other languages may not have been captured, potentially influencing the cultural and contextual breadth of the findings. Nevertheless, the included studies covered multiple regions across Europe, North America, Asia, Africa, and Oceania, which partially mitigates this limitation. Finally, no stakeholder validation was conducted to confirm the relevance or prioritisation of the derived outcomes.

This review is the second part of a larger project to develop a COS for trials evaluating FCC interventions in NICUs. The project adheres to the guidance of the Core Outcome Measures in Effectiveness Trials (COMET) Initiative handbook, which recommends three sources for outcome identification: (1) systematic reviews, (2) interviews or focus group discussions, (3) national audit databases. We previously conducted a quantitative systematic review. As part of outcome identification process, this qualitative systematic review synthesises parents' experiences of FCC in NICUs to identify outcomes that matter to them. In the next phase, focus group discussions will be conducted with key stakeholders. Once a comprehensive list of outcomes has been generated from the systematic reviews and focus group discussions, an international three rounds e‐Delphi survey will be conducted to rank outcomes by stakeholders.

## 5 Conclusion

This qualitative systematic review highlights that parental experiences of FCC in neonatal intensive care settings are inherently relational, subjective, and shaped by contextual factors, including variations in FCC intervention design and implementation. The identification of both positive and challenging parental experiences reflects not only the subjective nature of parental experiences but also diversity in how FCC is conceptualised and operationalised across healthcare systems within and between countries. This conceptual and contextual variation may contribute to heterogeneity in reported outcomes.

The review identified a wide range of outcomes derived from parental experiences of FCC in NICUs. However, the outcomes reported across studies lacked consistency, standardised definitions, and measurability, which limit their comparability and application in FCC research and practice. These findings highlight the need to develop and implement a COS and validated outcome measures to benchmark best practices and guide future research, while acknowledging the conceptual and contextual diversity of FCC across settings.

## Author Contributions

**Cansel Kocakabak:** investigation, data curation, formal analysis, writing – original draft. **Agnes van den Hoogen:** conceptualization, methodology, investigation, data curation, formal analysis, writing – review and editing. **Melissa Rothfus:** methodology, data curation, writing – review and editing. **Marsha Campbell‐Yeo:** investigation, validation, writing – review and editing. **Aurelia Abenstein:** validation, writing – review and editing. **Anna Axelin:** validation, writing – review and editing. **Patricia Schofield:** validation, writing – review and editing. **Jos M. Latour:** conceptualization, methodology; investigation, data curation, formal analysis, validation, writing – review and editing. All authors final approval of the manuscript and accountability for all aspect of the work.

## Ethics Statement

Ethical approval was not required for this study as it is a systematic review of previously published literature.

## Conflicts of Interest

The authors declare no conflicts of interest.

## Supporting information

Supporting File 1

Supporting File 2

Supporting File 3

Supporting File 4

Supporting File 5

## Data Availability

The dataset generated and analysed during the current study are available from the corresponding author on reasonable request.
