# Supplementary material for: Parents' Experiences and Reported Outcomes of Family‐Centred Care: A Qualitative Systematic Review
Source: Health Expect. 2026 Apr 19;29(2):e70671. doi: 10.1111/hex.70671 (PMC13092509; doi:10.1111/hex.70671)
Supplement: Supplementary file 2 — Supporting File 2 [file HEX-29-e70671-s005.docx]

Search Databases: Medline, CINAHL, PsycINFO, Scopus, Embase, Cochrane Library, JBI, LILACS, Scielo

# Search Records

Medline (Ovid)

Date Searched: December 14, 2022

| **#** | **Query** | **Results**  (14/12/22) | **Results**  (20/11/23) |
| --- | --- | --- | --- |
| 1 | Family Nursing/ | 1,561 | 1,575 |
| 2 | Patient-Centered Care/ | 22,509 | 23,169 |
| 3 | professional-family relations/ or professional-patient relations/ or nurse-patient relations/ or Physician-Patient Relations/ | 150,834 | 151,589 |
| 4 | ((famil* or patient* or person or parent? or relationship) adj1 (cent?r* or focus* or integrat* or empower* or involv* or participa*)).ti,ab,kf | 122,262 | 133,786 |
| 5 | (family nursing or "fcc" or FIcare).ti,ab,kf. | 4,922 | 5,320 |
| 6 | 1 or 2 or 3 or 4 or 5 | 277,921 | 290,456 |
| 7 | Intensive Care Units, Neonatal/ | 17,669 | 18,378 |
| 8 | Intensive Care, Neonatal/ | 6,089 | 6,163 |
| 9 | (neonatal intensive care or "NICU").ti,ab,kf. | 29,486 | 31,897 |
| 10 | ((intensive care or special care unit or special care ward or critical care) adj4 (neonat* or newborn? or infant?)).ti,ab,kf. | 27,941 | 30,153 |
| 11 | 7 or 8 or 9 or 10 | 40,675 | 43,364 |
| 12 | 6 and 11 | 1,796 | 1,903 |
| 13 | limit 12 to yr="2022 -Current" |  | 260 |

CINAHL (EBSCO)

Date Searched: December 14, 2022

| **#** | **Query** | **Results**  (14/12/22) | **Results**  (20/11/23) |
| --- | --- | --- | --- |
| 1 | (MH "Family Nursing") | 2,214 | 2,142 |
| 2 | (MH "Patient Centered Care") | 34,978 | 35,524 |
| 3 | (MH "Professional-Patient Relations") OR (MH "Professional-Family Relations") OR (MH "Nurse-Patient Relations") OR (MH "Physician-Patient Relations") | 113,572 | 113,112 |
| 4 | TI ( ((famil* or patient* or person or parent# or relationship) N0 (cent#r* or focus* or integrat* or empower* or involv* or participa*)) ) OR AB ( ((famil* or patient* or person or parent# or relationship) N0 (cent#r* or focus* or integrat* or empower* or involv* or participa*)) ) | 72,016 | 75,549 |
| 5 | TI ( "family nursing" or "fcc" or FIcare ) OR AB ( "family nursing" or "fcc" or FIcare ) | 1,351 | 1,388 |
| 6 | S1 OR S2 OR S3 OR S4 OR S5 | 198,811 | 201,277 |
| 7 | (MH "Intensive Care Units, Neonatal") | 4,963 | 5,006 |
| 8 | (MH "Intensive Care, Neonatal") | 15,606 | 16,318 |
| 9 | TI ( "neonatal intensive care" or "NICU" ) OR AB ( "neonatal intensive care" or "NICU" ) | 16,618 | 17,352 |
| 10 | TI ( (("intensive care" or "special care unit" or "special care ward" or "critical care") N3(neonat* or newborn# or infant#)) ) OR AB ( (("intensive care" or "special care unit" or "special care ward" or "critical care") N3(neonat* or newborn# or infant#)) ) | 14,418 | 15,034 |
| 11 | S7 OR S8 OR S9 OR S10 | 26,405 | 27,294 |
| 12 | S6 AND S11 | 1,888 | 1,969 |
| 13 | Limiters - Publication Date: 20220101-20231231 |  | 253 |

PsycINFO (EBSCO)

Date searched: December 14, 2022

| **#** | **Query** | **Results**  (14/12/22) | **Results**  (20/11/23) |
| --- | --- | --- | --- |
| 1 | (DE "Patient Centered Care") OR (DE "Client Participation") | 3,499 | 4,004 |
| 2 | TI ( ((famil* or patient* or person or parent# or relationship) N0 (cent#r* or focus* or integrat* or empower* or involv* or participa*)) ) OR AB ( ((famil* or patient* or person or parent# or relationship) N0 (cent#r* or focus* or integrat* or empower* or involv* or participa*)) ) | 55,148 | 58,323 |
| 3 | TI ( "family nursing" or "fcc" or FIcare ) OR AB ( "family nursing" or "fcc" or FIcare ) | 578 | 612 |
| 4 | S1 OR S2 OR S3 | 57,777 | 61,207 |
| 5 | DE "Neonatal Intensive Care" | 1,890 | 2,040 |
| 6 | TI ( "neonatal intensive care" or "NICU" ) OR AB ( "neonatal intensive care" or "NICU" ) | 2,773 | 2,930 |
| 7 | TI ( (("intensive care" or "special care unit" or "special care ward" or "critical care") N3(neonat* or newborn# or infant#)) ) OR AB ( (("intensive care" or "special care unit" or "special care ward" or "critical care") N3(neonat* or newborn# or infant#)) ) | 2,518 | 2,661 |
| 8 | S5 OR S6 OR S7 | 3,478 | 3,658 |
| 9 | S4 AND S8 | 273 | 296 |
| 10 | Limiters - Published Date: 20220101-20231231 |  | 35 |

Scopus (Elsevier)

Date Searched: December 14, 2022

| **#** | **Query** | **Results**  (14/12/22) | **Results**  (20/11/23) |
| --- | --- | --- | --- |
| 1 | ( TITLE-ABS-KEY ( famil* OR patient* OR person OR parent? OR relationship ) W/0 ( cent?r* OR focus* OR integrat* OR empower* OR involv* OR participa* ) ) OR ( TITLE-ABS-KEY ( "family nursing" OR "fcc" OR ficare ) ) | 273,436 | 295,622 |
| 2 | TITLE-ABS-KEY ( ( "intensive care" OR "special care unit" OR "special care ward" OR "critical care" ) W/3 ( neonat* OR newborn? OR infant? ) ) | 46,625 | 51,220 |
| 3 | 1 and 2 | 1,358 | 1,516 |
| 4 | AND PUBYEAR > 2021 AND PUBYEAR < 2024 |  | 307 |

Embase (Elsevier)

Date searched: December 14, 2022

| **#** | **Query** | **Results**  (14/12/22) | **Results**  (20/11/23) |
| --- | --- | --- | --- |
| 1 | 'family centered care'/exp OR 'family nursing'/exp OR 'professional-patient relationship'/de OR 'doctor patient relationship'/de OR 'nurse patient relationship'/de | 173,397 | 175,375 |
| 2 | ((famil* OR patient* OR person OR parent$ OR relationship) NEAR/1 (cent$r* OR focus* OR integrat* OR empower* OR involv* OR participa*)):ti,ab,kw | 189,054 | 206,485 |
| 3 | 'family nursing':ti,ab,kw OR 'fcc':ti,ab,kw OR ficare:ti,ab,kw | 4,160 | 4,478 |
| 4 | #1 OR #2 OR #3 | 355,615 | 374,919 |
| 5 | 'neonatal intensive care unit'/exp OR 'newborn intensive care'/exp | 45,868 | 50,403 |
| 6 | 'neonatal intensive care':ti,ab,kw OR 'nicu':ti,ab,kw | 44,076 | 47,976 |
| 7 | (('intensive care' OR 'special care unit' OR 'special care ward' OR 'critical care') NEAR/4 (neonat* OR newborn$ OR infant$)):ti,ab,kw | 37,463 | 40,154 |
| 8 | #5 or #6 or #7 | 64,688 | 70,235 |
| 9 | #4 and #8 | 1,896 | 2,062 |
| 10 | #9 AND (2022:py OR 2023:py) |  | 347 |

Cochrane Library (all lines limited to Cochrane Reviews)

Date Searched: December 14, 2022

| **#** | **Query** | **Results**  (14/12/22) | **Results**  (20/11/23) |
| --- | --- | --- | --- |
| 1 | [mh "family centered care"] | 0 | 0 |
| 2 | [mh "family nursing"] | 0 | 0 |
| 3 | [mh ^"professional-family relations"] | 1 | 1 |
| 4 | [mh ^"professional-patient relations"] | 5 | 14 |
| 5 | [mh "nurse-patient relations"] OR [mh "physician-patient relations"] | 9 | 9 |
| 6 | (famil* or patient* or person or parent? or relationship NEAR/0 center* or centr* or focus* or integrat* or empower* or involv* or participa*):ti,ab,kw | 8467 | 8673 |
| 7 | ("family nursing" or "fcc" or FIcare):ti,ab,kw | 3 | 3 |
| 8 | {OR #1-#7} | 8467 | 8673 |
| 9 | [mh "Intensive Care Units, Neonatal"] | 25 | 28 |
| 10 | [mh "Intensive Care, Neonatal"] | 7 | 7 |
| 11 | ("neonatal intensive care" or "NICU"):ti,ab,kw | 151 | 162 |
| 12 | ((intensive care or special care unit or special care ward or critical care) NEAR/3 (neonat* or newborn? or infant?)):ti,ab,kw | 245 | 257 |
| 13 | {OR #9-#12} | 250 | 262 |
| 14 | #8 AND #13 | 239 | 250 |
| 15 | with Cochrane Library publication date from Jan 2022 to Nov 2023, in Cochrane Reviews |  | 28 |

JBI (Ovid)

Date Searched: December 14, 2022

| \| **#** \| **Query** \| **Results**  (14/12/22) \|  \|  \| \| --- \| --- \| --- \| --- \| --- \| \| 1 \| ((famil* or patient* or person or parent? or relationship) adj1 (cent?r* or focus* or integrat* or empower* or involv* or participa*)).ti,ab,kw. \| 132 \|  \|  \| \| 2 \| (family nursing or "fcc" or FIcare).ti,ab,kw. \| 1 \|  \|  \| \| 3 \| 1 or 2 \| 132 \|  \|  \| \| 4 \| ((intensive care or special care unit or special care ward or critical care) adj4 (neonat* or newborn? or infant?)).ti,ab,kw. \| 26 \|  \|  \| \| 5 \| 3 and 4 \| 5 \|  \|  \| |  |  |  |  |
| --- | --- | --- | --- | --- | --- | --- | --- | --- | --- | --- | --- | --- | --- | --- | --- | --- | --- | --- | --- | --- | --- | --- | --- | --- | --- | --- | --- | --- | --- | --- | --- | --- | --- | --- |
|  |  |  |  |  |
|  |  |  |  |  |
| LILACS (Virtual Health Library)  Searched: December 14, 2022 |  |  |  |  |
|  |  |  |  |  |
| **Query** | **Results**  (14/12/22) |  |  |  |
| ((mh:(patient-centered care)) OR (mh:(family nursing)) OR (mh:(professional-family relations)) OR (mh:(professional-patient relations)) OR (mh:(nurse-patient relations)) OR (mh:(physician-patient relations)) OR (("family centered" OR "family focused" OR "Patient centered" OR "patient focused" OR "parent focused" OR "patient integrated" OR "patient empowered" OR "family empowered"))) AND ((mh:(intensive care units, neonatal)) OR (mh:(intensive care, neonatal)) OR (("neonatal intensive care" OR "newborn intensive care"))) AND ( db:("LILACS")) | 39 |  |  |  |

Scielo (SciELO Network)

Searched: December 14, 2022

| **Query** | **Results** |  |
| --- | --- | --- |
| ("family centered" OR "family focused" or "Patient centered" or "patient focused" OR "Family Nursing") AND ("neonatal intensive care" OR "newborn intensive care") | 10 |  |

# Search Records

Medline (Ovid)

Date Searched: November 20, 2023

| **#** | **Query** | **Results** |
| --- | --- | --- |
| 1 | Family Nursing/ | 1,575 |
| 2 | Patient-Centered Care/ | 23,169 |
| 3 | professional-family relations/ or professional-patient relations/ or nurse-patient relations/ or Physician-Patient Relations/ | 151,589 |
| 4 | ((famil* or patient* or person or parent? or relationship) adj1 (cent?r* or focus* or integrat* or empower* or involv* or participa*)).ti,ab,kf. | 133,786 |
| 5 | (family nursing or "fcc" or FIcare).ti,ab,kf. | 5,320 |
| 6 | 1 or 2 or 3 or 4 or 5 | 290,456 |
| 7 | Intensive Care Units, Neonatal/ | 18,378 |
| 8 | Intensive Care, Neonatal/ | 6,163 |
| 9 | (neonatal intensive care or "NICU").ti,ab,kf. | 31,897 |
| 10 | ((intensive care or special care unit or special care ward or critical care) adj4 (neonat* or newborn? or infant?)).ti,ab,kf. | 30,153 |
| 11 | 7 or 8 or 9 or 10 | 43,364 |
| 12 | 6 and 11 | 1,903 |
| 13 | limit 12 to yr="2022 -Current" | 260 |

CINAHL (EBSCO)

Date Searched: November 20, 2023

| **#** | **Query** | **Results** |
| --- | --- | --- |
| 1 | (MH "Family Nursing") | 2,142 |
| 2 | (MH "Patient Centered Care") | 35,524 |
| 3 | (MH "Professional-Patient Relations") OR (MH "Professional-Family Relations") OR (MH "Nurse-Patient Relations") OR (MH "Physician-Patient Relations") | 113,112 |
| 4 | TI ( ((famil* or patient* or person or parent# or relationship) N0 (cent#r* or focus* or integrat* or empower* or involv* or participa*)) ) OR AB ( ((famil* or patient* or person or parent# or relationship) N0 (cent#r* or focus* or integrat* or empower* or involv* or participa*)) ) | 75,549 |
| 5 | TI ( "family nursing" or "fcc" or FIcare ) OR AB ( "family nursing" or "fcc" or FIcare ) | 1,388 |
| 6 | S1 OR S2 OR S3 OR S4 OR S5 | 201,277 |
| 7 | (MH "Intensive Care Units, Neonatal") | 5,006 |
| 8 | (MH "Intensive Care, Neonatal") | 16,318 |
| 9 | TI ( "neonatal intensive care" or "NICU" ) OR AB ( "neonatal intensive care" or "NICU" ) | 17,352 |
| 10 | TI ( (("intensive care" or "special care unit" or "special care ward" or "critical care") N3(neonat* or newborn# or infant#)) ) OR AB ( (("intensive care" or "special care unit" or "special care ward" or "critical care") N3(neonat* or newborn# or infant#)) ) | 15,034 |
| 11 | S7 OR S8 OR S9 OR S10 | 27,294 |
| 12 | S6 AND S11 | 1,969 |
| 13 | Limiters - Publication Date: 20220101-20231231 | 253 |

PsycINFO (EBSCO)

Date searched: November 20, 2023

| **#** | **Query** | **Results** |
| --- | --- | --- |
| 1 | (DE "Patient Centered Care") OR (DE "Client Participation") | 4,004 |
| 2 | TI ( ((famil* or patient* or person or parent# or relationship) N0 (cent#r* or focus* or integrat* or empower* or involv* or participa*)) ) OR AB ( ((famil* or patient* or person or parent# or relationship) N0 (cent#r* or focus* or integrat* or empower* or involv* or participa*)) ) | 58,323 |
| 3 | TI ( "family nursing" or "fcc" or FIcare ) OR AB ( "family nursing" or "fcc" or FIcare ) | 612 |
| 4 | S1 OR S2 OR S3 | 61,207 |
| 5 | DE "Neonatal Intensive Care" | 2,040 |
| 6 | TI ( "neonatal intensive care" or "NICU" ) OR AB ( "neonatal intensive care" or "NICU" ) | 2,930 |
| 7 | TI ( (("intensive care" or "special care unit" or "special care ward" or "critical care") N3(neonat* or newborn# or infant#)) ) OR AB ( (("intensive care" or "special care unit" or "special care ward" or "critical care") N3(neonat* or newborn# or infant#)) ) | 2,661 |
| 8 | S5 OR S6 OR S7 | 3,658 |
| 9 | S4 AND S8 | 296 |
| 10 | Limiters - Published Date: 20220101-20231231 | 35 |

Scopus (Elsevier)

Date Searched: November 20, 2023

| **#** | **Query** | **Results** |
| --- | --- | --- |
| 1 | ( TITLE-ABS-KEY ( famil* OR patient* OR person OR parent? OR relationship ) W/0 ( cent?r* OR focus* OR integrat* OR empower* OR involv* OR participa* ) ) OR ( TITLE-ABS-KEY ( "family nursing" OR "fcc" OR ficare ) ) | 295,622 |
| 2 | TITLE-ABS-KEY ( ( "intensive care" OR "special care unit" OR "special care ward" OR "critical care" ) W/3 ( neonat* OR newborn? OR infant? ) ) | 51,220 |
| 3 | 1 and 2 | 1,516 |
| 4 | AND PUBYEAR > 2021 AND PUBYEAR < 2024 | 307 |

Embase (Elsevier)

Date searched: November 20, 2023

| **#** | **Query** | **Results** |
| --- | --- | --- |
| 1 | 'family centered care'/exp OR 'family nursing'/exp OR 'professional-patient relationship'/de OR 'doctor patient relationship'/de OR 'nurse patient relationship'/de | 175,375 |
| 2 | ((famil* OR patient* OR person OR parent$ OR relationship) NEAR/1 (cent$r* OR focus* OR integrat* OR empower* OR involv* OR participa*)):ti,ab,kw | 206,485 |
| 3 | 'family nursing':ti,ab,kw OR 'fcc':ti,ab,kw OR ficare:ti,ab,kw | 4,478 |
| 4 | #1 OR #2 OR #3 | 374,919 |
| 5 | 'neonatal intensive care unit'/exp OR 'newborn intensive care'/exp | 50,403 |
| 6 | 'neonatal intensive care':ti,ab,kw OR 'nicu':ti,ab,kw | 47,976 |
| 7 | (('intensive care' OR 'special care unit' OR 'special care ward' OR 'critical care') NEAR/4 (neonat* OR newborn$ OR infant$)):ti,ab,kw | 40,154 |
| 8 | #5 or #6 or #7 | 70,235 |
| 9 | #4 and #8 | 2,062 |
| 10 | #9 AND (2022:py OR 2023:py) | 347 |

Cochrane Library (all lines limited to Cochrane Reviews)

Date Searched: November 20, 2023

| **#** | **Query** | **Results** |
| --- | --- | --- |
| 1 | [mh "family centered care"] | 0 |
| 2 | [mh "family nursing"] | 0 |
| 3 | [mh ^"professional-family relations"] | 1 |
| 4 | [mh ^"professional-patient relations"] | 14 |
| 5 | [mh "nurse-patient relations"] OR [mh "physician-patient relations"] | 9 |
| 6 | (famil* or patient* or person or parent? or relationship NEAR/0 center* or centr* or focus* or integrat* or empower* or involv* or participa*):ti,ab,kw | 8673 |
| 7 | ("family nursing" or "fcc" or FIcare):ti,ab,kw | 3 |
| 8 | {OR #1-#7} | 8673 |
| 9 | [mh "Intensive Care Units, Neonatal"] | 28 |
| 10 | [mh "Intensive Care, Neonatal"] | 7 |
| 11 | ("neonatal intensive care" or "NICU"):ti,ab,kw | 162 |
| 12 | ((intensive care or special care unit or special care ward or critical care) NEAR/3 (neonat* or newborn? or infant?)):ti,ab,kw | 257 |
| 13 | {OR #9-#12} | 262 |
| 14 | #8 AND #13 | 250 |
| 15 | with Cochrane Library publication date from Jan 2022 to Nov 2023, in Cochrane Reviews | 28 |

JBI (Ovid)

Date Searched: November 20, 2023

| \| **#** \| **Query** \| **Results** \| \| --- \| --- \| --- \| \| 1 \| ((famil* or patient* or person or parent? or relationship) adj1 (cent?r* or focus* or integrat* or empower* or involv* or participa*)).ti,ab,kw. \| 45 \| \| 2 \| (family nursing or "fcc" or FIcare).ti,ab,kw. \| 0 \| \| 3 \| 1 or 2 \| 45 \| \| 4 \| ((intensive care or special care unit or special care ward or critical care) adj4 (neonat* or newborn? or infant?)).ti,ab,kw. \| 11 \| \| 5 \| 3 and 4 \| 1 \| |  |  |
| --- | --- | --- | --- | --- | --- | --- | --- | --- | --- | --- | --- | --- | --- | --- | --- | --- | --- | --- | --- | --- |
|  |  |  |
|  |  |  |
| LILACS (Virtual Health Library)  Searched: November 20, 2023 |  |  |
|  |  |  |
| **Query** | **Results** |  |
| ((mh:(patient-centered care)) OR (mh:(family nursing)) OR (mh:(professional-family relations)) OR (mh:(professional-patient relations)) OR (mh:(nurse-patient relations)) OR (mh:(physician-patient relations)) OR (("family centered" OR "family focused" OR "Patient centered" OR "patient focused" OR "parent focused" OR "patient integrated" OR "patient empowered" OR "family empowered"))) AND ((mh:(intensive care units, neonatal)) OR (mh:(intensive care, neonatal)) OR (("neonatal intensive care" OR "newborn intensive care"))) AND ( db:("LILACS")) | 41 |  |

Scielo (SciELO Network)

Searched: November 20, 2023

| **Query** | **Results** |  |
| --- | --- | --- |
| ("family centered" OR "family focused" or "Patient centered" or "patient focused" OR "Family Nursing") AND ("neonatal intensive care" OR "newborn intensive care") | 11 |  |

# Search Records

Medline (Ovid)

Date Searched: May 30, 2025

| **#** | **Query** | **Results** |
| --- | --- | --- |
| 1 | exp Empirical Research/ or Interviews as Topic/ or Personal Narratives as Topic/ or Focus Groups/ or exp Narration/ or Nursing Methodology Research/ or Narrative Medicine/ | 207,600 |
| 2 | (Interview or Personal Narrative).pt. | 38,228 |
| 3 | interview*.ti,ab,kf. | 519,778 |
| 4 | qualitative.ti,ab,kf,jw. | 394,576 |
| 5 | (theme* or thematic).ti,ab,kf. | 214,358 |
| 6 | ethnological research.ti,ab,kf. | 8 |
| 7 | ethnograph*.ti,ab,kf. | 15,829 |
| 8 | ethnomedicine.ti,ab,kf. | 1,205 |
| 9 | ethnonursing.ti,ab,kf. | 140 |
| 10 | phenomenol*.ti,ab,kf. | 39,424 |
| 11 | (grounded adj (theor* or study or studies or research or analys?s)).ti,ab,kf. | 17,470 |
| 12 | life stor*.ti,ab,kf. | 1,791 |
| 13 | (emic or etic or hermeneutic* or heuristic* or semiotic*).ti,ab,kf. | 25,074 |
| 14 | (data adj1 saturat$).ti,ab,kf. | 2,791 |
| 15 | participant observ*.ti,ab,kf. | 6,102 |
| 16 | (social construct* or postmodern* or post-structural* or post structural* or poststructural* or post modern* or post-modern*).ti,ab,kf. | 4,849 |
| 17 | (action research or cooperative inquir* or co operative inquir* or co-operative inquir*).ti,ab,kf. | 6,665 |
| 18 | (humanistic or existential or experiential or paradigm*).ti,ab,kf. | 221,736 |
| 19 | (field adj (study or studies or research or work)).ti,ab,kf. | 23,460 |
| 20 | (human science or social science).ti,ab,kf. | 7,962 |
| 21 | biographical method.ti,ab,kf. | 27 |
| 22 | theoretical sampl*.ti,ab,kf. | 1,067 |
| 23 | ((purpos* adj4 sampl*) or (focus adj group*)).ti,ab,kf. | 102,646 |
| 24 | (open-ended or narrative* or textual or texts or semi-structured).ti,ab,kf. | 238,261 |
| 25 | (life world* or life-world* or conversation analys?s or personal experience* or theoretical saturation).ti,ab,kf. | 19,984 |
| 26 | ((lived or life) adj experience*).ti,ab,kf. | 25,997 |
| 27 | cluster sampl*.ti,ab,kf. | 10,885 |
| 28 | observational method*.ti,ab,kf. | 1,117 |
| 29 | content analysis.ti,ab,kf. | 53,556 |
| 30 | (constant adj (comparative or comparison)).ti,ab,kf. | 6,701 |
| 31 | ((discourse* or discurs*) adj3 analys?s).ti,ab,kf. | 3,845 |
| 32 | (heidegger* or colaizzi* or spiegelberg* or merleau* or husserl* or foucault* or ricoeur or glaser*).ti,ab,kf. | 5,639 |
| 33 | (van adj manen*).ti,ab,kf. | 634 |
| 34 | (van adj kaam*).ti,ab,kf. | 49 |
| 35 | (corbin* adj2 strauss*).ti,ab,kf. | 519 |
| 36* | or/1-35 | 1,363,076 |
| 37 | Family Nursing/ | 1,609 |
| 38 | Patient-Centered Care/ | 24,833 |
| 39 | professional-family relations/ or professional-patient relations/ or nurse-patient relations/ or Physician-Patient Relations/ | 154,585 |
| 40 | ((famil* or patient* or person or parent? or relationship) adj1 (cent?r* or focus* or integrat* or empower* or involv* or participa*)).ti,ab,kf. | 157,351 |
| 41 | (family nursing or "fcc" or FIcare).ti,ab,kf. | 5,983 |
| 42 | 37 or 38 or 39 or 40 or 41 | 317,000 |
| 43 | Intensive Care Units, Neonatal/ | 20,177 |
| 44 | Intensive Care, Neonatal/ | 6,250 |
| 45 | (neonatal intensive care or "NICU").ti,ab,kf. | 36,067 |
| 46 | ((intensive care or special care unit or special care ward or critical care) adj4 (neonat* or newborn? or infant?)).ti,ab,kf. | 33,909 |
| 47 | 43 or 44 or 45 or 46 | 47,977 |
| 48 | 42 and 47 | 2,142 |
| 49 | limit 48 to yr="2022 -Current" | 500 |
| 50 | 36 and 49 | 205 |

*Qualitative filter (lines 1-36) taken from: Qualitative Studies - MEDLINE. In: Canada's Drug Agency Search Filters Database. Ottawa: Canada's Drug Agency; 2025: <https://searchfilters.cda-amc.ca/link/40>. Accessed 2025-05-29.

CINAHL (EBSCO)

Date Searched: May 30, 2025

| **#** | **Query** | **Results** |
| --- | --- | --- |
| 1 | (MH "Family Nursing") | 2,259 |
| 2 | (MH "Patient Centered Care") | 40,023 |
| 3 | (MH "Professional-Patient Relations") OR (MH "Professional-Family Relations") OR (MH "Nurse-Patient Relations") OR (MH "Physician-Patient Relations") | 117,981 |
| 4 | TI ( ((famil* or patient* or person or parent# or relationship) N0 (cent#r* or focus* or integrat* or empower* or involv* or participa*)) ) OR AB ( ((famil* or patient* or person or parent# or relationship) N0 (cent#r* or focus* or integrat* or empower* or involv* or participa*)) ) | 80,551 |
| 5 | TI ( "family nursing" or "fcc" or FIcare ) OR AB ( "family nursing" or "fcc" or FIcare ) | 1,382 |
| 6 | S1 OR S2 OR S3 OR S4 OR S5 | 213,304 |
| 7 | (MH "Intensive Care Units, Neonatal") | 5,168 |
| 8 | (MH "Intensive Care, Neonatal") | 18,157 |
| 9 | TI ( "neonatal intensive care" or "NICU" ) OR AB ( "neonatal intensive care" or "NICU" ) | 18,612 |
| 10 | TI ( (("intensive care" or "special care unit" or "special care ward" or "critical care") N3(neonat* or newborn# or infant#)) ) OR AB ( (("intensive care" or "special care unit" or "special care ward" or "critical care") N3(neonat* or newborn# or infant#)) ) | 15,870 |
| 11 | S7 OR S8 OR S9 OR S10 | 29,457 |
| 12 | S6 AND S11 | 2,183 |
| 13 | Limiters - Publication Date: 20220101-20251231 | 493 |
| 14* | MH Qualitative Studies OR MH Grounded theory OR MH Narratives OR MH Interviews+ OR MH Audiorecording OR MH Focus Groups OR MH Research, Nursing OR MH Discourse Analysis OR MH Content Analysis OR MH Ethnographic Research OR MH Ethnonursing Research OR MH Constant Comparative Method OR MH Qualitative Validity+ OR MH Purposive Sample OR MH Observational Methods+ OR MH Field Studies OR MH Theoretical Sample OR MH Phenomenology OR MH Phenomenological Research OR MH Life Experiences+ OR MH Cluster Sample+ OR TI qualitative OR AB qualitative OR TI interview* OR AB interview* OR TI (theme* or thematic) OR AB (theme* or thematic) OR TI ("ethnological research") OR AB ("ethnological research") OR TI ethnonursing OR AB ethnonursing OR TI ethnograph* OR AB ethnograph* OR TI phenomenol* OR AB phenomenol* OR TI "focus group*" OR AB "focus group*" OR TI (grounded N1 (theor* OR analys?s OR research OR studies OR study)) OR AB (grounded N1 (theor* OR analys?s OR research OR studies OR study)) OR TI ("life stor*") OR AB ("life stor*") OR TI (emic OR etic OR hermeneutic* OR heuristic* OR semiotic) OR AB (emic OR etic OR hermeneutic* OR heuristic* OR semiotic) OR TI (data N1 saturat*) OR AB (data N1 saturat*) OR TI ("participant observ*") OR AB ("participant observ*") OR TI ("social construct*" OR postmodern* OR "post-structural*" OR poststructural* OR "post-modern*" OR feminis*) OR AB ("social construct*" OR postmodern* OR "post-structural*" OR poststructural* OR "post-modern*" OR feminis*) OR TI ("action research" OR "cooperative inquir*" OR "co-operative inquir*") OR AB ("action research" OR "cooperative inquir*" OR "co-operative inquir*") OR TI (humanistic OR existential OR experiential OR paradigm*) OR AB (humanistic OR existential OR experiential OR paradigm*) OR TI (field N1 (research OR study OR studies)) OR AB (field N1 (research OR study OR studies)) OR TI "human science" OR AB "human science" OR TI "biographical method" OR AB "biographical method" OR TI ("theoretical sampl*") OR AB ("theoretical sampl*") OR TI ("purpos* N4 sampl*") OR AB ("purpos* N4 sampl*") OR TI ("open-ended" OR narrative* OR textual OR texts OR "semi-structured") OR AB ("open-ended" OR narrative* OR textual OR texts OR "semi-structured") OR TI ("life world" OR "life-world" OR "conversation analys?s" OR "personal experience*" OR "theoretical saturation") OR AB ("life world" OR "life-world" OR "conversation analys?s" OR "personal experience*" OR "theoretical saturation") OR TI ((life OR lived) N1 experience*) OR AB ((life OR lived) N1 experience*) OR TI ("cluster sampl*") OR AB ("cluster sampl*") OR TI ("observational method*") OR AB ("observational method*") OR TI ("content analysis") OR AB ("content analysis") OR TI ((discurs* OR discourse*) N3 analys?s) OR AB ((discurs* OR discourse*) N3 analys?s) OR TI (constant N1 (comparison OR comparative)) OR AB (constant N1 (comparison OR comparative)) OR TI ("narrative analys?s") OR AB ("narrative analys?s") OR TI (heidegger* OR colaizzi* OR spiegelberg* OR merleau* OR husserl* OR foucault* OR ricoeur OR glaser*) OR AB (heidegger* OR colaizzi* OR spiegelberg* OR merleau* OR husserl* OR foucault* OR ricoeur OR glaser*) OR TI (van N1 manen*) OR AB (van N1 manen*) OR TI (van N1 kaam*) OR AB (van N1 kaam*) OR TI (Corbin* N2 strauss*) OR AB (Corbin* N2 strauss*) | 800,572 |
| 15 | 13 AND 14 | 216 |

*Qualitative filter (line 14) taken from: Qualitative Studies - CINAHL. In: Canada's Drug Agency Search Filters Database. Ottawa: Canada's Drug Agency; 2025: <https://searchfilters.cda-amc.ca/link/92>. Accessed 2025-05-29.

PsycINFO (EBSCO)

Date searched: May 30, 2025

| **#** | **Query** | **Results** |
| --- | --- | --- |
| 1 | (DE "Patient Centered Care") OR (DE "Client Participation") | 4,730 |
| 2 | TI ( ((famil* or patient* or person or parent# or relationship) N0 (cent#r* or focus* or integrat* or empower* or involv* or participa*)) ) OR AB ( ((famil* or patient* or person or parent# or relationship) N0 (cent#r* or focus* or integrat* or empower* or involv* or participa*)) ) | 63,872 |
| 3 | TI ( "family nursing" or "fcc" or FIcare ) OR AB ( "family nursing" or "fcc" or FIcare ) | 651 |
| 4 | S1 OR S2 OR S3 | 67,131 |
| 5 | DE "Neonatal Intensive Care" | 2,240 |
| 6 | TI ( "neonatal intensive care" or "NICU" ) OR AB ( "neonatal intensive care" or "NICU" ) | 3,150 |
| 7 | TI ( (("intensive care" or "special care unit" or "special care ward" or "critical care") N3(neonat* or newborn# or infant#)) ) OR AB ( (("intensive care" or "special care unit" or "special care ward" or "critical care") N3(neonat* or newborn# or infant#)) ) | 2,841 |
| 8 | S5 OR S6 OR S7 | 3,905 |
| 9 | S4 AND S8 | 321 |
| 10 | Limiters - Publication Date: 20220101-20251231 | 64 |

Scopus (Elsevier)

Date Searched: May 30, 2025

| **#** | **Query** | **Results** |
| --- | --- | --- |
| 1 | ( TITLE-ABS-KEY ( famil* OR patient* OR person OR parent? OR relationship ) W/0 ( cent?r* OR focus* OR integrat* OR empower* OR involv* OR participa* ) ) OR ( TITLE-ABS-KEY ( "family nursing" OR "fcc" OR ficare ) ) | 336,214 |
| 2 | TITLE-ABS-KEY ( ( "intensive care" OR "special care unit" OR "special care ward" OR "critical care" ) W/3 ( neonat* OR newborn? OR infant? ) ) | 58,435 |
| 3 | 1 and 2 | 1,817 |
| 4 | AND PUBYEAR > 2021 AND PUBYEAR < 2026 | 607 |
| 5* | TITLE-ABS-KEY ( "Empirical Research" OR interview OR "Interviews as Topic" OR "Personal Narratives" OR "Focus Groups" OR narration OR "Nursing Methodology Research" OR "Narrative Medicine" OR interview* OR qualitative OR theme* OR thematic OR "ethnological research" OR ethnograph* OR ethnomedicine OR ethnonursing OR phenomenol* OR "grounded theor*" OR "grounded study" OR "grounded studies" OR "grounded research" OR "grounded analysis" OR "grounded analyses" OR "life stor*" OR emic OR etic OR hermeneutic* OR heuristic* OR semiotic* OR "data saturat*" OR "participant observ*" OR "social construct*" OR postmodern* OR post-structural* OR "post structural*" OR poststructural* OR "post modern*" OR post-modern* OR feminis* OR "action research" OR "cooperative inquir*" OR "co operative inquir*" OR humanistic OR existential OR experiential OR paradigm* OR "field study" OR "field studies" OR "field research" OR "field work" OR "human science" OR "social science" OR "biographical method" OR "theoretical sampl*" OR ( purpos* W/3 sampl* ) OR ( focus W/3 group* ) OR open-ended OR narrative* OR textual OR texts OR "semi-structured" OR "life world*" OR "life-world*" OR "conversation analysis" OR "personal experience*" OR "theoretical saturation" OR "conversation analyses" OR "lived experience" OR "life experience*" OR "cluster sampl*" OR "observational method*" OR "content analysis" OR "constant comparative" OR "constant comparison" OR ( discourse* W/3 analysis ) OR ( discourse* W/3 analyses ) OR ( discurs* W/3 analysis ) OR ( discurs* W/3 analyses ) OR heidegger* OR colaizzi* OR spiegelberg* OR merleau* OR husserl* OR foucault* OR ricoeur OR glaser* OR "van manen*" OR "van kaam*" OR ( corbin* W/2 strauss* ) ) | 5,432,809 |
| 6 | 4 AND 5 | 238 |

*Qualitative filter (line 5) taken from: Qualitative Studies - Scopus. In: Canada's Drug Agency Search Filters Database. Ottawa: Canada's Drug Agency; 2025: [https://searchfilters.cda-amc.ca/link/94. Accessed 2025-05-29](https://searchfilters.cda-amc.ca/link/94.%20Accessed%202025-05-29).

Embase (Elsevier)

Date searched: May 30, 2025

| **#** | **Query** | **Results** |
| --- | --- | --- |
| 1 | 'family centered care'/exp OR 'family nursing'/exp OR 'professional-patient relationship'/de OR 'doctor patient relationship'/de OR 'nurse patient relationship'/de | 180,015 |
| 2 | ((famil* OR patient* OR person OR parent$ OR relationship) NEAR/1 (cent$r* OR focus* OR integrat* OR empower* OR involv* OR participa*)):ti,ab,kw | 246,207 |
| 3 | 'family nursing':ti,ab,kw OR 'fcc':ti,ab,kw OR ficare:ti,ab,kw | 5,088 |
| 4 | #1 OR #2 OR #3 | 418,693 |
| 5 | 'neonatal intensive care unit'/exp OR 'newborn intensive care'/exp | 60,046 |
| 6 | 'neonatal intensive care':ti,ab,kw OR 'nicu':ti,ab,kw | 55,931 |
| 7 | (('intensive care' OR 'special care unit' OR 'special care ward' OR 'critical care') NEAR/4 (neonat* OR newborn$ OR infant$)):ti,ab,kw | 46,264 |
| 8 | #5 or #6 or #7 | 81,120 |
| 9 | #4 and #8 | 2,446 |
| 10 | #9 AND [2022-2025]/py | 657 |
| 11 | 'qualitative research'/exp OR 'grounded theory'/de OR 'interview'/exp OR 'audio recording'/exp OR 'nursing research'/de OR 'ethnonursing research'/de OR 'discourse analysis'/de OR 'content analysis'/de OR 'ethnographic research'/de OR 'ethnography'/de OR 'constant comparative method'/de OR 'qualitative validity'/de OR 'purposive sample'/de OR 'observational method'/exp OR 'field study'/de OR 'theoretical sample'/de OR 'phenomenology'/de OR 'personal experience'/de OR 'narrative medicine'/de | 653,021 |
| 12 | 'empirical research':ab,kw,ti OR interview:ab,kw,ti OR 'interviews as topic':ab,kw,ti OR 'personal narratives':ab,kw,ti OR 'focus groups':ab,kw,ti OR narration:ab,kw,ti OR 'nursing methodology research':ab,kw,ti OR 'narrative medicine':ab,kw,ti OR interview*:ab,kw,ti OR qualitative:ab,kw,ti OR theme*:ab,kw,ti OR thematic:ab,kw,ti OR 'ethnological research':ab,kw,ti OR ethnograph*:ab,kw,ti OR ethnomedicine:ab,kw,ti OR ethnonursing:ab,kw,ti OR phenomenol*:ab,kw,ti OR 'grounded theor*':ab,kw,ti OR 'grounded study':ab,kw,ti OR 'grounded studies':ab,kw,ti OR 'grounded research':ab,kw,ti OR 'grounded analysis':ab,kw,ti OR 'grounded analyses':ab,kw,ti OR 'life stor*':ab,kw,ti OR emic:ab,kw,ti OR etic:ab,kw,ti OR hermeneutic*:ab,kw,ti OR heuristic*:ab,kw,ti OR semiotic*:ab,kw,ti OR 'data saturat*':ab,kw,ti OR 'participant observ*':ab,kw,ti OR 'social construct*':ab,kw,ti OR postmodern*:ab,kw,ti OR 'post structural*':ab,kw,ti OR poststructural*:ab,kw,ti OR 'post modern*':ab,kw,ti OR feminis*:ab,kw,ti OR 'action research':ab,kw,ti OR 'cooperative inquir*':ab,kw,ti OR 'co operative inquir*':ab,kw,ti OR humanistic:ab,kw,ti OR existential:ab,kw,ti OR experiential:ab,kw,ti OR paradigm*:ab,kw,ti OR 'field study':ab,kw,ti OR 'field studies':ab,kw,ti OR 'field research':ab,kw,ti OR 'field work':ab,kw,ti OR 'human science':ab,kw,ti OR 'social science':ab,kw,ti OR 'biographical method':ab,kw,ti OR 'theoretical sampl*':ab,kw,ti OR ((purpos* NEAR/3 sampl*):ab,kw,ti) OR ((focus NEAR/3 group*):ab,kw,ti) OR 'open ended':ab,kw,ti OR narrative*:ab,kw,ti OR textual:ab,kw,ti OR texts:ab,kw,ti OR 'semi-structured':ab,kw,ti OR 'life world*':ab,kw,ti OR 'life-world*':ab,kw,ti OR 'conversation analysis':ab,kw,ti OR 'personal experience*':ab,kw,ti OR 'theoretical saturation':ab,kw,ti OR 'conversation analyses':ab,kw,ti OR 'lived experience':ab,kw,ti OR 'life experience*':ab,kw,ti OR 'cluster sampl*':ab,kw,ti OR 'observational method*':ab,kw,ti OR 'content analysis':ab,kw,ti OR 'constant comparative':ab,kw,ti OR 'constant comparison':ab,kw,ti OR ((discourse* NEAR/3 analysis):ab,kw,ti) OR ((discourse* NEAR/3 analyses):ab,kw,ti) OR ((discurs* NEAR/3 analysis):ab,kw,ti) OR ((discurs* NEAR/3 analyses):ab,kw,ti) OR heidegger*:ab,kw,ti OR colaizzi*:ab,kw,ti OR spiegelberg*:ab,kw,ti OR merleau*:ab,kw,ti OR husserl*:ab,kw,ti OR foucault*:ab,kw,ti OR ricoeur:ab,kw,ti OR glaser*:ab,kw,ti OR 'van manen*':ab,kw,ti OR 'van kaam*':ab,kw,ti OR ((corbin* NEAR/2 strauss*):ab,kw,ti) | 1,650,964 |
| 13* | 11 OR 12 | 1,815,357 |
| 14 | 10 AND 13 | 239 |

* Qualitative filter (lines 11-13) adapted from: Qualitative Studies - CINAHL. In: Canada's Drug Agency Search Filters Database. Ottawa: Canada's Drug Agency; 2025: https://searchfilters.cda-amc.ca/link/92. Accessed 2025-05-29.

Cochrane Library (all lines limited to Cochrane Reviews)

Date Searched: May 30, 2025

| **#** | **Query** | **Results** |
| --- | --- | --- |
| 1 | [mh "family centered care"] | 0 |
| 2 | [mh "family nursing"] | 49 |
| 3 | [mh ^"professional-family relations"] | 1 |
| 4 | [mh ^"professional-patient relations"] | 13 |
| 5 | [mh "nurse-patient relations"] OR [mh "physician-patient relations"] | 8 |
| 6 | (famil* or patient* or person or parent? or relationship NEAR/0 center* or centr* or focus* or integrat* or empower* or involv* or participa*):ti,ab,kw | 8871 |
| 7 | ("family nursing" or "fcc" or FIcare):ti,ab,kw | 1 |
| 8 | {OR #1-#7} | 8871 |
| 9 | [mh "Intensive Care Units, Neonatal"] | 30 |
| 10 | [mh "Intensive Care, Neonatal"] | 7 |
| 11 | ("neonatal intensive care" or "NICU"):ti,ab,kw | 172 |
| 12 | ((intensive care or special care unit or special care ward or critical care) NEAR/3 (neonat* or newborn? or infant?)):ti,ab,kw | 265 |
| 13 | {OR #9-#12} | 270 |
| 14 | #8 AND #13 | 259 |
| 15 | with Cochrane Library publication date from Jan 2022 to May 2025, in Cochrane Reviews | 47 |

JBI (Ovid)

Date Searched: May 30, 2025

| \| **#** \| **Query** \| **Results** \| \| --- \| --- \| --- \| \| 1 \| ((famil* or patient* or person or parent? or relationship) adj1 (cent?r* or focus* or integrat* or empower* or involv* or participa*)).ti,ab,kw. \| 165 \| \| 2 \| (family nursing or "fcc" or FIcare).ti,ab,kw. \| 2 \| \| 3 \| 1 or 2 \| 165 \| \| 4 \| ((intensive care or special care unit or special care ward or critical care) adj4 (neonat* or newborn? or infant?)).ti,ab,kw. \| 29 \| \| 5 \| 3 and 4 \| 7 \| |  |  |
| --- | --- | --- | --- | --- | --- | --- | --- | --- | --- | --- | --- | --- | --- | --- | --- | --- | --- | --- | --- | --- |
|  |  |  |
|  |  |  |
| LILACS (Virtual Health Library)  Searched: May 30, 2025 |  |  |
|  |  |  |
| **Query** | **Results** |  |
| ((mh:(patient-centered care)) OR (mh:(family nursing)) OR (mh:(professional-family relations)) OR (mh:(professional-patient relations)) OR (mh:(nurse-patient relations)) OR (mh:(physician-patient relations)) OR (("family centered" OR "family focused" OR "Patient centered" OR "patient focused" OR "parent focused" OR "patient integrated" OR "patient empowered" OR "family empowered"))) AND ((mh:(intensive care units, neonatal)) OR (mh:(intensive care, neonatal)) OR (("neonatal intensive care" OR "newborn intensive care"))) AND ( db:("LILACS")) | 50 |  |
| Date range: 2022-2026 | 10 |  |

Scielo (SciELO Network)

Searched: May 30, 2025

| **Query** | **Results** |  |
| --- | --- | --- |
| ("family centered" OR "family focused" or "Patient centered" or "patient focused" OR "Family Nursing") AND ("neonatal intensive care" OR "newborn intensive care") | 11 |  |
