# Supplementary material for: Parents' Experiences and Reported Outcomes of Family‐Centred Care: A Qualitative Systematic Review
Source: Health Expect. 2026 Apr 19;29(2):e70671. doi: 10.1111/hex.70671 (PMC13092509; doi:10.1111/hex.70671)
Supplement: Supplementary file 3 — Supporting File 3 [file HEX-29-e70671-s001.docx]

**Electronic Supplemental Material 3: Characteristics of the studies included in the systematic review**

| **Author**  **Year**  **Country** | **Aim** | **Method** | **Population** | **Analysis** | **Main Findings** |
| --- | --- | --- | --- | --- | --- |
| Shafey et al., 2022  Canada | Experiences of fathers about FICare | Qualitative design; Interviews  Journal entries | Fathers (n=13) | Thematic Analysis | Fear of the unknown; Mental preparation; Identifying fathers ‘role; Parenting with supervision; Effective communication  Post neonatal care transition; Family life |
| Abukari et al., 2022  Ghana* | Experiences families and clinicians of FCC | Exploratory descriptive;  Interviews  Focus group | Family members (n=42)  Clinicians (n=42) | Content Analysis | Family experiences of FCC practices |
| Dien et al.,  2022  Canada | Experiences of mothers about FICare and Standard Care | Interpretive descriptive;  Interviews | Mother FICare (n=14)  Mothers Standard Care (n=12) | Constant comparative analysis | Recovering from birth; Adapting to the NICU; Caring for baby  Coping with daily disruption; Seeing progress; Supporting parenting |
| Ferreira et al., 2021  Canada | Parents experiences involvement in newborn’s care | Phenomenology;  Interviews  Focus group | Mothers (n=9)  Father (n=1) | Content analysis | Parent-staff interaction; Supportive/trustworthy healthcare professionals; Consistency in care and caring staff; Family couple and peer support; Newborn status; Resources and education for parents; NICU environment; Academic and research participation |
| van den Hoogen et al., 2020  Netherland | Experience of parents of an FCC programme | Qualitative design;  Interviews | Mother (n=11)  Fathers (n=2) | Thematic analysis | Involvement in care; Personalized information and communication; Transition to a parental role; Emotional support |
| Ndiaye et al., 2020 Africa | Experiences of parents about as primary careers in a low-income country | Qualitative descriptive;  Interviews | Mothers (n=10)  Fathers (n=2) | Thematic content analysis | General impressions of the neonatal unit; Communication;  Maternal stress |
| Neu et al., 2020  USA | Experiences of mothers about FCC | Qualitative descriptive;  Interviews | Mothers (n=14) | Thematic analysis | Visiting; General caregiving; Holding; Feeding; Maternal ideas for improvement |
| Lundqvist et al., 2019  Sweden | Experiences of parents of an FCC programme | Phenomenology; Interviews | Parents (n=6) | Phenomenological analysis | Feelings of existential loneliness and guilt; Challenges in becoming a mother; Ambivalent relationship to the partner; Professionals were supportive; Being unprepared for coming home; Neonatal home care-return to everyday life; Alignment to the needs of their partner; Stressed about managing everyday hassle; Father’s bonding process |
| Sarin et al., 2019  India * | Experience of parents about FCC | Qualitative design;  Interviews | Mothers (n=5)  Fathers (n=5)  Grandmother (n=1)  Grandfather (n=1) | Content analysis | Acceptability of FCC was based on gains in knowledge, access to the child, and improved well-being of the child; Interaction between staff and parent attendants was based on helpful communication; Empowerment and self-efficacy of parents |
| Maastrup et al., 2017  Denmark | Experience of parents about SSC | Qualitative descriptive;  Interviews | Mothers (n=9)  Fathers (n=2) | Thematic analysis | Overcoming ambivalence through professional support; Proximity and parental feeling; Feeling useful and realising the important of skin-to-skin contact; Bonding is beneficial regardless of survival. |
| Serlachius et al., 2018  New Zealand | Experiences of parents about FCC | Qualitative deductive;  Interviews | Mothers (n=63)  Fathers (n=20) | Thematic analysis | Disempowerment; Hierarchy between parents and staff; Father’s peripheral role |
| Broom et al., 2017  Australian* | Experiences of parents about FICare | Qualitative descriptive;  Focus group | Mothers (n=4)  Grandmother (n=1) | Content analysis | Benefits of FICare components; Enhancement of parent confidence and parental role attainment; Improved parent-parent communication |
| Treherne et al., 2017  Canada | Experiences of parents about closeness and separation | Qualitative descriptive;  Smartphone App | Mothers (n=13)  Fathers (n=7) | Content analysis | Having a role as a parent; Providing for and getting to know the infant; Support from staff; Reluctantly leaving the infant’s bedside; NICU environment |
| Brødsgaard et al., 2015  Denmark | Experiences of parents of an early discharge programme | Qualitative design;  Focus groups | Parents (n=9) | Content analysis | Respect and understanding of the family’s overall situation; A natural progression; Level of information before and during EDP; Participation and dialogue before and during EDP; Recommendation of EDP to others |
| Weis et al., 2013  Denmark | Experiences of parents about GFCC and SC | Descriptive comparative design; Interviews | Parents GFCC (n=13)  Parents SC (n=9) | Thematic analysis | Discovering and expressing emotions; Reaching a deeper level of communication; Obtain mutual understanding |
| Russel et al., 2014  UK | Experiences of parents about FCC | Qualitative design;  Interviews | Mothers (n=32)  Fathers (n=7) | Thematic analysis | Parental involvement; Staff competence and efficiency; Interpersonal relationship with staff |
| Finlayson et al., 2014  UK | Experiences of mothers about FCC | Qualitative design;  Interviews | Mothers (n=12) | Thematic analysis | Mothering in limbo; Deference to the experts; Anxious surveillance; Muted relations; Power struggles; Consistently inconsistent |
| Shirazi et al., 2016  Iran * | Experiences of caregivers about FCC | Hermeneutic phenomenology;  Interviews | Mothers (n=6)  Grandmother (n=1) | Van Manen’s analysis | Meta-family interaction; Comprehensive support; Reconstruction of a normal family |
| Lundqvist et al., 2007  Sweden | Experiences of fathers caring for infant | Hermeneutic phenomenology;  Interviews | Fathers (n=13) | Van Manen’s analysis | Feeling of distance; Feelings of proximity |
| Sarapat et al., 2017  Thailand | Experiences of parents about care involvement | Descriptive qualitative;  Interviews | Mothers (n=19)  Fathers (n=3)  Grandmothers (n=2) | Thematic analysis | Uncertainty about their child’s condition; Desire to be close their preterm babies; Lack of confidence in providing care; Overcoming difficulties in breastfeeding; Socio-cultural factors influencing parental involvement |
| Ingram et al., 2017  UK | Experiences of parents about FCC discharge program | Qualitative design;  Interviews | Parents (n=37) | Thematic analysis | Practical preparation: knowledge and skills transfer; Emotional preparation: uncertainty, feeling rushed, motivation to get home; Role of feeding: breastfeeding is the harder way to do it |
| Shirazi et al., 2018  Iran | Experiences of familial caregivers about FCC | Phenomenology;  Interviews | 6M  1F  1GM | Van Manen’s thematic analysis | Restoring stability; Oriented coalition; Dynamics of care;  Empowering the family |
| Mӧrelius et al.,2021  Sweden | Experiences of fathers about feeding | Qualitative inductive  Telephone interviews | 7 F | Content analysis | Shared responsibility for feeding process; A long and demanding process |
| Mohammadi et al.,2020  Iran | Experiences of mothers within context of dignity | Phenomenological;  Qualitative study | 20M | Colaizzi method | Privacy; Respecting individual identity; Authority |
| Patrikson et al., 2019  Sweden* | Describe communication between HCPs and parents in the presence of language barriers | Hermeneutic lifeworld;  Observation and interviews | 10 P | Phenomenological hermeneutic analysis following reflective lifeworld research | Wanting to speak for oneself; Being aware of cultural keys; Understanding one another in the employees ’arena |
| Axelin et al., 2018  Finland | Dynamics of neonatologist-parent communication and decision-making during medical rounds | Ethnographic approach;  Video recording and interview | 22 P  8M  7 Couple | Thematic analysis | Collaborative communication and decision-making; Neonatologist-led communication and decision-making; Emergency communication and decision-making; Disconnected communication and decision-making |
| Reid et al., 2010  Canada | Parents’ experience and satisfaction with care | Interpretive description;  In-depth interviews | 10 P | Thematic analysis | Perceptive engagement; Cautious guidance; Subtle presence |
| Heermann et al.,2005  USA | Experience of mothers | Qualitative design;  Interview | 15 M | Spradley’s domain analysis | Focus: from NICU to baby; ownership: from their baby to mu baby; caregiving: passive to active; voice: from silence to advocacy |
| Wigert et al., 2014  Sweden | Experiences of communication | Hermeneutic lifeworld; | 27 P  (16 M  11F) |  | Meeting a fellow human being; being included or excluded as a parent; bearing unwanted responsibility |
| Gotting et al., 2022  Sweden | Experiences of communication with hcps when language barrier | Qualitative study;  Interview | 17M  12 F  2 Family members | Content analysis | Communicating through pictorial support; Facing barriers in communication; Facing external influences; The need for a good healthcare relationship |
| Jafari et al.,2023  Iran* | Communication barriers related to parents for implementing FCC from parents’ perspective | Qualitative study;  Semi-structured interviews-field notes | 5M | Conventional content analysis | Mutually ineffective relationship between personnel and parents |
| Afeadie et al., 2023  Ghana | Experiences of mothers information and interaction with HCPs | In-depth interviews  Focus group discussions | 15 M | Thematic analysis | Socioeconomic status of participants; A healthcare workers’ mood at specific time; Physicians in possession of detailed information |
| Hajiaraghi et al.,2021  Iran* | Examine the strategic elements of FCC | Qualitative study;  Interview | 2F  3M | Conventional qualitative content | Family and care; Parental characteristics in care; Family needs |
| Dahan et al., 2023*  Canada | Experiences of families with information sharing | Qualitative study;  Interview | 10 P | Thematic analysis | Revolving Clinicians; Controlling the narrative; Impact on relationships; Impact on personhood |
| Sigurdson et al.,2020  USA | Experiences of families of FCC | Grounded theory;  In-depth interviews  Focus group | 18 Family members |  | Conflict with or lack of knowledge about social work; staff judgement of, or unwillingness to address barriers to family presence at bedside; need for nurse continuity and meaningful relationship with nurses and inconsistent access to translation services |
| Petersson et al., 2020  Sweden | Experiences of parents Family Health conversations | Interview | 12 family members | Content analysis | Co-creating a comprehensive picture; feeling validated; feeling equipped for the future |
| Park et al., 2024  South Korea* | Experiences of FCC among parents | Grounded theory;  Interview | 3P | Grounded theory | Wandering; Becoming a harmonious team |
| Shrestha et al.,  Nepal | Experiences of support and care | Descriptive phenomenological study | 25 P  (20M  5F) | Colazzi | Care and support; Initial involvement preterm infants’ care; Outcome of care involvement |
| Nukpezah et al.,2025  Ghana | Experiences of mothers following preterm birth delivery and hospitalization | Descriptive phenomenological study  Semi-structured interviews | 13M | Colazzi | Maternal anxiety about the unknown outcomes of the newborn condition; The positive impact of family-centred care; Maternal roles in preterm care; Poor support for maternal involvement in care |
| Guttman et al.,2024  USA | Parents perceptions of communication and understand parents communication needs and preferences | Semi structured interviews | 14  8M  6F | Thematic analysis | Strengths; Challenges; People; Coping Strategies |
| Eriksson et al.,2024  Sweden | Experiences of fathers about hospital based home neonatal care after discharge | Interview | 12F | Content analysis | Vivid memories from the NICU; Struggling with new challenges in life; Transition to home still in their thoughts |
| Stefana et al.,2024  Italy | Fathers perceptions and interactions with nurses | Ethnographic observation  Semi-structured interview | 20 F | Thematic analysis | Communication and clarity about infant’s health condition and progress; Inclusiveness and guidance from nurses; fathers’ satisfaction with nurses’ support for mother; nurses’ personal attention to the babies; nurse’s varied personalities |
| Cai et al.,2024  China | Experiences of parents of KC | Descriptive qualitative design  Interviews | 15 P | Thematic analysis | Low motivation upon initial engagement with KC; Dynamic fluctuations of emotional states during KC; Unexpected gains; Barriers to participation |
| Jafari et al., 2024  Iran* | Investigate parental barriers to implementing FCC | Semi-structured interviews  Field notes | 5 M | Conventional content analysis | Constraints parental role; Limitations parental involvement in care |
| Brodsgard et al.,2024  Denmark | Parents shared experiences of separation | Phenomenological hermeneutic design  Semi-structured interview | 8 P | Phenomenological hermeneutic approach | Becoming parents at different paces; Being at the Juncture between separation and Closeness |
| Osborne et al.,2024  USA | Identify opportunities for improvement by describing the experiences of parents | Semi-structured | 16 P | Content analysis | Medical team inclusion and connection; confusion regarding NICU care; discharge readiness; methods of communication |
| Schmid et al.,2024  Switzerland* | The views of parents on barriers and facilitators to parental presence | Semi-structured interviews  Focus group discussions | 20P  (10M  10F | Content analysis | Parent-professional interaction; physical and mental aspect; structural factors; organization and time management; resources; cultural aspect |
| Kabajassi et al.,2024  Uganda* | Identify facilitators and constraints that informed the adaptation of FICare | Interviews  Focus group | 10 M | Content analysis | The healthcare provider’s workload would be reduced by offloading tasks to mothers; Neonatal outcomes would be improved by involving mothers in patient care; Mothers would be empowered by assuming more responsibility; Maternal stress would increase with increased responsibility; mothers would not be able to learn new skills; Healthcare providers would not trust maternal assessments |
| Banazadeh et al.,2024  Iran* | Explore parental factors affecting parents’ participation in decision-making | Qualitative study Interviews | 10 P | Conventional Content analysis | Parental capabilities; A sense of parental self-efficacy; Conviction; living conditions |
| Franck et al.,2024  USA | Explore how a mobile app designed to support FICare influences parents experience | Interview | 9 M  1F | Thematic analysis | Actively caring for the infant; Learning how to care their infant; Learning about the clinical status of their infant |
| Ondusko et al.,2025  USA | To explore the needs and experiences of Black families and identify strategies to improve FCC and equity | Interview  Focus group | 13 Family  (10M  3F) | Reflexive Thematic analysis | Experiences; Recommendations |
| Ajayi et al., 2024  USA | Experiences of provider communication, support needs, and overall experience | Grounded theory | 12 M | Thematic analysis | Maternal care/nursing experiences; Interaction NICU; Support needs |

*Only data of family members were included in the review.

F=Father;M=Mother; FICare=Family Integrated Care; FCC=Family-Centred Care; NICU=Neonatal Intensive Care Units; FM=Family Members; KMC=Kangaroo Mother Care; SC=Standard Care.
