# Supplementary material for: Parents' Experiences and Reported Outcomes of Family‐Centred Care: A Qualitative Systematic Review
Source: Health Expect. 2026 Apr 19;29(2):e70671. doi: 10.1111/hex.70671 (PMC13092509; doi:10.1111/hex.70671)
Supplement: Supplementary file 4 — Supporting File 4 [file HEX-29-e70671-s002.docx]

**Table 1.** Synthesized Finding 1: The Impact of NICU Admission on Parental Mental Health

| **Table 1: Synthesized Finding 1: The Impact of NICU admission on Parental Mental Health** | | |
| --- | --- | --- |
| **Findings** | **Categories** | **Synthesized Finding** |
| *Fear of the unknown*: He was in there for so long, but still a lot of complications that we knew could happen and the concern was always I guess nagging about what could, or you know, could not happen. ^1^ | Parental fear and worry related to infant’s health and survival | This synthesized finding reflects the emotional and psychosocial experiences of parents during their infant’s admission to and hospitalization in the NICU. Parents described feelings of fear, worry, guilt, isolation, self-blame, and stress, which were triggered by factors such as uncertainty about their infant’s health and survival, separation from their infants, and environmental, and financial factors. While these factors contributed to psychological distress, supportive information and preparation related to premature birth, the NICU environment and available resources helped to alleviate the psychological impact on parents. |
| *Worrying about the child’s condition:* ...in the first few days, his condition was very terrible. It seemed like he was going to die…he was so tired and breathed so strongly…I was so worried that my child won’t survive…I cried all night. (ID, M9) ^2^ |  |  |
| *Emotional stress:* “You can’t sleep”, “you feel like your baby is crying”, and boxes are carried with dead babies”. ^3^ |  |  |
| *Fear of their baby dying: “*What worries me the most is to see some deaths around here. I know that maybe the next baby could be mine” ^4^ |  |  |
| *Living with worry:…* it was like, you rather not touch him because you thought then I'll rip off this or that tube or something like that. So, it was a bit, it was hard.^5^ |  |  |
| *Being afraid of their babies being at risk*: I didn’t want to do much because my baby was so small. I didn’t dare turn his body to one side. He’s very tiny and his bones may not be very strong…I was scared, particularly for the neck, as I heard that a newborn baby’s bones are still weak. (ID, M16)^2^ |  |  |
| *Conflicting feelings and needs: "*I was nervous that everything on her would stop, and they would come and tear her out of my arms and resuscitate her." ^6^ |  |  |
| *Uncertainty, feeling rushed, motivation to get home:* It came to the point where we, yeah, we were getting prepared to do it and, yeah, nervous, scared, but excited at the same time…mentally kind of preparing how we were going to do things, continuing it at home rather than having the hospital around to help.^7^ |  |  |
| *Attempting to produce enough milk supply:* I never stopped pumping my breasts even a single time because I’m worried that if my baby starts getting milk, I would have no breast milk for her… So I try to express milk every 2-3 hours to keep my milk… ^2^ |  |  |
| *Maternal anxiety about the unknown outcome of the newborn condition:* It is an emotional rollercoaster because some days you might feel there is improvement, but the next day you feel that there is a relapse. (M7) ^8^ |  |  |
| *Fear and worry about future medical problems for the neonate:* "When I saw my baby first time, I couldn't control myself or do anything at all, I was crying all the time, I thought when my baby was admitted to here, he might not be able to see, or hear, and his brain would have problems.” ^9^ |  |  |
| *Fear of unintentionally harming the neonate:* “Because of the transmission of infection, I preferred not to touch my baby, even though the staff said it was necessary.” ^10^ |  |  |
| *Concerns about the infant’s future*: ‘I definitely made sure that I enrolled him in, like, the – we call it first step here, and it just works on developmental issues.’^11^ |  |  |
| *Isolation and loneliness:* “Because of poverty, my husband cannot find the ticket (transportation fare) to come visit me every day. My father is too old to move around. My mother is no longer. ^4^ | Feelings of isolation and guilt due to separation from the infant |  |
| *Being unprepared for coming home:* "Coming home was an adjustment, she felt like the loneliest person in the world because she was the only one who could take care of the infant" (M52).^12^ |  |  |
| *Feelings of existential loneliness and guilt:* She felt guilty about the infant being born prematurely and experienced this as defeat (M4). ^12^ |  |  |
| *Reluctantly leaving the infant’s bedside:* “I feel a sense of abandonment and I have to leave earlier than planned so I am feeling a little guilty too.”^13^ |  |  |
| *Facing negative emotions:* “I have a lot of guilty conscience that my baby was born early. I cry all the time, and I can't get close to her because it was my fault; if I hadn't lifted the carpet, my water bag wouldn't have broken, and my baby wouldn't have been born early.” ^9^ |  |  |
| *Using the Phone for Communicating Proved to Be an Ambivalent Method:* “I think it was so difficult because you just see pictures . . . but you haven’t held them, and you barely know what they say when they cry” (M2)^14^ |  |  |
| *Technology is overwhelming:* Obviously if you stay there overnight, the monitors don’t turn off, they keep going every night. So, for me I think it was important to go home, get a little bit of a break to rest and recover myself, however, every time I left my emotions were very high because I felt as if I was leaving my baby. (ID 202 Standard Care) ^15^ | Stress related to parental vulnerability and external caregiving environment |  |
| *Concern about their own health:* What worries me the most is my own health. I have had surgery, and the after-surgery is still painful” ^4^ |  |  |
| *Conflict between caring for self and infant:* ‘So, he would be getting his antibiotics at 10:00, and I would have to get mine at 10:00. So, I wasn’t able to stay with him during that time which also meant missing out one time where to feed him and to change him and things like that that we could participate in. I wasn’t necessarily able to stay that long when I was able to visit him, because I had to go back to my room to get my own care done.’^11^ |  |  |
| *Financial worries: “*as a poor person my worry is how I will pay the medical expenses for my baby. (…) I know it is cheaper with mutuelle (health insurance) paying most of the costs, but it also depends on how long you stay” ^4^ |  |  |
| *Lack of information:* The absence of an open flow of information and feedback on the processes and progress of their children’s illness were unpleasant experiences according to the families. This hampered their ability to fully participate in ICU care and exacerbated their anxiety.^3^ |  |  |
| *Stressed about managing everyday hassle:* Felt that it took a lot of energy to keep up with everything at home, visit them in the NICU and make sure that the older children did not suffer (F8)^12^ |  |  |
| *Anxious surveillance:* When the red light is flashing and that alarms going off I’m having palpitations wondering why nobody was doing anything.^16^ |  |  |
| *Conflict between work/family affairs and KC:* “I have an older child to take care of at home, and there is no one to help with looking after him. My husband must work, while my mother-in-law, at most, helps with cooking because she is older. Basically, I must take care of the child at home on my own, so I can’t afford to spare any time.” (P8).^17^ |  |  |
| *Parents busy and not visiting the neonate:* “I have another child at home and there is no one to take care of him, so I can't come to be with my baby very often. ^10^ |  |  |
| *Organization and time management:* “Of course I would have liked to come, but our boy is still at home. I have been in the women’s hospital for so long, even before that. I have to be at home sometime. It is also for him.” (M05)^18^ |  |  |
| *Cultural aspects:* “She doesn’t say what’s on her mind and I think to myself, “Sometimes you just have to speak for yourself.” But she doesn’t dare. The confidence is not there.” (F09)^18^ |  |  |
| *Maternal stress would increase with increased responsibility:* ‘If something is abnormal with the baby, definitely my stress will increase’ ^19^ |  |  |
| *Caring for siblings:* “I have two other children at home. I have to do the housework and take care of them and check their homework” (M1).^20^ |  |  |
| *Balancing life:* ‘I am single, and I have two kids under the age of five now. So, I was parenting a newborn and a toddler at the time, so that was hard, and it still is hard. It’s a lot of work.’^11^ |  |  |
| *Mental preparation:* Quote 2A: It was around 28 weeks that my wife went for a regular check-up and they noticed very high blood pressure ... one good thing is that ... they kind of prepared us for potentially having a 28 week old baby. We did get an opportunity to tour around the NICU, which was really good, it definitely made the experience when we did go there at 32 weeks a lot less overwhelming just because we had seen it.^1^ | Supportive information and preparation to reduce the emotional toll of the NICU experience |  |
| *Resource and education for parents:* “There should be some sort of way to present to parents, this is the NICU, this is what you can expect kind of thing, like, just one information session. Parents should be invited to one information session.” (FG#3)^21^ |  |  |
| *Maternal ideas for improvement:* I have been here for 14 weeks, and I never knew this (parent lounge) was here! Maybe, like, after parents get settled in if they’re going to be here for the long road to be able to show them their resources and actually show them where they are. Because I didn’t know where case management was until I started to get the boys moved to our local hospital and I was calling everyone I could possibly think about. The social worker came by and said “Hi”, but because I wasn’t here all the time…it just would have been nice for someone to say, “Here are their offices if you ever need to talk to them. Here’s lactation offices, here’s the family lounge, help yourselves to whatever is in here,” just stuff like that.^22^ |  |  |
| *Be prepared:* “I believe we hadn’t thought much about that, but we thought she should have breastmilk, at least.”^23^11 |  |  |

**Table 2:** Synthesized Finding 2: Becoming a Parent through Caregiving Involvement

| **Table 2: Synthesized Finding 2: Becoming a Parent through Caregiving Involvement** | | |
| --- | --- | --- |
| **Findings** | **Categories** | **Synthesized Finding** |
| *Parenting with supervision:* **"**My nurses were just; they were all up front with everything that it was. If we needed something, we’d go get it. If you need this, you go get it ... It helped speed up. It, it was good ... We were basically parenting, with supervision. For, nine days, while we got ready to go home." (Interview 7)^1^ | Parental Autonomy and Self-efficacy in Caregiving | This synthesized finding explores how both active participation and restricted participation in caregiving, along with the structural conditions of the NICU environment and policies, shape and influence the development of parental characteristic and identity.  Parents who were actively involved in caregiving activities, which were feeding, holding, changing diaper, and making decisions, reported increased confidence, a stronger emotional bond with their infant, and growing sense of parental role. In contrast, parents who experienced restrictions on their participation due to institutional polices, physical discomfort, limited visiting hours, described feeling of inadequacy, emotional disconnection with their infant. These experiences shows that the involvement of parents their infant care and the development of parental identity is not only an emotional or personal journey, but also is deeply influenced by the structural environment and policies. |
| *Lacking control:* Not being allowed to read his files was stressful. It’s all about my baby. I wanted to know everything going on. It’s my baby and me. It puts you at ease knowing what’s happening’ (Mother, Discharge, #18) ^24^ |  |  |
| *Bedside education and participating rounds:* “I would stay to do care rather than go to an education session.”^25^ |  |  |
| *Feeling autonomous and making decisions:* For a brief moment you feel like you are in control again, which for me gives me the sense of responsibility and bonds me to my baby.^13^ |  |  |
| *Empowerment and self-efficacy of parents:* R: I will do at home what I am doing here.  I: But at home you have many people, and it is not that everyone will come with their hands washed, right?  R: I will tell them to wash, I will tell them to do like this, otherwise he will be sick again… After all this effort, we take him out of the hospital and he will again go to the hospital, so don’t do that. So, I will not allow them to touch [him] without washing [their] hands. (M03).^26^ |  |  |
| *A natural progression:* Although initially it was scary, I developed the skills, it was a natural progression; we learned how to take care of him.^27^ |  |  |
| *Acceptability of FCC among family members was based on gains in knowledge, access to the child, and improved well-being of the child:* This is good because if we cannot see meet our children, then how would attachment develop and how would our child know us and feel us? How would they know that we are their parents? It is the good part that we are able to stay here and can take care of our babies. We can see what doctors are doing and what is the temperature of child. We are being informed about machines as well now.” (M03).^26^ |  |  |
| *The level of information before and during the Early discharge program matched the needs of the families:* There was so much information, to take in before we could... know whether it [EDP] suited us. There was plenty of time to consider it really thoroughly and ask questions.^14^ |  |  |
| *Looking after their own:* “[The neonatal unit] are great about you being able to open the incubator and they get you very involved in looking after them, and changing nappies, and cleaning bottoms and so you’re doing as much of the care as you can”. (12, mother, D/C).^28^ |  |  |
| *Performing care for their babies with sufficient skills:* I never changed a baby’s diaper. I was quite bad at it…When I changed a diaper for her, I didn’t know if she’ll get hurt…I have to try it anyway. As she doesn’t stay still…Now I’m better at changing my baby’s diaper. (ID, M19)^2^ |  |  |
| *Believing in the benefits of breast milk:* I want to breastfeed my baby...Breast milk is good, it’s better than everything else…It makes my baby healthier as it has more antibodies than formula milk. (ID, M4)^2^ |  |  |
| *Breastfeeding is the harder way to do it”:* Everybody acknowledged that breastfeeding is the harder way of doing it. And a lot of babies that were bottle-fed left sooner than us. (Mother #305)^7^ |  |  |
| *Share decisions:* “Actually, the biggest problem was when we decided that we should quit pumping, so to say…” ^23^ |  |  |
| *A long process:* “At the moment, yesterday and today, we try, for an intensive period, to breastfeed again.” ^23^ |  |  |
| *Self-control of life:* Entering and getting out the unit are scheduled in specific hours. You can’t enter the unit in any time. True, this is so to control infection and in the end is for my baby’s good but I’m not in a good physical and psychological condition as well, sometimes, I feel like going out of unit and go to my family. But here, you have no options. You can’t do anything as you want. I feel very weak and incapable . . . . (P4)^29^ |  |  |
| Voice: From silence to advocacy: “I said something to one of the nurses about how the baby likes to be positioned. She really likes being laid a certain way and her legs tucked in a certain way. The next day the nurse said, ‘We have to write this down because this baby was upset and mad and not going to sleep and I tucked her in just the way you described, and she was out like a light.’ That was really the first time I really felt like I knew more about this baby than all these other people.”^30^ |  |  |
| *Bearing unwanted responsibility:* Vague communication, should we remember when the child needs feeding or should the staff do it, and sometimes in case we forgot it was as if we were supposed to take on the responsibility.^31^ |  |  |
| *Having expectations:* “Sometimes it feels like if you don’t insist, they can be a bit ignorant or not take things seriously. What I mean is that if you can communicate and stand up for yourself, they will listen better to you, and of course the language matters in this matter too.” (P12)^32^ |  |  |
| *United care with concerns:* “It doesn’t matter if the family participates in basic activities, such as feeding or changing diapers, but I think it would be a disadvantage if other medical problems arise.” (P2)^33^ |  |  |
| *Positive impact of FCC*: It gives me reassurance and confidence being involved in the process. (M1)^8^ |  |  |
| *Eager to participate yet concerned about not being competent:* When I saw those tiny hands and feet that day, I felt a bit worried. I was concerned that I might use too much force or make too sudden movements, afraid that I wouldn’t do it well.” (P12)^17^ |  |  |
| *The healthcare provider's workload would be reduced by offloading tasks to mothers:* When I learn, I will not disturb the doctors as much.’ ^19^ |  |  |
| *Neonatal outcomes would be improved by involving mothers in patient care:* This ‘program will reduce the death of children.^19^ |  |  |
| *Mothers would be empowered by assuming more responsibility:* You start being confident and know that whatever you are measuring is right.’^19^ |  |  |
| *Awareness of self‑abilities*: “I am not breastfeeding. No need I stay with her” (M7).^20^ |  |  |
| *Learning about the clinical status of their infant and partnering in care planning* “I felt so prepared by the nurses and by all the different healthcare providers to be able to feel like I can manage this, you know, I know all the steps and all the parts of their care.”^34^ |  |  |
| *Support the parenting role*: More communication, more teaching skills. Introducing parents to how to hold the baby, how to feed a baby,how to do skin-to-skin, where is a safe place to lay a child…^35^ |  |  |
| *Persistently questioning providers*: ‘I have a lot of basic knowledge of what was going on, so I understood what was going on, and I understood what questions to ask. So I had a really good picture of what was going on with the kids, and my questions were answered after we had a conversation, and I actually did have to bring up, I felt it was necessary to tell them, ‘I have a biology degree. So when I asked you a question, I need an actual answer.’ ^11^ |  |  |
| *Devising strategies to hold providers accountable:* ‘I felt like they weren’t really listening to me again. And so, what I ended up doing was I called her [notable Black obgyn] on my cell phone and I put her on speakerphone … And so, as soon as they [care team] found out that I was one of her patients, it really switched. They started treating me differently and everything.*’*^11^ |  |  |
| *Socioeconomic status of participants:* I know it is their responsibility to tell me the status of my baby anytime I come to visit. They are here because of us, so I ask a lot of questions, whether the questions are irrelevant or not. It is my baby, so I have the right to know everything. [Adzo 34 years, immigration officer IDI].^36^ |  |  |
| *The need of family information:* I told the doctor I would like to know about any problem with my child I am a mother; I have the right to know it.^37^ |  |  |
| *Controlling the narrative:“*feel like I would have control over the situation a little bit more”^38^ |  |  |
| *Newborn status and care:* Skin to-skin contact is “[. . . ] very important. It just encourages the mother to attach.” (FG#2)^21^ | Parent-Infant Bonding through Caregiving |  |
| *The fathers’ bonding process was vaguely expressed:* He had not had much time with the infant, and therefore ended up thinking “now what”? (F4)^12^ |  |  |
| *Providing for and getting to know the infant feeding, holding, and interacting:* “That’s how we discover who our little baby is.”^13^ |  |  |
| *Holding:* We did skin to skin the night they were born. They were on CPAP. I do skin-to-skin with both of them together now every day.^22^ |  |  |
| *Ownership and proximity to baby:* We have to go home every day and come in twice a day … . Our presence with our baby. We want to be close. For nearly everything’ (Father, Admission, #47)^24^ |  |  |
| *An important difference for the infant:* He does not need to constantly be soothed [as he does when he is in the incubator]’ [P5]^6^ |  |  |
| *Bonding with their babies through breastfeeding:* I’m so happy and glad. When looking at my baby’s face, I think: “Wow the feeling is like this.” I don’t know how to explain. When I look at his face, while his eyes are closed, his mouth is sucking milk from my breasts. My baby is so cute…I love him so much. (ID, M3)^2^ |  |  |
| *Gradual enhancement of infant-parents attachment:* “Later on, after starting feeding, I was called there two-hourly for feeding. At that time, we used to touch, hold, interact with him, feed breast milk and change his diaper.” (M18)^39^ |  |  |
| *Promotion of infant-parent attachment*: “During breastfeeding, I felt closer to her like having a heart-to-heart connection. Lots of mothering love flows towards my baby.” (M14)^39^ |  |  |
| *Skin-to-skin contact:* I think every mother will love it a lot for that one. I enjoy it so much, making me love my baby more. (M4)^8^ |  |  |
| *Vivid memories from the time at the NICU:* Although you knew there was a child, it doesn’t become real because you don’t. . . feel it (. . .) It doesn’t become real. . . it doesn’t become real until you really get to hold it. . . and have it.^40^ |  |  |
| Having Shared Experiences Was Essential for the Feeling of Being a Family: I’m happy that there were . . . some experiences that I was part of for the first time. When they were in the bath . . . so it wasn’t just another thing that you [father’s name] had tried. It was the first time for both of us; that was nice. (M2)^27^ |  |  |
| *The mothers as the core element of care:* I would go to the NICU and say, “well, let me do something, and I will help you as much as I can (M)’^37^ |  |  |
| *Challenges in becoming a mother:* In the beginning, well, what was she even doing there? (M32)^12^ | Developing Parental Identity and Role through Involvement |  |
| *Mothering in limbo:* Today is the first time I’ve been on my own with her and it’s bad in a way because it should be really nice but it’s not really. Although it’s great that I’m on my own with her, it’s just in this hospital room. . . . it just feels like pretend still . . . like I pretended to have a baby . . . that’s what it feels like.^16^ |  |  |
| *Longing to perform a maternal role:* …I could do everything for my baby…because she is my child...I’m her mother…so I have to raise and look after her. (ID, M14)^2^ |  |  |
| *Identifying fathers’ roles:* When you introduce a third member to your family ... like the dad’s number 3 all of a sudden so. That’s going to be in the book I write on being a father its called, “You’re number 3” ... Moms were treated kind of as the primary and um you know it was the way they kind of they didn’t really speak to you as a family they speak to mom type of deal in that sense. (Interview 3, standard care)^1^ |  |  |
| *Becoming an outsider:* … of course, they're much better at it than me, well, not better, I just mean it goes faster when they change (11, p. 5) ^5^ |  |  |
| *Transition to a parental role:* "We did the care all by ourselves. It was our own process and very meaningful for us to feel complete as a parent" (M5).^41^ |  |  |
| *Role of parent:* ‘You’re going through so much, and you don’t feel like it’s your baby unless you do the cares. It gives you a sense of normality and time with your baby’ (Mother, Discharge, #83)^24^ |  |  |
| *Returning to reality:* … yes, when we could take him out, when we could change him and feed him outside the incubator, then it felt more normal (3, p. 12)^5^ |  |  |
| *Involvement in care:* “To hold him and to care for him gave me warm feelings and contributed to stronger feelings of being a mother” ^41^ |  |  |
| *Formation of parental identity:* ‘You can feel a little more like mum and dad when you have him skin-to-skin–not a guest in his home’ [P5]^6^ |  |  |
| *Functional evaluation:* “I mean, by changing the position of the infant, saturation increases gradually. You know, when we put him in this position (pointing by her hands), saturation increased. As we turned the baby, the oxygen increased to 99 or 98-100.”(Grandmother)^42^ |  |  |
| *Working together:* “We breastfed first and then we tube-fed /…/because he couldn’t suckle so much. I prepared all the breastmilk; heated that; and changed the nappy and put him to the breast. He had a lot of cords, oxygen, and things. It was paper journals too, to document everything, I took care of that too.”^23^ |  |  |
| *Focus: From NICU to baby*: “Paul doesn’t do what the book says. If you read the book right next to Paul, you can watch him and he’s doing the opposite. There is the book of Paul and very few people know that book.^30^ |  |  |
| *Ownership: From their baby to my baby:“*I didn’t really feel like a parent at first. Especially the first couple of weeks when she was in the Isolette and she had all these tubes and wires and oxygen and vent tubes and NG tubes and central lines. And even though I have a medical background, I still always felt like I needed the permission of the nurses. This was more their baby than it was my baby.”^30^ |  |  |
| *Caregiving: From passive to active:* “I started changing her diaper right away and cupping her, so I touched her quite a bit, as much as they would let me. And then I kangarooed her when she was nine days old.”^30^ |  |  |
| *Initial lack of confidence in preterm infant care:* “Cup feeding was very difficult initially. I used to be worried if something happened to her or feared of choking and spilling. It was also very time-consuming as the baby sleeps too often while feeding. (M13)^39^ |  |  |
| *Confidence in preterm infant care/care enabling:* “It was difficult to change his clothes and to feed him. After providing care for a week myself, it is easier now. I can understand my baby and what he likes. Sometimes, he wakes up and provides facial expression, sucks his finger and I feel he wants to feed. I can interpret his cry in different situations. “(M11)^39^ |  |  |
| *Satisfaction and emotional comfort:* Now in SNCU, we visit, hold and care for our baby. I am doing KMC for about three hours, changing his diaper, and feeding him. The condition is improving. I am busy caring for him and am happier now.” (M9)^39^ |  |  |
| *From nervous and fear to calm and confidence*: “After a day or two of holding them, I gradually felt reassured. I felt like that everything was fine. As time went on, I no longer had to be so cautious, and my mood became calm and steady as well.” (P9)^17^ |  |  |
| *Awakening to parental roles:* I felt like a mother for the first time when I held my baby. Since I hadn’t had much contact with infants before, I felt so happy when I touched him. When I held him like this, really, I really felt like he’s, my baby.” (P15)^17^ |  |  |
| *Perception of parental role:* “After 10 years, God gave us a baby. No matter the costs of treatment, we will pay” (M4)^20^ |  |  |
| *Willingness to accept the parental role:* “I’m a mother. It is my right to know what they have done and what the outcome has been. Our opinions must be asked” (M5)^20^ |  |  |
| *Active caregiving with their infant:* “I would urge for more involvement of the parents, having me diaper, having me rub the swab milk and do the oral care, all those things are really important, especially for moms. I think we want to nurture, we want to care, and that's taken away from us for unfortunate reasons, and so it was very important for me to participate [in the FICare program].^34^ |  |  |
| *The father’s role in care:* I feel that they do not engage many fathers in training, because the father may not give a sign of how important it is to him^37^ |  |  |
| *Being seen*: ‘When it comes to childbearing today... It is like there is more focus on the mother than on a couple who have become parents, I think... It is more like the mother has had a child, and then the father is on the sidelines somewhere. That’s my experience’ (Father family 9)^43^ |  |  |
| *Gaining strength:* And I think I have become more secure, about what I believe in, and I know that just because I am not a physician, it doesn’t mean that I don’t have a sense of what is right or wrong, kind of, but it can be in other situations too, that you sort of have the courage to trust your own gut feeling you know, is this right or this is not ok, or this is how it should be, that you have become a bit stronger in that regard too’ (Mother, family 7)^43^ |  |  |
| *Fathers as secondary:* ‘I think they should encourage us more to come in the wards. I don’t see any men in the wards. Just having somewhere for us to sit’ (Father, Admission, #48)^24^ | Influence of NICU environment and Policies on Care Involvement |  |
| *Fathers as primary support person:* As a dad, there’s food supplied for Emily but not for me. In terms of encouraging dads to be here it makes it harder …. As dad, we don’t have much to do but support mum. The thought is put into supporting mum but not supporting the people who support mum’ (Father, Discharge, #29)^24^ |  |  |
| *Living beside reality:* The first week, it felt as if you never went out, couldn't get out, you went out for maybe five minutes and then back in again, you know. You became like the monkeys at the zoo nearly, wandering back and forward (2, p. 11)^5^ |  |  |
| *The NICU environment:* “Preoccupied by my surroundings and sometimes [I] would lose the ability to focus on the time I was spending with my son.”^13^ |  |  |
| *Easy access:* “[The neonatal staff] appreciate that there’s a bond between parents and baby that needs to be maintained. The biggest plus was… the (neonatal) unit got us a room over the road”. (4, father, D/C)^28^ |  |  |
| *Physical environment:* I liked to be able to sleep next to my baby once I finally was able to stay overnight in the hospital so that I could hear what was going on and be a part of the feedings. I think if I wasn’t able to stay in the room it would have taken a lot longer to go home because if you’re not in the room, then you can’t necessarily be there for the feedings and things like that. . . (ID 595)^15^ |  |  |
| *The NICU environment:* “The chairs are horrible, and the nurses were trying to put a pillow and make me comfortable. [. . . ] So as much as I wanted to stay there all day long, I was not comfortable.” (FG#2)^21^ |  |  |
| *Parental involvement policy:* It’s the hospital that has a policy of supporting mothers to take care of babies…The Sai-YaiRak Project promotes breastfeeding, allowing a mother to have a close bond with her baby and they pushed me to be here to have a chance to care for my baby. (ID, M1)^2^ |  |  |
| *Employee leave policy*: I have 3-month’s maternity leave, so I can devote myself to taking care of my baby. This helps a lot. I don’t have to worry about anything that could prevent me from visiting my baby conveniently. (ID, M14)^2^ |  |  |
| *Feeding:* Well, I was breastfeeding solidly, you know, but I recently stopped. I’m just trying to wean her on to formula because I’m going to start working and it’s going to be hard to work and pump. And I know they say that it’s the law for employers to allow you to pump but that’s just going to be so much work, and I don’t know if I’m ready for that. It’s a lifestyle choice, Breast feeding is not just something you can do like “oh yeah, I’m going to breastfeed now.” No, it’s something that you do that your life revolves around. Completely. It’s hard. It’s definitely a struggle.^22^ |  |  |
| *Visitation opportunities:* “Normally, newborns cannot be visited in that department, it is a chance for me to see my baby. I just want to come and have a look at my baby.” (P6)^17^ |  |  |
| *Long commutes and parking inconvenience*: “Sometimes, parking may take a long time, and there is no other way. My home is far, really quite far. It may take me about an hour and 10 minutes to drive there. The most annoying part is when I arrive, I can’t find a parking spot. I would have to drive around in circles there (to find a parking spot).” (P4)^17^ |  |  |
| *financial constraints*: “If the family’s financial situation is not good, we may consider saving every bit we could. We can tolerate not having a chance to hold the baby until discharge. The main issue is that the cost of KC is not covered by the medical insurance…It is hard for me to understand why we have to pay to hold our baby. It is very frustrating and incomprehensible. Anyway, we will see the baby sooner or later, so saving a little money is the priority.” (P7)^17^ |  |  |
| *Being So Close and Yet So Far Apart:* “It’s the worst thing I’ve ever experienced because I was just lying there alone, and I knew that 30 meters away was my family, and I couldn’t just go over to them” (M2).^27^ |  |  |
| *Structural factors*:“Yes, so what I noticed, of course, is the parking. If you go to the hospital parking and you are there all day, you can quickly lose a hundred francs or more a week. […] But other than that, we are not in a bad financial position. […] I think we should make it.” (F08)^18^ |  |  |
| *Resources:* I also want to give fathers three months, because the first time is the most important. […] The mother has memories with the child, but the father has to work all day and sleep all night. Then it feels like a two-hour visit home. But that’s the law.” (F09)^18^ |  |  |
| *The role of app technology in mFICare:* “I think, as you know, that time is relatively monotonous and relatively timeless, and so, the app not only kind of forced you to keep track of those activities because they're important but it also made you feel like you were doing something or accomplishing something every day.” ^34^ |  |  |
| *Inconsistent Access to Quality Translation Services:* I have something to say about the translations. Uh, the thing I didn’t like . . . it’s like they offer you phone translation, . . . which I think is really hard for the people that don’t understand English perfectly, I think [phone translation] doesn’t make a difference, seriously, because it’s really hard to hear and understand the phone translators, so I wish they could have had more [in-person translators] . . . I know they have some, but sometimes they’re not available, like one-on-one . . . so they can explain, you know, and make sure they’re understanding.^44^ |  |  |

**Table 3:** Synthesized Finding 3: The Influence of Parent-Staff Interactions on Parental Experience

| **Synthesized Finding 3: The Influence of Parent-Staff Interactions on Parental Experience** | | |
| --- | --- | --- |
| **Findings** | **Categories** | **Synthesized Finding** |
| *Participating in bedside rounds:* It kind of takes me back to one of the doctors that was doing rounds one morning and he asked me how it was going. I think it was one of the first days I participated in rounds and I went to go rattle off all the numbers that the nurse had told me. You know, how much weight she had gained overnight or lost overnight, how many episodes she’s had, how much food she’s getting. The doctor stopped me and said, “No. Mom, how is she doing?” He really made me think that even though the nurses are there for 12-hour shifts and looking after our little ones, I knew better how she was doing. (ID 586)^15^ | Inclusive Communication and Recognition of Parents During Medical Rounds | This synthesized finding shows staff play a central role shaping the parental experience in the NICU. The quality and nature of interactions between staff and parents in the NICU influence parental emotional well-being, confidence, identity, and caregiving involvement.  Parents reported inclusive communication, emotional support, consistent, availability, and encouragement from staff strengthened their understanding, confidence, and connection to their infant. These interactions contributed parents feel empowered, respected, and valued. In contrast, parents who experienced excluded, inconsistent, judgemental behaviour from staff reported reluctance to seek support, loss of confidence, reduced trust, and hesitancy to participate in caregiving. |
| *Parent-staff interactions:* “It’s very easy if they talked at the bedside of the baby, then you’re obviously part of the conversation, and they always asked me at the end of their discussion, they asked me if I had any questions and concerns.”^21^ |  |  |
| *Becoming a family:* There's been a good compromise between the professionalism of the staff, we know that they know what they have to do, but still, they listen to the knowledge and prior experience that I bring along about her development, her experiences (13, p. 4)^5^ |  |  |
| *Family-centred ward rounds:* “It was great to get that interaction, especially with the doctors, because they included you as part of the rounds, whereas I found before that they would kind of look at you but not talk to you. ^25^ |  |  |
| *Effective communication*: Some of the nurses were fantastic. Being available for rounds and being encouraged to be there when the doctors were doing the rounds was very helpful and everyone was very open and candid and answered any questions um so we tried to make sure that we were always there every day when they came around to do rounds. And that was great. (Interview 4, standard care)^1^ |  |  |
| *Communication:* Seven of the mothers said they had not been informed what was wrong with their baby and none said that they knew what treatment their baby was receiving. Just half reported being told about their baby's current health once or twice a day. Mothers felt it was challenging to ask for information, although many reported that it was easier to talk to the paediatrician when she did her rounds. ^4^ |  |  |
| *Academic and research participation:* “part of the medical team or part of the healthcare team.” (FG#2)^21^ |  |  |
| *Patient decision making on healthcare issues:* It is better that treatment personnel tell me what they are going to do for my baby. They should make sure whether we are willing to follow such treatment procedure for our baby and make use of my ideas as well. In this way, I can accept and encounter those decisions easier and will have more psychological readiness. (P10)^29^ |  |  |
| *Wanting to speak for oneself*: “I also feel good about learning many, many words, for example nappy, mother’s milk, nipple shield, blanket. I didn’t know those words before. I want to talk. I want to understand everything too. Swedish is very hard.” (M)^45^ |  |  |
| *Collaborative communication and decision-making:* the mother reported that she felt comfortable during the round and knew the structure of the rounds in detail. The mother felt that her observations of her infant’s condition were regarded as meaningful with regard to the medical decision-making. The participation and open information sharing provided the mother with a sense of control over her infant’s care, making her feel confident.^46^ |  |  |
| *Neonatologist-led communication and decision-making:* Parents revealed that they did not always understand the importance of their observations in the decision-making process. Instead, they emphasised their sense of being heard and respected and described the process of developing their expertise in the infant care. Many parents perceived themselves as not having competence to participate in decision-making and preferred to rely on the neonatologists’ expertise. . ^46^ |  |  |
| *Emergency communication and decision-making:* The interviews with the parents indicated that they had mixed feelings about their participation in the medical rounds. The situation was difficult for them because they had repeatedly received bad news about their infant’s condition and prognosis during previous medical rounds.^46^ |  |  |
| *Disconnected communication and decision-making:* The parents seemed confused about their participation in the rounds. They expressed feelings such as being under interrogation and worried about ‘asking stupid questions. Despite these feelings, the parents did not oppose their participation in the medical rounds.^46^ |  |  |
| *Being included or excluded as a parent*: It was weird, because it was my child who was lying there, so I wanted to know what they said; if it had been me who was sick, I would have been allowed to hear it; now there was not really anyone who could speak for him I was afraid that I was only getting the information that they wanted to talk about at that time. (M)^31^ |  |  |
| *Communicating through an interpreter*: “The interpreter that you book, you have to be sure that they have the right education and are able to interpret the information very well. If the interpreter, for example, says that you have a serious disease, it can be the baby or yourself. But maybe that’s not right, maybe the interpreter interpreted wrong. When you are shocked, you can be deaf to what is being said.” (P1)^32^ |  |  |
| *Using pictorial support:* “We got the pictorial support from the hospital to help with communication. To communicate, they fetched one of these and they said, “What you want, you can point to. They even had it in Arabic so that I could point to the picture. I had a schedule with pictorial support in my room at all times until we were discharged. Without the pictures, I would need an interpreter at all times, you could say.” (P2)^32^ |  |  |
| *Medical care team inclusion and connection:* “I just think being parents of any NICU child is really scary. So, if the doctors could take more time and just sit down with you. I understand that they have 50 other babies that they have to attend to and families and that kind of stuff, but I would just feel – if they could just sit down for a little, really explain to you what is going on” (ID 5)^47^ |  |  |
| *Perception of situation:* “I found doctors aren’t frank. My baby has many problems with his heart, brain, and breathing. Doctors decided to transfer him to the heart hospital for heart surgery, even though it was useless. The point is physicians do their best to save the baby because of their fear of the law but I don’t agree with a surgery” (F9)^20^ |  |  |
| *Hypervigilance and trauma trajectory formation*: “…there was one interaction I felt a little uncomfortable in with a genetics doctor… they had seen [my baby] and I had just come in… I walked right by [the HCPs] and they saw me go to [my baby’s] bedside, but they were talking amongst themselves and didn’t really introduce themselves or… proactively talk to me and I was really wanting to talk to them.” (F)^35^ |  |  |
| *Emotional issues*: ‘I was upset again because it was just a lot of sudden choices, decisions, and I feel like I wasn’t really included in the care as I should have been.^11^ |  |  |
| *Decision-making*: We went to go see the baby and the baby’s not in the NICU, and I’m like, ‘Okay. Well, where’s my child?’ And they’re like, ‘Oh yeah, he went down for a CT scan,’ and I was just like, ‘Why did he go for a CT scan? Like, we left, and he was fine. Like why suddenly?’… ‘Oh, we wanted to make sure he had brain swelling.’ And I’m just like, ‘This is not something to be kind of flippant about.’ So, I got ticked off, and when the MP came by, she kind of had the same flippant. And I was like, ‘Oh no,’ I said, ‘Ma’am, I need you to come back and talk to me a little bit.’ And she’s like, ‘Well, I have other rounds,’ and I said, ‘Well, I will sit here until you finish your rounds … So, when she did her rounds and she finally came back, it was kind of like,’ ‘Oh, you still here?’ I was like, ‘I told you I would wait because I need to speak with you about what’s going on with my child.’^11^ |  |  |
| *Caring for baby:* I was expecting to be just sitting there watching him you know, in the incubator with all the wires and everything, but I ended up being more involved. So, the nurses, they emphasized the involvement of my presence. . . like them just telling me to get involved instead of just telling me, “Oh step aside while I do this tube feed”. Instead of doing that, they kind of came and said, “Hey do you want a syringe to feed your baby on the side?”. Those little things made my day so much better. (ID 731 )^15^ | Encouragement and Coaching to Support Parental Caregiving |  |
| *Following the nurse’s advice:* …When I first entered the SNB, I didn’t know what I could or couldn’t do. It depended on the nurse’s advice. I didn’t dare do…because I didn’t know that if it is proper or not. When I arrived there, a nurse told me to change my baby’s nappy. Okay, I can do this. (ID, M14)^2^ |  |  |
| *Reassurance and encouragement:* “She kept willing us forward. ‘Why don’t you try this? Why don’t you do his nappy?’ I was like ‘Oh no’, very scared, but very encouraged to touch him, talk to him… Things around us were very positive’. (3, mother, D/C)^28^ |  |  |
| *Reconstruction of a normal family:* “From that day, Mrs. [X] encouraged me to do her chores up to now, and I feel like a mother. I mean, when I didn’t do her chores, I didn’t feel that I was her mother.”^48^ |  |  |
| *Personalized information and communication:* The NICU nurse encouraged us to participate in the care. We learned a lot by observing how nurses cared and by copying their practice” (M9)^41^ |  |  |
| *Seeking opportunity to be involved in caring for their babies:* I came here and went back home for two days because my baby’s intake of milk was only 1 cc. to 3 cc…When she got 15 cc. of milk I asked nurse’s permission to stay here. I felt that if I took care of my baby by myself, my baby would get encouragement. (ID, M1) ^2^ |  |  |
| *Perceptive engagement:* The nurse was the first one that said “Will you bath him? I’m like ‘No’, She said, “Oh, you’ve never bathed him?” I ‘m like “No, I didn’t know I could.” So, she says, “Do you want to?” and I said “No, I’ m scared “She said “I’ll teach you how if you want to”^49^ |  |  |
| *Cautious guidance:* They would teach me certain things, and then I felt more confident next time I could do it by myself and I didn’t need to worry about them standing there watching me to make sure that you know, they would say, ‘‘This is how I like to pick him up out of the isolate. I’ll show you, and then you can try next time by yourself, so you don’t have to ask for help each time.’’^49^ |  |  |
| *Engaged parenting to partnering:* We had one nurse who was teaching me early on about preemies. That their tone isn’t good. They can’t get themselves tucked up and curled in a fetal position. You have to help them. She’s just now getting to the point where she can stand a little bit more stimulation. And the nurse she has today and I were talking about that earlier. What should we maybe be doing now that she’s starting to be awake and alert a little bit more?”^30^ |  |  |
| *Creating other solutions:* “For example, this picture was not there (pointing at a picture of a baby being bathed brought by the author to the interview); ‘shower the baby’; this picture was not there, and every week we had decided, but when she talks; I don’t understand, but when she says “Shower” and points to her head, then I understand.” (Parent 2)^32^ |  |  |
| *Mixed experience of guidance and support for preterm infant care:* “It has been 3-4 days since I began to hold and feed my baby. They have instructed me to spoon-feed and breastfeed him alternately and continue the same after going home. I am still not confident to feed my baby yet. It would be easier if I could learn how to feed him properly before being discharged. After going home, my mother-in-law might help in feeding and other baby care.” (M6)^39^ |  |  |
| *Parent-to-parent support for learning preterm infant care: “*Observing other mothers’ doing, it was easy to follow when nurses helped KMC. Now I am feeding him, providing KMC as guided. (M16)^39^ |  |  |
| Breastfeeding support: I was taught how to breastfeed initially when I came here…. (M2)^8^ |  |  |
| *Inclusiveness and guidance from nurses*: “It’s our first child, and we’re inexperienced with newborns. Her fragile and petite nature is quite intimidating; there’s this overwhelming fear of causing harm just by holding or even touching her. Yet, watching the nurses handle and care for them reassures us. I paid close attention to their interaction with [son’s name] and other infants on the ward, trying to learn the right approach. The desire to hold and embrace him is profound, but the fear of inadvertently hurting him is equally strong. Seeing nurses in action and their consistent assurances that it was safe, and even beneficial for [son’s name], offered immense relief.”^50^ |  |  |
| *Enhancing caregiving self-efficacy:* “The nurses are here with me, teaching me things, providing guidance on how to hold the baby, how to feed, and how to change diapers, I gradually used to adapt to these tasks. They also share useful tips with me.” (P3)^17^ |  |  |
| *Parent-professional interaction*: It would be good if you could be there and do this.” We took that to heart and had to say: “That’s right.” […] That’s certainly why we came more often […] We knew that if we weren’t doing well, we didn’t have to. We have support here. We felt that.” (F05)^18^ |  |  |
| *Coping and support:* ‘One of the things that I could say, is they were encouraging me to breastfeed, to nurse because they were like, ‘Your baby needs it.’ And so, I had regular consultations with, with the lactation nurse, and she was – she was working with me on how to pump and how often the pump and to keep trying and how to massage my breasts, and she was very hands on. She even put her hand down my shirt, and she was working with me until I was able to until my milk dropped.^11^ |  |  |
| *The interaction between the medical staff and the family*: One of the nurses did her best and used all her skills so that I could do breast pumping, and every word she uttered encourage me.^37^ |  |  |
| *Engaged parenting to partnering*: “Everything depended on what nurse you had; how quickly in the shift we could get in here and develop a rapport with her, and how controlling they were.” ^30^ |  |  |
| *Taking on parental responsibility:* ‘‘Sometimes you just can’t see any nurses. It is comfortable, when you can see a few’ [P12]^6^ | Staff Familiarity and Presence Supporting Parental Involvement |  |
| *Comprehensive advocacy:* “Tell me, don’t you want us to help you? I say I will get it myself, but I see them looking at me from a distance to look out for me. So, I have nothing to worry about when I need help. Her nurse is there to help me.”^42^ |  |  |
| *Subtle presence:* They gave you privacy; you know what I mean? They put the curtain around; they didn’t bother you if they didn’t need to. If the bells and whistles weren’t going to, they just left you to just be with your child.^49^ |  |  |
| *Feeling trust and confidence:* “Every time I talked with them (healthcare staff), the pain was relieved. They were really kind . . . I was comforted by them and able to hug them; it felt like they were part of my family.” (P16) ^32^ |  |  |
| *Appreciated competent and affectionate care to preterm infants*: “I was unknown of caring for such a small baby. I saw such good care there. We can’t give that much interest to others’ babies. But they cared for babies so well that it looked like they were caring for their own babies. I was surprised. They were prepared for such good care.” (M3)^39^ |  |  |
| *Unmet Family-Identified Need for Nurse Continuity of Care and a Meaningful Relationship With Nurses*: Just having primary nurses felt really good. I felt so much more . . . I mean, I know all the nurses were competent . . . but anytime my primary nurse was on duty, I felt like I could relax a little bit more, you know? And I felt like they cared about my babies more and would look out for them more even though, you know, all the nurses have to care for all the babies. It just emotionally felt safer^44^ |  |  |
| *Comprehensive support:* “Some nurses really took care [of my baby) very well. That was a weight off my mind. For example, when I was going home. I was feeling that if I wasn’t there, she was taken care of very well.”^48^ | Parental Trust Staff Expertise and Supervision |  |
| *Experience and confidence:* “All the doctors that were there, as far as I’m concerned, were the experts. The doctors had, you know, been in this industry for like 15, 20, 25 years. They knew, they knew their stuff inside out you know, the information was never flaky…” (16, mother, D/C)^28^ |  |  |
| *Deference to the expert:* To be honest we had a baby three months early and she’s tiny and, in an incubator, so you do whatever the doctors say at first because they’re the experts. (Rachel)^16^ |  |  |
| *Seeing progress:* The biggest joy was seeing how quickly my son was able to overcome the obstacles of being preterm. He was able to start eating a lot better under their [nurses’] supervision and as a result he got stronger. That was a great joy to see him get so much stronger so fast under their care. (ID 152 )^15^ |  |  |
| *Synergistic care as partners*: “A mom can blame herself for being away from her baby and not being able to do anything for the baby. But the HCPs gave the baby all the attention and love, they fed him, changed his diaper so the baby wasn’t neglected and was raised well, which compensated for my lack of doing anything as a parent. I think that is the most ideal and most important outcome.” (P1)^33^ |  |  |
| *Pain management*: I normally express some breastmilk for them(nurses) or sometimes they will call me to come and hold my baby if the baby is crying. (M3)^8^ |  |  |
| Nurses’ personal attention to the babies: “I tried giving him a pacifier, but he kept refusing it. A nurse approached us, explaining that he favoured a different teat type, and handed it to me. He accepted it instantly. This gesture reassured me, signalling that they were genuinely observant of his needs, not solely focusing on medical care.”^50^ |  |  |
| *Level of health literacy:* “I like to participate, but I don’t know about diseases. They do what is better. I’m pleased. The doctors know better” (M4)^20^ |  |  |
| *Distrust and fear of the medical setting:* “Every day they would ask me if I went into labour that day, would I want to just be sent to [the hospital with the level IV NICU] to give birth to [my baby] if it came to that point so that I could be with [my baby] in the hospital… in tears all of those days, I kept telling them you’re going to have to take [my baby after he’s born] because I don’t know that medical team and they could kill me and I want to be alive to meet [my baby]. And so, if that means that I have to birth my baby'.^35^ |  |  |
| *Impact on relationships: “*builds trust, like you really need that with this situation. Like, if you can’t have a communication, I don’t know how you’d be able to leave your baby in the room and walk away”.^38^ |  |  |
| *The value of staff engagement:* It was one of the nursery nurses sat down with me and explained what it [Train-to-Home] was and did the first round of stickers and then another nurse went through it again a bit later, updated it with a different discharge date and everything. (Mother #219)^7^ | Staff Communication and Explanation to Promote Parental Understanding |  |
| *Getting to know your baby:* It kind of gave us a guide to what we should be doing and what we should be learning and looking for. (Mother #617)^7^ |  |  |
| *Information and explanation:* She explained every machine to me: ‘That’s to monitor her heart rate’, she goes ‘Your baby’s not on oxygen, she’s on air just to help her lungs, 'cos you do know baby’s really small. Baby’s not sick mummy.’ And she explained everything to me. The machines, how they incubate her.’ (30, mother, D/C)^28^ |  |  |
| *Fathers and families loved it:* One of the nurses sat down and went through everything so that we completely understood it, and it was great because we’ve got a four-year-old, he loved the train, he thought it was fantastic, and it was great for him to be able to understand a bit more… (Mother #418)^7^ |  |  |
| *Participation and dialogue before and during Early discharge program:* “Felt very much that there was space and time,” and “The [number] of conversations we had was just right.”^27^ |  |  |
| *Lack of parental involvement guidelines:* A nurse didn’t teach me how to change a diaper… I didn’t know how to it at the first time, so I observed from an old one that a nurse did. (ID, M16)^2^ |  |  |
| *The professionals were supportive but also created a sense of insecurity*: He was more or less happy with the care but felt that the staff did not devote enough time to informing them and making sure that they had understood the information" (F36)^12^ |  |  |
| *Communication:* “It was a busy environment, and so if communication had been bad, I would have said, ‘I can appreciate why it was bad’, but it wasn’t, it was really good, so communication for me was number 1. Absolute number 1. And that really helped, that felt, made us really reassured. It gave us confidence throughout the whole experience. Really good”. (1, father, D/C)^28^ |  |  |
| *Do it right at the beginning:* We did think it was a really good idea that you can see him progressing, but it’s just unfortunate that we didn’t do it right at the beginning…..So we didn’t actually see much progress. That’s one thing I suggest…is to do it right at the beginning so that you can see a change. (Mother #408)^7^ |  |  |
| *The challenges of expressing breast milk:* “I kept asking, when do I start expressing, I I’m rubbing the, trying to get it to come out but nothing’s working um… and it was about day 4, I think before they said to me, oh yea, here’s a kit, go and express”. (21, mother, D/C)^28^ |  |  |
| *Facing misunderstandings:* “In certain situations, it happens that we misunderstand, and we are trying to find a proper conversation so that we can explain ourselves and why something has happened, for example, why I was half an hour late in feeding the baby, because she was sleeping so soundly. I just simply say that ‘she was sleeping so soundly’. The nurse would not understand; she was like, ‘You should wake her up, you should feed her. You know, these times are very important, every three hours she should have a feeding.’ Then I also have to explain to her that, you know, ‘she was very sleepy’. I went to the other nurse, I explained to her, and she said, ‘Wait another half an hour; she will wake up if she is hungry.” (P6)^32^ |  |  |
| *Analysing silent language/communication*: “There are certain things that are similar in all languages when it comes to body language and signs; for example, when they told me to wait or explained to me that it was time for breastfeeding, these kinds of things I could understand when they were instructing me.” (P12)^32^ |  |  |
| Content with informational support: “They told me many things that I need to be careful, e.g., for breathing pattern, warmth of the baby, stimulate the baby if bluish discoloration of the baby’s face. They also instructed about Kangaroo mother care to keep warm and expressed breast milk (EBM) feeding for the baby.” (M13)^39^ |  |  |
| *Strengths:* “You know we’re getting someone like 100% focused when we’re talking to them” (interview 6)^51^ |  |  |
| *Communication and clarity about infants’ health conditions and progress:* “When the situation was still uncertain, and we weren’t sure about the possible consequences, the doctors and nurses would explain how things were progressing, and it was clear. But I felt the need to hear the updates multiple times from various people. I would consistently ask every nurse about [son’s name] condition and sometimes even ask another nurse to reconfirm what I’d heard, even when I knew I’d understood correctly. It seemed like hearing ten people say things were going well would genuinely reassure me. However, deep down, I felt that even if a thousand people gave me the same positive feedback, I wouldn’t truly be at ease.”^50^ |  |  |
| *Passive acceptance:* “I came here to do KC, but I only saw a sign when I got to the entrance. Honestly, I’m still not entirely clear what KC is. When the doctor told us that I could start KC, I had no idea what it was. We even asked a friend what the benefits of KC were.” (P7)^17^ |  |  |
| From unmeasured to gratitude: “When I have close contact and communication with the healthcare professionals, I could know more about them… I can ask the nurse about my infant’s condition like how much milk he’s drinking, and the nurse patiently tells us, so we feel more reassured and very grateful to the nurses.” (P10)^17^ |  |  |
| *Parents reject caregiving due to perceived inequalities:* “Here, the nurses told me to change my baby's diaper. Why should I take care of my baby when they get paid to do it?”^10^ |  |  |
| *Getting Information About the Newborn at Different Times Intensified Feelings of Separation:* “I would rather it was the nurse who told [mother’s name] how it should be done than it was me who had to sit and be more knowledgeable” (F2)^14^ |  |  |
| *Confusion regarding NICU care:* “Honestly, to this day, I still don’t really know why he was admitted” (ID 5)^47^ |  |  |
| *Discharge readiness:* They did rounds on Monday and was like oh she’s going home tomorrow, so there was literally no time for me to prepare mentally, physically, or anything like that” (ID 36)^47^ |  |  |
| *Methods of communication:* “I wish they gave us some information to take home versus just going over it.” (ID 9)^47^ |  |  |
| *Earn rather than assume trust:* These people came in’. And she would talk to me and it made me feel more comfortable to be myself, and I didn’t feel like I was on eggshells or judged or something.” (M)^35^ |  |  |
| *Communication and information sharing*: ‘They had a flyer like a poster. It was called my flight plan home to where we were supposed to check off different things to learn before going home, but nothing was ever taught.’^11^ |  |  |
| *Physicians in possession of detailed information:* Hmm … it’s quite disappointing at times. This morning, for instance, I needed information about my baby, but because the doctor wasn’t around, I couldn’t get the information, I felt sad [Adwoa, Immigration officer, 31 years, FGD]^36^ |  |  |
| *Being understood:* ‘Precisely those feelings I know everybody talks about, that it is so wonderful, and it is so good, and you love the baby from the very first second and all that stuff. I didn’t feel that, it had to develop slowly and I thought it was so relieving to talk to them about this and get their feedback that it is not so unusual to feel that way’ (Mother, family 3)^43^ |  |  |
| *Lack of knowledge/ambivalence*: There definitely should be a NICU social worker [but] for us, we had no idea that we were eligible for Medical. We needed the social worker to tell us that. So, I think that’s really important for supporting families later so that they don’t get . . . a huge bill . . . [but] we didn’t get told about Medical until a few weeks later . . . For some reason, [the social worker] didn’t tell us about the medical program until later . . . I don’t know what the reasons are, but she was checking in regularly. We didn’t realize that we needed to ask for the information, you know?^44^ |  |  |
| *Conflict:* One thing that bothered me is that [social workers are] very quick to call CPS. They don’t call the parent if they miss an appointment or what have you. So, we had a follow-up appointment the same day I took him to the ER and the social worker called CPS. Like, they didn’t notify me, say, “Hey, you missed your appointment,” you know, or anything. Strictly called CPS. “But you have my phone number . . . come on, guys. Why didn’t you call me? You have my number.”^44^ |  |  |
| *Perceived status of problem solving:* “I felt that they treated me rather differently, especially because they knew that I was emotionally vulnerable and in need of care.”^42^ | Individualized Emotional Support and Respectful Care |  |
| *Feeling like an individual:* “Yeah, I just found our experience very good, it was very, I suppose, personal in a sense. I wasn’t, I didn’t feel like a piece of meat. I felt like a human that was passed around and people were caring…they were really willing me on and I felt like a person that was going through this and I had support around me rather than: you’re in a hospital, you’re passed from buck to buck”. (3, mother, D/C)^28^ |  |  |
| *FICare Enhances parental confidence and parental role attainment:* “It was great to have someone there to hold your hand.”^25^ |  |  |
| *Reaching a deeper level of communication:* We talked all the time [in the unit], but that was different. We discussed subjects at a deeper level [in the dialogues]. Yes, we discussed totally different subjects than in the unit everyday life.’ (GFCC-F5)^52^ |  |  |
| *Supportive/trustworthy healthcare professionals:* “One nurse, she snapped me out of my postpartum. Just as — you know, no matter what anybody was saying to me, it was just — it was constant, I was blaming myself. But her, she was like — to me, she was like my angel. She came in, she’s like, “No, that’s enough. You’re doing perfect, that’s more than enough.” (FG#1)^21^ |  |  |
| *Emotional support:* “It was nice to tell them my story and to have somebody who was just listening and who understood the situation on the NICU.” (M9)^41^ |  |  |
| *Sensitive and emotional support:* “It was almost overwhelming how lovely everyone was and just, before during and after, the whole process just sympathetic and, and came across like they were hurting too… [The staff were] incredibly empathic and you know even, they were giving us the, the prognosis with Z, and they had tears in their eyes you know the way that they were saying it and you kind of, it makes it feel like you’re a person who is experiencing a terrible thing rather than just another number going through the process”. (32, mother, dec)^28^ |  |  |
| *Reconstituted family:* “Every mother calls her baby with words like chubby or nice, and everyone believes their baby is the cutest one; but I could not experience this feeling not at all (with emphasis). Her nurse was the only one to help me.”^42^ |  |  |
| *Meta-family interaction:* “Every one of these nurses was considered to be a main member of our family.”^48^ |  |  |
| *Met in the home with respect and understanding of the family’s overall situation:* “Feel very much that we were treated with respect and understanding of all aspects of our situation”^27^ |  |  |
| *Informational privacy:* I don’t like people other than the treatment personnel (secretary, service staff, Mothers of other hospitalized infants, their relatives) in the unit who are not main health care providers for my baby be aware of my baby’s problems and his genetic disease; I prefer no other person except his doctor and nurses access information on my baby’s disease. (P11)^29^ |  |  |
| *Psychological privacy:* The treatment personnel are only allowed to talk about my baby’s problem to me and my husband and they should never disclose this information to anybody else. In such sensitive situation, I don’t even like my sister knows about my baby’s disease. (P15)^29^ |  |  |
| *Empathetic behaviours:* When we empathize with these mothers, they without fear of judgment and loss of dignity tell the health care providers your concerns and problems and subsequently experience better feelings. (P6)^29^ |  |  |
| *Meeting a fellow human being:* The doctor listened, the doctor was also a person she showed that she was also a fellow human being in the whole thing; she said, but God, here I am, saying horrible things to you, but of course I have to say what I say now. (F) ^31^ |  |  |
| *Mixed experience with communication and emotional support:* “We expect a polite response from nurses like the way we talk to them. But it doesn’t happen like this. Some are nice but not all. Some of them didn’t answer our questions. Then I felt like something bad was happening to our baby. I didn’t discuss it with anyone or say a word because our baby is inside there.” (F4)^39^ |  |  |
| *People:* “from the nurses I felt so much care and compassion than from the [physicians] . . . I try to join the when the attendings are rounding. It’s different, obviously, because they’re doing their job, but there’s, it’s still the information” (interview 1)^51^ |  |  |
| *Support families and provide resources: “*I just think that [I] could have been supported before it got to that level… There should just be somebody that just says ‘I’m here [for] you, [you] want to scream, you want to cry, you want to yell? I’m here… to tell you, you’re going to be okay. Even if your baby doesn’t make it, baby, you’re going to be okay.’” (M*)*^35^ |  |  |
| *Provide compassionate care:* “I would have people of color in the NICU … that might make people feel a little bit more comfortable, like I’m not surrounded by … people that don’t look like me.”^35^ |  |  |
| *The family’s need for spiritual support:* I think nurses are very helpful and can show more sympathy and understanding for a family.^37^ |  |  |
| *Confrontation of caregivers:* Although I would like to stay and calm my baby, I have to leave the ward because they do not want to be disturbed while working.”^42^ | Staff Behaviours that Undermine Parental Confidence and Emotional Safety |  |
| *Staff as gatekeepers:* He was upset so I was rubbing his foot and hand. The nurse came in and growled at me to try not to disturb him’ (Mother, Admission, #61)^24^ |  |  |
| *Feeling like a burden:* ‘We felt not to bother them like kindergarten children. But it would’ve made us more confident and comfortable.’ (Mother, Admission, #39)^24^ |  |  |
| *Passive recipient of care:* ...Nurses were working…When I asked them, they told me to wait a moment. I visited my baby, I often saw them keeping busy…I felt hesitant and afraid to ask them and I thought if they were available, they would come to me. (ID, M13)^2^ |  |  |
| *Power struggles:* And they said, “you don’t need to concern yourself with that, we need to concern ourselves with that”. And I was like, actually I don’t agree, I think I need to know that as well cos I’m his mum.^16^ |  |  |
| *Muted relations:* I wouldn’t talk to them [nurses] about feeling upset or depressed or if I had worries about her or anything, in case they thought I was some kind of psychotic mother. I’ve never felt. . . . I just couldn’t speak to them about anything personal or anything like that. (Jane)^16^ |  |  |
| *The professionals were supportive but also created a sense of insecurity:* She felt that some of the staff members on the night shift did not understand her and that they thought she was a bad mother for not being able to cope. (M40)^12^ |  |  |
| *Interaction between staff and parent attendants was based on helpful communication although moderated by inherent hierarchical parents that differentiate compliant and non-compliant parents:* Yes, the nature of everyone here is quite good. They scold us sometimes, but it doesn’t matter because it is for the betterment of our baby, so it’s okay.” (Father 02)^26^ |  |  |
| *Confronting problems of breastfeeding:* …I know that my baby must get milk, but I don’t want to force him. When my baby gets a little milk…It’s like two-sided pressure. A nurse pressured me to force my baby. He doesn’t wake up even after I try to wake him. I need to force him, is it right? (ID, M9)^2^ |  |  |
| *Avoiding discrimination*: Sometimes it happens that health care providers discriminate against parents during taking care of infants and interacting with their mothers. Some health care providers do their job better, with more observation of health care providers principles for infants whose parents have higher educational or financial situation; they even answer their questions more respectfully. Such behaviours threaten other mothers’ dignity in the unit. (P17)^29^ |  |  |
| *Poor support for maternal involvement in care:* I have been able to know certain things about my baby through my conversation with some of the staffs. But some nurses are not being friendly. I hope we will be discharged soon. (M4)^8^ |  |  |
| *Challenges:* “the first day . . . we had zero information” and “I did ask for someone to come down, for the doctor to come and speak to me . . . no one came” (interview 1)^51^ |  |  |
| *Being Emotionally Divided in the Relationship With the Nurses:* Our relationship and bond of trust is with the nurses around us, who were in the NICU, but they do not care for me. So, when I say, I feel really bad . . . some strange nurse comes over [from the maternity unit] and looks at me, and she has had no relationship with me. (M4)^14^ |  |  |
| *Mothers would not be able to learn new skills:* ‘Just like in class, some children are sharp. When the teacher teaches, those sharp children pick up faster. Some learn but they are slow learners, and some, when you try to teach, you are just pulling them.’^19^ |  |  |
| *Healthcare providers would not trust maternal assessments:* ‘the doctor sees that it's not a big deal and yet you see it's a big deal.’^19^ |  |  |
| *Impediments to mFICare delivery and meeting parental priorities:* “There were a couple times where I really wanted to be part of the FICare briefings [rounds] in the mornings and it would just get started earlier than normal or they wouldn't call me on the phone. Or the breastfeeding, like they kept telling me how important breastfeeding was and then I would call and say, “I'll be there at 9 so don't do a bottle feeding or a tube feeding because I'll be there at a certain time,” and then I'm literally like five minutes late and I call and say, “I'm five minutes late, I'm on the bridge,” I get there and they're like, “Yeah, we already fed the baby, we just had to move on.”^34^ |  |  |
| *Staff judgment or dislike of families:* They were just, like, snobby and their body language, they had attituded all the time. This mother and her husband returned to shift work in fast food while their infant was hospitalized. Instead of feeling empathy or receiving support from the NICU care team, they felt judged and blamed as bad parents due to their socioeconomic status.^44^ |  |  |
| *Racially based judgment*: I didn’t know until I looked at her medical chart because I needed to apply for social security and that’s when I see all the comments about me made . . . And they made me feel bad because I couldn’t breastfeed a lot and I couldn’t transport breast milk because it would get bad by the time I got there, and they didn’t like that.^44^ |  |  |
| *Respect family concerns: “*Not making advocacy for your children a crime… when [parents] has the courage to speak up about something, making sure that they feel heard and not crazy.”^35^ |  |  |
| *myth of Black “hardiness:* “I’m bawling in a car hoping these [HCPs] love my baby enough to keep him alive [during] surgery, my little brown baby.” (Mother)^35^ |  |  |
| *Policing and surveillance:* “The most difficult thing was feeling like a prisoner, like the baby didn’t belong to me. I had got that feeling every time I came.”^35^ |  |  |
| *Undermining black parenting:* “I never felt a part of the team. Anything I said was invalidated. None of my concerns were real or genuine, apparently, to the health-team.”^35^ |  |  |
| *Biased provider’s attitudes*: ‘I asked for breastfeeding, there was no breastfeeding support given. I had to teach myself how to use a pump and struggled often. And their reasoning for me is like, “Oh, you don’t have to worry about it now. They’re small enough to get donor milk, so you don’t have to worry about it.” So, it was a reason as to why they didn’t provide the help despite me asking for the help explicitly.’ ^11^ |  |  |
| *A healthcare Worker’s mood at a specific time:* Sometimes I am satisfied with the response I get from them, but not always. And mostly, it depends on their mood. They are humans in any way, but if you come on a day, they are moody, you will see they hardly like to talk to you. This is bad in any way, but what can you say? [Adzele,27 years, Military officer, IDI].^36^ |  |  |
| *Fathers feeling excluded, judged, and unwelcome:* Interviewer: What were the moments where [your husband] felt judged? Um, numerous times when my husband wasn’t around, they asked if he worked. It was just very weird. So, finally, my husband wrote on the board, “I work at Folsom Prison. Not an inmate there.” . . . I would just say there were stereotypes about him. My husband is the complete opposite of any of those, so I think that’s probably what makes him so frustrated . . .^44^ |  |  |
| *Continuity and consistency in care:* The one thing we found that was a little bit hard was when there would be a new nurse, it was like starting again from scratch. They would assume our knowledge was very low and would repeat a lot of the same thing or try to tell us the same thing. . . So the more they could keep the same person [nurse] with us, the easier it was and more beneficial it was for us. (ID 225)^15^ | Inconsistency in Staffing and Its Impact om Parental Experience |  |
| *Consistency in care and caring staff:* If the same physician was in for two weeks, you get momentum going and you would kind of be — you’d have a plan, more of a long-term plan. When someone would come in on the weekend, sometimes they would totally switch gears on you. And then, whoever was there would come back the next week, it would sometimes shift again.” (FG#3)^21^ |  |  |
| *Consistently inconsistent:* To me it’s just about consistency and every nurse does things differently. One nurse will tell you to do one thing, and the next nurse will come in and criticise you cos they wind or feed a baby differently and it makes you feel like crap. (Hannah)^16^ |  |  |
| *Fathers’ satisfaction with nurses’ support for mother: The* staff here are outstanding, and we believed everything was excellent. But the guidelines for parental care, like how long to wait before bottle-feeding our daughters’ post-bath, varied among nurses without clear general instructions. This inconsistency was sometimes perplexing, especially for [partner’s name], leading her to feel uncertain and questioning her maternal capabilities.”^50^ |  |  |
| *Nurses’ varied personalities:* There’s a rotation of nurses here; you meet one today, and a different one tomorrow. Some are naturally cheerful, and others are more reserved. Some nurses, when they can, take extra time to discuss how [children’s names] are doing, while others are more succinct. Yet, they all demonstrate proficiency and genuine concern for the babies. Whenever we have questions, which is frequent, the staff is always approachable.”^50^ |  |  |
| *Being Admitted to Two Different Units with Two Different Agendas:* [The NICU] was very keen that the mother should also begin breastfeeding and breast pumping, whereas the other unit, well, it’s blood pressure. Uh, you’d better not pump. . . . We actually had to try to conjure up a bit ourselves . . . unknowingly. It was chaos. (F3)^14^ |  |  |
| *Revolving clinicians:* “There’s people coming in and out all the time, and you have one doctor on Saturday morning, and you never see them again, there’s another doctor on Saturday night, you never see them again [ . . . ] you develop a sort of little relationship with someone and then you never see them again.”^38^ |  |  |
| *Impact on personhood:* “I feel if they are primary, they feel like they own it, if they are not primary then why bother to know the baby or, it’s more of an attitude with the baby. I don’t have to know the baby, I am just here, I just have to take care of them and go.”^38^ |  |  |

**Table 4:** Synthesized Finding 4: Psychosocial and Relational Coping Experiences of Parent in the NICU and Beyond

| **Table 4: Synthesized Finding 4: Psychosocial and Relational Coping Experiences of Parent in the NICU and Beyond** | | |
| --- | --- | --- |
| **Findings** | **Categories** | **Synthesized Findings** |
| *Discovering and expressing emotions:* ‘[Using reflection sheets] helped us put into words some of the things we actually felt inside. It felt good to express some of the chaos, because it is chaotic having a baby born that early.’ (GFCC-M7)^52^ | Coping Strategies and Psychosocial Support | This synthesized finding reflects the ways in which parents cope with the psychological, emotional, and relational challenges of having a baby in the NICU and transitioning to life after discharge.  Parents experienced a variety of coping strategies including emotional expression, spiritual coping, peer support, and reflections to overcome emotional toll during hospitalization.  Partner and family dynamics were impacted by separation and role disruption, which influence parental well-being and caregiving capacity.  Parents experienced mixed feelings during transition to home. While some parents reported feelings of independent and comfort, others experienced overwhelm. |
| *Obtaining mutual understanding:* ‘It was a process for us to talk through the questions in the reflection sheets. I think we were totally unaware of some of our differences . . . we knew there were differences in our perceptions, but being forced to reflect on it, put it into words, that was what I experienced as most valuable afterwards.’ (GFCC-F2)^52^ |  |  |
| *Coping with daily disruption:* Being away from my husband and my son was one of my biggest challenges. . . I talked to my husband every day but it’s not the same. . . It was harder not to have his support physically than just on the phone or through text message. (ID 679 )^15^ |  |  |
| *Mental health:* “There should be a team to comfort mothers because many of them arrive depressed and they don't know how to pick themselves up.”^4^ |  |  |
| *Group education sessions:* “I deliberately [went] to meet other people. that’s where the education sessions were good for us.”^25^ |  |  |
| *Miracle babies psychosocial support:* “Someone who’s actually come out the other side … can come in and … say that yes, okay, it is hard, but you do get over this and you actually can move on.”^25^ |  |  |
| *Coping strategies:* Prayers, crying, taking time off work and receiving occasional assistance from the staff were among the coping strategies used by families to deal with the anxiety associated with the ICU phase of treatment^51^ |  |  |
| *Praying to the holly idols for their child’s well-being:* My baby slept without any clothes on (in an incubator). He was breathing but I wondered if he would survive. When anybody called me, either my relatives, father or sister, I asked all of them to make a vow to the holy idols for me…In my hometown, we have San Klang Ban (the shrine of the village) that I pay much respect...When I did that, I felt very relieved. (ID, M18)^2^ |  |  |
| *Coping strategies:* “And so we said very clearly we’ve done this before. This was what happened . . . and we think it could happen again and we just want to be very conservative” (interview 4)^51^ |  |  |
| *Respecting religious identity* I’m a religious person. I invoke to Imams a lot. I liked to put the green piece of cloth brought form Imam Reza (PBUH) Harem in my baby’s bed. So, I talked to my baby’s doctor and nurse and they respected my religious beliefs without prejudging me and accepted my request. (P19)^29^ |  |  |
| *Dealing with administrative barriers*: “’Cause when I buy the medicine from an international market, I found that there are many languages; the whole-country language is there, but English is always, always, always there. So many times, it happens here that we do not have that in the instructions. For that particular medicine, I have to search in Google. Many of them (other immigrants) are not so educated, they just blindly follow what they understood, and sometimes it can be harmful for the child. So, I think it’s very important to add more languages in the product information.” (P6)^32^ |  |  |
| *The need for psychosocial support:* “We had our children after we had been living here for some years, so we were not completely blank when we came here, but we didn’t understand as much as now.” (P9)^32^ |  |  |
| *Compromising on Own Needs:* “I dropped it simply because I couldn’t handle all that porter and long waiting time” (M1).^27^ |  |  |
| *Religious beliefs:* “Our decisions are based on our beliefs. Religion is the light of our path of life. I believe in God. Where the decision is crossing the religious rules, I draw a red line there” (F8)^20^ |  |  |
| *Emotional and social support:* “It was a good place for my husband when a lot of times the men kind of just fall through the cracks, you know, in terms of how they're feeling, but the class was good because there were other dads there and the nurses would ask us to sit in a circle and talk about like our greatest fears or what our babies were going through, and it was a time where he could express himself in a safe place.”^34^ |  |  |
| *Improve mental health resources:* “I would… have a therapist [in the NICU], mothers are going through this traumatic experience.”^35^ |  |  |
| *Emotional and mental toll of having a preterm birth:* ‘post-partum depression is real. I think probably a year, I may even say two, parenting was really a blur particularly the adjustment from coming straight from the hospital to home.’^11^ |  |  |
| *Relationships with other NICU moms*: ‘Yes. On the times that we even had a brief conversation [NICU moms]. It was helpful because it was hopeful to let us all know that we’re all going through something similar. It’s all different situations and diagnoses, but we were all there to embrace our children.’^11^ |  |  |
| *Processing experiences*: Well, you go back to all these things that potentially could have happened, you go back there...It is rather challenging in a way. But that is a part of the working through of course... That was really good because then you processed, so that was really good. I think that is the point, that you are able to talk things through and sort things out’ (Father, family 11)^43^ |  |  |
| *Help to move on*: ‘You haven’t suppressed anything, and you haven’t hidden anything, and you can kind of talk and handle things, and that I think has been an incredibly good foundation for the new challenges we are in the midst of just now, otherwise it would have been very difficult. That it just doesn’t escalate, but that you have been able to overcome the initial days and weeks’ (Mother, family 6)’^43^ |  |  |
| *Family life:* It was just I think fatigue, fatigue played a major part of it ... I mean my wife and I were always very close, we were, but it definitely didn’t separate us. And I think it did make us a little bit stronger ... because we realized that the team work is going to make the dream work here. We’re either going to live by the sword or die by the sword, but we can’t be two separate entities. (Interview 13)^1^ | Family and Partner Dynamics in the NICU |  |
| *Ambivalent relationship to the partner:* She felt that her partner did not understand how it affected her not being able to sleep at night (M40)^12^ |  |  |
| *Emotional state:* “I’ve been told nothing to my wife about our baby’s condition. The situation is stressful for a woman. Mothers may act emotionally” (F8)^20^ |  |  |
| Alignment to the needs of their partner: The father helped by taking charge of the gavage feeding, as this was something that caused the mother stress (F8) )^12^ |  |  |
| *Family support:* I just told her (the preterm baby’s mother) how to take a bath and what kind of milk to feed a baby…She should pour some water on the face first to prevent a baby from getting cold… Be careful not to wet a baby’s umbilical cord stump as it might get infected…(Grandmother)^2^ |  |  |
| *Recommendation of EDP to others, especially if there are siblings:* “I would definitely recommend EDP.”^27^ |  |  |
| *Supporting the partner:* “[It is] Pretty usual that the milk production decreases after a couple of weeks, it happened both times for [wife’s name]. It’s kind of difficult to ask her to fight then. Because it interrupts the night pretty much, she must get up every third hour to pump and then find there is no output.”^23^ |  |  |
| *Full-time work:* “The only advantage with the feeding tube was that we could feed her while she was sleeping. So that was an advantage during the night. We could just wake up by the clock, feed her, and go back to bed and she slept the whole time.”^23^ |  |  |
| *Struggling with new challenges in life:* I think P is more of an analyst who can sort of think a lot about things, something like why it like this is. . . she starts to worry while I’m probably the complete opposite and that sometimes causes a conflict between us.^40^ |  |  |
| Emotion satisfaction of family members: “Our communication with each other became closer and more frequent. After I did the KC, he would ask how the baby was doing. I would tell him what the nurse had taught me, how to take care of the infant, how the infant was that day, whether he was well-behaved, whether he had cried, and so on. He was also very happy to hear about these things.” (P1)^17^ |  |  |
| *Physical and psychological aspects: I* sent my husband ahead because I said, “Hey listen, I need to get some fresh air right now. I can’t go in this building just yet.” So I really felt quite bad.” (M01)^18^ |  |  |
| *Time for focusing on us:* ‘I think it is pretty much that we got time to talk, just the two of us, even though X (their sick child) was there but it was not about her, her medicine, or her spasms, it was about us’ (Mother, family 6)^43^ |  |  |
| *Opening up for a different dialogue:* We had talked, we talked to each other all the time, but I think it really made a difference to talk to someone else and to be asked questions.^43^ |  |  |
| *Learning to listen to each other:* ‘We haven’t talked about it a lot just the two of us. I think we understood a lot when we sat here and talked, he told his version, then I could tell my version, how I felt and how he felt. I thought it felt really good, we didn’t exactly do that at the hospital’ (Mother, family 9)^43^ |  |  |
| *Post-NICU transition:* Quote 6C: Like having a FICare liaison to say ‘now that you’re out we’re going to we still have all the things you can access ...We’re going to hook you up with the breastfeeding clinic to have that stuff done automatically so kind of not trying to figure out what to do so that she’s getting the best care. (Interview 1)^1^ | Support for Transitioning from NICU to Home Care |  |
| *Neonatal home care-return to everyday life:* Coming home was liberating and a step back into normal life, with more independence (M4)^12^ |  |  |
| *Neonatal home care-return to everyday life:* They got to be together as a whole family, which was really great, an incredible upside for them (F8)^12^ |  |  |
| *Facing the future:* Then I start to cry. It's like filling the house again after it's been empty (12, p. 15)^5^ |  |  |
| *Transition to home still in their thoughts:* But that’s the way it is, yes, I think that’s why, if you ask most families, they’ll say that they would rather be at home and have someone coming to check on them there instead of being in the hospital. (79)^40^ |  |  |
| *Transition before discharge*: “We are being parents for the first time with no prior experience, our baby went straight into the NICU right after birth, and we didn’t even get a chance to hold him. Having this transitional phase is valuable for us. It provides us with some understanding of how to care for him once we are back home.” (P14)^17^ |  |  |

# REFERENCES

1. Shafey A, Benzies K, Amin R, Stelfox HT, Shah V. Fathers' Experiences in Alberta Family Integrated Care: A Qualitative Study. *The Journal of Perinatal & Neonatal Nursing*. 2022;36(4):371-379. doi:10.1097/jpn.0000000000000684

2. Sarapat P FW, Jintrawet U, Mesukko J, Ray L. Perceptions and practices of parents in caring for their hospitalized preterm infants. *Pac Rim Int J Nurs Res*. 2017;21(3):220-233.

3. Abukari AS, Acheampong AK, Aziato L. Experiences and contextual practices of family-centered care in Ghanaian nicus: a qualitative study of families and clinicians. *BMC Health Services Research*. 2022/08/17 2022;22(1):1051. doi:10.1186/s12913-022-08425-0

4. Ndiaye S, Bosowski J, Tuyisenge L, et al. Parents as carers on a neonatal unit: Qualitative study of parental and staff perceptions in a low-income setting. *Early Human Development*. 2020/06/01/ 2020;145:105038. doi:<https://doi.org/10.1016/j.earlhumdev.2020.105038>

5. Lundqvist P, Westas LH, Hallstrom I. From distance toward proximity: fathers lived experience of caring for their preterm infants. *J Pediatr Nurs*. Dec 2007;22(6):490-7. doi:10.1016/j.pedn.2007.04.008

6. Maastrup R, Weis J, Engsig AB, Johannsen KL, Zoffmann V. ‘Now she has become my daughter’: parents’ early experiences of skin-to-skin contact with extremely preterm infants. *Scandinavian Journal of Caring Sciences*. 2018/06/01 2018;32(2):545-553. doi:<https://doi.org/10.1111/scs.12478>

7. Ingram J, Redshaw M, Manns S, et al. "Giving us hope": Parent and neonatal staff views and expectations of a planned family-centred discharge process (Train-to-Home). *Health Expect*. Aug 2017;20(4):751-759. doi:10.1111/hex.12514

8. Nukpezah RN, Atanuriba GA. ‘It is an emotional rollercoaster!!!’ Experiences of mothers of preterm newborns seeking care at a tertiary hospital in Ghana: a qualitative phenomenological study. *BMJ Open*. 2025;15(1):e093173. doi:10.1136/bmjopen-2024-093173

9. Jafari Z, Kermanshahi S, Vanaki Z. Communication Barriers to Implementation of Family-Centered Care from Perspective of Mothers and Personnel of Neonatal Intensive Care Unit: A Qualitative Study. *Journal of Archives in Military Medicine*. 07/16 2023;11doi:10.5812/jamm-138443

10. Jafari Z, Kermanshahi SMK, Vanaki Z, Mosayebi Z. Parental barriers to implementing family-centred care in a neonatal intensive care unit in Islamic Republic of Iran. *East Mediterr Health J*. May 29 2024;30(5):356-362. doi:10.26719/2024.30.5.356

11. Ajayi KV, Page R, Montour T, Garney WR, Wachira E, Adeyemi L. 'We are suffering. Nothing is changing.' Black mother's experiences, communication, and support in the neonatal intensive care unit in the United States: A Qualitative Study. *Ethn Health*. Jan 2024;29(1):77-99. doi:10.1080/13557858.2023.2259642

12. Lundqvist P, Weis J, Sivberg B. Parents’ journey caring for a preterm infant until discharge from hospital-based neonatal home care—A challenging process to cope with. *Journal of Clinical Nursing*. 2019/08/01 2019;28(15-16):2966-2978. doi:<https://doi.org/10.1111/jocn.14891>

13. Stephanie C. Treherne NF, Lyne Charbonneau, and Anna Axelin. Parents' Perspectives of Closeness and Separation With Their Preterm Infants in the NICU 2017;

14. Brødsgaard A, Bjerregaard M, Knudsen JB. Parents' Shared Experiences of Separation From Their Newborns After Birth in Denmark. *J Obstet Gynecol Neonatal Nurs*. Sep 2024;53(5):534-542. doi:10.1016/j.jogn.2024.04.007

15. Dien R, Benzies KM, Zanoni P, Kurilova J. Alberta Family Integrated Care™ and Standard Care: A Qualitative Study of Mothers’ Experiences of their Journeying to Home from the Neonatal Intensive Care Unit. *Global Qualitative Nursing Research*. 2022/01/01 2022;9:23333936221097113. doi:10.1177/23333936221097113

16. Finlayson K, Dixon A, Smith C, Dykes F, Flacking R. Mothers' perceptions of family centred care in neonatal intensive care units. *Sex Reprod Healthc*. Oct 2014;5(3):119-24. doi:10.1016/j.srhc.2014.06.003

17. Cai Q, Zhou Y, Chen D, Wang F, Xu X. Parental perceptions and experiences of kangaroo care for preterm infants in neonatal intensive care units in China: a qualitative study. *BMC Pregnancy and Childbirth*. 2024/07/25 2024;24(1):499. doi:10.1186/s12884-024-06622-9

18. Schmid SV, Arnold C, Jaisli S, Bubl B, Harju E, Kidszun A. Parents' and neonatal healthcare professionals' views on barriers and facilitators to parental presence in the neonatal unit: a qualitative study. *BMC Pediatr*. Apr 24 2024;24(1):268. doi:10.1186/s12887-024-04758-3

19. Kabajassi O, Reiter A, Tagoola A, et al. Facilitators and constraints to family integrated care in low-resource settings informed the adaptation in Uganda. *Acta Paediatr*. Aug 2024;113(8):1845-1851. doi:10.1111/apa.17182

20. Banazadeh M, Khanjari S, Behmaneshpour F, Oskouie F. Parental factors affecting their participation in decision-making for neonates with life-threatening conditions: A qualitative study Parents' participation in decision-making. *J Educ Health Promot*. 2024;13:316. doi:10.4103/jehp.jehp_991_23

21. Ferreira A, Ferretti E, Curtis K, et al. Parents' Views to Strengthen Partnerships in Newborn Intensive Care. Original Research. *Frontiers in Pediatrics*. 2021-September-27 2021;9doi:10.3389/fped.2021.721835

22. Neu M, Klawetter S, Greenfield JC, Roybal K, Scott JL, Hwang SS. Mothers' Experiences in the NICU Before Family-Centered Care and in NICUs Where It Is the Standard of Care. *Advances in Neonatal Care*. 2020;20(1)

23. Mӧrelius E, Brogren S, Andersson S, Alehagen S. Fathers’ experiences of feeding their extremely preterm infants in family-centred neonatal intensive care: a qualitative study. *International Breastfeeding Journal*. 2021/06/17 2021;16(1):46. doi:10.1186/s13006-021-00394-0

24. Serlachius A, Hames J, Juth V, Garton D, Rowley S, Petrie KJ. Parental experiences of family-centred care from admission to discharge in the neonatal intensive care unit. *Journal of Paediatrics and Child Health*. 2018/11/01 2018;54(11):1227-1233. doi:<https://doi.org/10.1111/jpc.14063>

25. Broom M, Parsons G, Carlisle H, Kecskes Z, Thibeau S. Exploring Parental and Staff Perceptions of the Family-Integrated Care Model: A Qualitative Focus Group Study. *Advances in Neonatal Care*. 2017;17(6)

26. Sarin E, Maria A. Acceptability of a family-centered newborn care model among providers and receivers of care in a Public Health Setting: a qualitative study from India. *BMC Health Services Research*. 2019/03/21 2019;19(1):184. doi:10.1186/s12913-019-4017-1

27. Brodsgaard A, Zimmermann R, Petersen M. A preterm lifeline: Early discharge programme based on family-centred care. *J Spec Pediatr Nurs*. Oct 2015;20(4):232-43. doi:10.1111/jspn.12120

28. Russell G, Sawyer A, Rabe H, et al. Parents' views on care of their very premature babies in neonatal intensive care units: a qualitative study. *BMC Pediatr*. Sep 13 2014;14:230. doi:10.1186/1471-2431-14-230

29. Mohammadi F, Bijani M, Cheraghi F, Kyle H. Dignity: The Cornerstone of Nursing Care Among Hospitalized Mothers of Infants in Neonatal Intensive Care Unit. *Journal of Perinatal & Neonatal Nursing*. 10/01 2020;34:E44-E50. doi:10.1097/JPN.0000000000000522

30. Heermann JA, Wilson ME, Wilhelm PA. Mothers in the NICU: Outsider to Partner. *Pediatric Nursing*. May/Jun 2005 2005;31(3):176-81, 200.

31. Wigert H, Dellenmark Blom M, Bry K. Parents’ experiences of communication with neonatal intensive-care unit staff: an interview study. *BMC Pediatrics*. 2014/12/10 2014;14(1):304. doi:10.1186/s12887-014-0304-5

32. Gotting E-K, Ulrika F, and Wigert H. Communication between parents and neonatal healthcare professionals using pictorial support when language barriers exist – parents’ experiences. *International Journal of Qualitative Studies on Health and Well-being*. 2022/12/31 2022;17(1):2122151. doi:10.1080/17482631.2022.2122151

33. Park YA, Im Y. Exploration of Family-Centered Care in NICUs: A Grounded Theory Methodology. *Qualitative Health Research*. 0(0):10497323241298928. doi:10.1177/10497323241298928

34. Franck LS, Magaña J, Bisgaard R, Lothe B, Sun Y, Morton CH. Mobile-enhanced Family Integrated Care for preterm infants: A qualitative study of parents' views. *PEC Innov*. Dec 2024;4:100284. doi:10.1016/j.pecinn.2024.100284

35. Ondusko DS, Klawetter S, Hawkins Carter E, et al. The Needs and Experiences of Black Families in the Neonatal Intensive Care Unit. *Pediatrics*. Jan 1 2025;155(1)doi:10.1542/peds.2024-067473

36. Afeadie RK, Collins A, Siaw-Marfo D, et al. Information and interaction opportunities: Experiences of mothers of babies at the neonatal intensive care unit, Accra, Ghana: A qualitative study. *Journal of Neonatal Nursing*. 2023/06/01/ 2023;29(3):554-558. doi:<https://doi.org/10.1016/j.jnn.2022.11.010>

37. Hajiaraghi N, Sadeghi N, Motaghi M, Mousavi M. Investigating the strategic elements of family-centered care in the neonatal intensive care unit: A qualitative study. *Journal of Qualitative Research in Health Sciences*. 2021;10(2):65-74. doi:10.22062/jqr.2021.193611.0

38. Dahan M, Rotteau L, Higazi S, et al. Understanding the Family Context: A Qualitative Descriptive Study of Parent and NICU Clinician Experiences and Perspectives. *Children (Basel)*. May 17 2023;10(5)doi:10.3390/children10050896

39. Shrestha T, Pandey Bista A, Joshi Pradhan S, Pudasainee-Kapri S, Subedi M. Unveiling parents’ lived experience with preterm infant care and support in neonatal care units of public hospitals in Nepal: A phenomenological inquiry. *PLOS ONE*. 2025;20(2):e0319013. doi:10.1371/journal.pone.0319013

40. Eriksson E, Pia L, and Jönsson L. Fathers’ Experiences Six Months After their Preterm Infant's Discharge from the NICU. *Comprehensive Child and Adolescent Nursing*. 2024/10/01 2024;47(4):245-254. doi:10.1080/24694193.2024.2406209

41. van den Hoogen A, Eijsermans R, Ockhuijsen HDL, et al. Parents' experiences of VOICE: A novel support programme in the NICU. *Nursing in Critical Care*. 2021/05/01 2021;26(3):201-208. doi:<https://doi.org/10.1111/nicc.12569>

42. Zahra Hadian Shirazi FS, Mahnaz Rakhshan, Narjes Pishva, Faezeh Jahanpour. Lived Experiences of the Caregivers of Infants about Family-Centered Care in the Neonatal Intensive Care Unit: A Phenomenological Study. 2018;

43. Åberg Petersson M, Persson C, Massoudi P, Benzein E, Wåhlin I. Parents' experiences of family health conversations after having a child in need of neonatal intensive care. *Scand J Caring Sci*. Dec 2021;35(4):1269-1277. doi:10.1111/scs.12945

44. Sigurdson K, Profit J, Dhurjati R, et al. Former NICU Families Describe Gaps in Family-Centered Care. *Qual Health Res*. Oct 2020;30(12):1861-1875. doi:10.1177/1049732320932897

45. Patriksson K, Stefan N, and Wigert H. Conditions for communication between health care professionals and parents on a neonatal ward in the presence of language barriers. *International Journal of Qualitative Studies on Health and Well-being*. 2019/01/01 2019;14(1):1652060. doi:10.1080/17482631.2019.1652060

46. Axelin A, Outinen J, Lainema K, Lehtonen L, Franck LS. Neonatologists can impede or support parents’ participation in decision-making during medical rounds in neonatal intensive care units. *Acta Paediatrica*. 2018;107(12):2100-2108. doi:<https://doi.org/10.1111/apa.14386>

47. Osborne AD, Worsley D, Cullen C, Martin A, Christ L. Enhancing NICU Care and Communication: Perspectives of Moderately Preterm Infant Parents. *Pediatrics*. Jun 1 2024;153(6)doi:10.1542/peds.2023-064419

48. Hadian Shirazi Z, Sharif F, Rakhshan M, Pishva N, Jahanpour F. Lived Experience of Caregivers of Family-Centered Care in the Neonatal Intensive Care Unit: "Evocation of Being at Home". *Iran J Pediatr*. Oct 2016;26(5):e3960. doi:10.5812/ijp.3960

49. Reis MD, Rempel GR, Scott SD, Brady‐Fryer BA, Van Aerde J. Developing Nurse/Parent Relationships in the NICU Through Negotiated Partnership. *Journal of Obstetric, Gynecologic & Neonatal Nursing*. 2010/11/01/ 2010;39(6):675-683. doi:<https://doi.org/10.1111/j.1552-6909.2010.01189.x>

50. Stefana A, Barlati S, Beghini R, Biban P. Fathers’ experiences of nurses’ roles and care practices during their preterm infant’s stay in the neonatal intensive care unit. *Intensive and Critical Care Nursing*. 2024/12/01/ 2024;85:103803. doi:<https://doi.org/10.1016/j.iccn.2024.103803>

51. Guttmann KF, Raviv GN, Fortney CA, Ramirez M, Smith CB. Parent Perspectives on Communication Quality in the Neonatal Intensive Care Unit. *Adv Neonatal Care*. Aug 1 2024;24(4):382-388. doi:10.1097/anc.0000000000001178

52. Weis J, Zoffmann V, Egerod I. Enhancing person-centred communication in NICU: a comparative thematic analysis. *Nurs Crit Care*. Nov 2015;20(6):287-98. doi:10.1111/nicc.12062
